# A preclinical model of peripheral T‐cell lymphoma GATA3 reveals DNA damage response pathway vulnerability

**DOI:** 10.15252/emmm.202215816

**Published:** 2022-05-05

**Authors:** Elizabeth A Kuczynski, Giulia Morlino, Alison Peter, Anna M L Coenen‐Stass, Jennifer I Moss, Neha Wali, Oona Delpuech, Avinash Reddy, Anisha Solanki, Charles Sinclair, Dinis P Calado, Larissa S Carnevalli

**Affiliations:** ^1^ Oncology R&D AstraZeneca Cambridge UK; ^2^ Immunity & Cancer Laboratory Francis Crick Institute London UK; ^3^ Oncology R&D AstraZeneca Waltham MA USA; ^4^ Peter Gorer Department of Immunobiology School of Immunology & Microbial Sciences London UK; ^5^ Present address: Benevolent AI London UK; ^6^ Present address: Translational Medicine Merck Healthcare KGaA Darmstadt Germany; ^7^ Present address: LGC Genomics Division Cambridge UK; ^8^ Present address: Flagship Pioneering Cambridge MA USA

**Keywords:** DNA damage response, GATA3, peripheral T‐cell lymphoma, syngeneic mouse model, T‐follicular helper cell, Cancer, Immunology

## Abstract

Peripheral T‐cell lymphoma (PTCL) represents a rare group of heterogeneous diseases in urgent need of effective treatments. A scarcity of disease‐relevant preclinical models hinders research advances. Here, we isolated a novel mouse (m)PTCL by serially transplanting a lymphoma from a germinal center B‐cell hyperplasia model (*Cγ1*‐Cre *Blimp1* ^fl/fl^) through immune‐competent mice. Lymphoma cells were identified as clonal TCRβ+ T‐helper cells expressing T‐follicular helper markers. We also observed coincident B‐cell activation and development of a *de novo* B‐cell lymphoma in the model, reminiscent of B‐cell activation/lymphomagenesis found in human PTCL. Molecular profiling linked the mPTCL to the high‐risk “GATA3” subtype of PTCL, showing GATA3 and Th2 gene expression, PI3K/mTOR pathway enrichment, hyperactivated MYC, and genome instability. Exome sequencing identified a human‐relevant oncogenic β‐catenin mutation possibly involved in T‐cell lymphomagenesis. Prolonged treatment responses were achieved *in vivo* by targeting ATR in the DNA damage response (DDR), a result corroborated in PTCL cell lines. This work provides mechanistic insight into the molecular and immunological drivers of T‐cell lymphomagenesis and proposes DDR inhibition as an effective and readily translatable therapy in PTCL.

## Graphical abstract

A murine peripheral T‐cell lymphoma (mPTCL) arose coincident to B‐cell hyperplasia and was developed into a transplantable preclinical model. mPTCL and human PTCL lines responded to ATR inhibition, providing a rationale for clinical exploration of DNA‐damage response inhibitors for PTCL treatment.

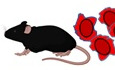

**The paper explained**

**Problem**

Peripheral T‐cell lymphoma (PTCL) represents a rare and heterogeneous cancer with a poor prognosis and limited treatment options. PTCL development is coincident to immune dysregulation, and cancer cells display phenotype characteristic of the T‐cells of origin. A major hurdle limiting therapeutic development and testing is the lack of preclinical models recapitulating the highly diverse clinical PTCL disease biology.

**Results**

We report the cellular and molecular characterization of a new murine model of PTCL (mPTCL). The mPTCL developed in a mouse model of chronic B‐cell hyperplasia and evaded host immunity as evidenced by the passage in immunocompetent syngeneic hosts. Analysis of mPTCL revealed the clonal origin and phenotypic markers that resembled the human PTCL‐GATA3 subtype with T‐follicular helper features (Tfh). At a molecular level, the mPTCL harbored a putative gain‐of‐function genetic mutation in β‐catenin, similar to that reported in a subset of human PTCLs. Moreover, the mPTCL exhibited downstream induction of Myc and hallmarks of replicative stress. This knowledge prompted the exploration of the DNA damage response inhibitor ceralasertib, which targets the ATR component of the DNA repair machinery. Human PTCL cell lines exhibited *in vitro* sensitivity to ATR inhibition, and the mPTCL displayed *in vivo* treatment responses at tolerated doses.

**Impact**

The mPTCL described and characterized herein represents a new model of relevance to human PTCL biology. This model will be instrumental in the discovery and development of new therapeutics to treat PTCL, which has a high unmet need. The work also provides a rationale for future clinical exploration of ATR inhibitors in patients with PTCL.

## Introduction

Peripheral T‐cell lymphomas (PTCLs) are a rare group of mature T‐cell malignancies in urgent need of effective treatments. PTCL displays high levels of genetic and biologic heterogeneity with 27 PTCL subtypes currently recognized (Swerdlow *et al*, 2016). The majority of cases fall under angioimmunoblastic T‐cell lymphoma (AITL), anaplastic large cell lymphoma (ALCL; ALK^+^ or ALK‐ forms), and PTCL‐not otherwise specified (PTCL‐NOS), the latter forming a catch‐all group of unclassifiable PTCL. PTCLs represent 1.6% of hematologic malignancies and ~670 new cases annually in the United Kingdom (hmrn.org); therefore, disease heterogeneity and rarity have made identifying new treatments challenging. The 5‐year survival rates for PTCL‐NOS and AITL are between 25 and 35% (hmrn.org). Anthracycline‐based CHOP chemotherapy is the standard of care, but rapid relapses are common (Schmitz & de Leval, 2017; Fiore *et al*, 2020). With the exception of brentuximab vedotin for CD30‐expressing anaplastic large cell lymphoma (ALCL), other agents including FDA‐approved pralatrexate and histone deacetylase inhibitors have not demonstrated a survival benefit (Fiore *et al*, 2020). New treatments are therefore urgently required.

Immunophenotyping and gene expression studies have established a T‐cell‐of‐origin for most PTCLs, which has elucidated divergent biologic and prognostic disease subgroups (Fiore *et al*, 2020). T‐follicular helper cells (Tfh) are considered as the origin of AITL and some related PTCL subtypes, including PTCL of Tfh immunophenotype (PTCL‐Tfh). Normal Tfh cells are crucial for germinal center (GC) responses during which B‐cells differentiate into high‐affinity antibody‐producing plasma cells (Crotty, 2011). AITL exhibits a Tfh‐like immunophenotype, BCL6 positivity, and associated with activated B‐cells, expanded follicular dendritic cell (FDC) networks, and hypergammaglobulinemia (Lunning & Vose, 2017). Coincident B‐cell lymphomas also develop in ~23% of cases (Xu *et al*, 2002; Zettl *et al*, 2002; Attygalle *et al*, 2007). Th1 or Th2 cells are considered as the origin of PTCL‐NOS (Iqbal *et al*, 2014; Wang *et al*, 2014a; Amador *et al*, 2019). One PTCL‐NOS subgroup expresses the Th1 master regulator TBX21/T‐BET and cytotoxic gene signatures (49% of PTCL‐NOS) (Szabo *et al*, 2000). Another group with an inferior prognosis expresses the Th2 master regulator GATA3 and its gene targets (33% of PTCL‐NOS) (Zheng & Flavell, 1997; de Leval *et al*, 2007; Iqbal *et al*, 2014; Wang *et al*, 2014a; Amador *et al*, 2019). These novel disease subgroups are improving understanding of the disease but have not yet altered their clinical management.

Distinct oncogenic mechanisms have been recognized in PTCL subgroups (Heavican *et al*, 2019; Watatani *et al*, 2019), supporting a tailored treatment approach for PTCL. For instance, mutations involving epigenetic regulators TET2 and DNMT3 are enriched in AITL and advocate the use of histone deacetylase inhibitors or hypomethylating agents, agents which are being explored in the clinic (Fiore *et al*, 2020). PTCL‐GATA3 often possesses PI3K/mTOR activity by gene expression and copy number alteration (Iqbal *et al*, 2010, 2014; Manso *et al*, 2016; Heavican *et al*, 2019), supporting the use of PI3K and mTOR inhibitors (Amador *et al*, 2019; Fiore *et al*, 2020). PTCL‐GATA3 is further characterized by MYC gains and overexpression, genetic loss of the CDKN2A/TP53 pathway, and possibly as consequence, genomic instability (Nakagawa *et al*, 2009; Heavican *et al*, 2019). In fact, PTCL‐GATA3 has an exacerbated DNA mutation rate, chromosomal translocations, and copy number alterations compared with PTCL‐Tfh, AITL, PTCL‐TBX21, and ALK+ ALCL (Nakagawa *et al*, 2009; Heavican *et al*, 2019; Watatani *et al*, 2019). Targeting the DNA damage response (DDR) in tumors with defective DNA repair mechanisms is a promising therapeutic approach in solid tumors and hematologic malignancies (Young *et al*, 2019; Boudny & Trbusek, 2020; Carrassa *et al*, 2020; Yap *et al*, 2021), but it has not been considered in PTCL.

Disease rarity and heterogeneity have hindered the conduct of subtype‐specific clinical trials in PTCL and aggravate the need for preclinical studies to identify new treatments. Preclinical models of PTCL are, however, scarce, with many PTCL subtypes under‐represented or lacking representative cell lines and genetic models (Ellyard *et al*, 2012; Wartewig *et al*, 2017; Cortes *et al*, 2018; Ng *et al*, 2018).

We performed an in‐depth molecular characterization of a novel mouse model of PTCL. The murine PTCL (mPTCL) was transplanted from a genetically engineered model of dysregulated germinal center B‐cells (Calado *et al*, 2010) and had a corresponding GC‐origin Tfh cell immunophenotype. mPTCL cells carried a clinically relevant mutation in β‐catenin and reflected multiple features of Th2‐like human PTCL‐GATA3 including MYC activation and genome instability. Importantly, mPTCL and human PTCL cell lines demonstrated treatment responses to the ATR inhibitor ceralasertib (AZD6738). Various ATR inhibitors are well‐tolerated in the clinic (Dillon *et al*, 2019; Yap *et al*, 2020, 2021) and thus could be a readily translatable treatment for PTCL.

## Results

### Isolation of an *in vivo* transplantable murine lymphoma

We and others previously reported that genetic deletion of *Blimp1* (*Blimp1* ^fl/fl^) specifically in GC B‐cells using the *Cγ1*‐Cre mouse line (Casola *et al*, 2006) drives B‐cell hyperplasia due to a block in plasma cell differentiation. These mice develop lymphomas mostly resembling activated B‐cell (ABC) DLBCL (Pasqualucci *et al*, 2006; Calado *et al*, 2010; Mandelbaum *et al*, 2010). We aimed to generate an immune‐competent transplantable lymphoma for preclinical investigation from this system.

**Figure 1:**
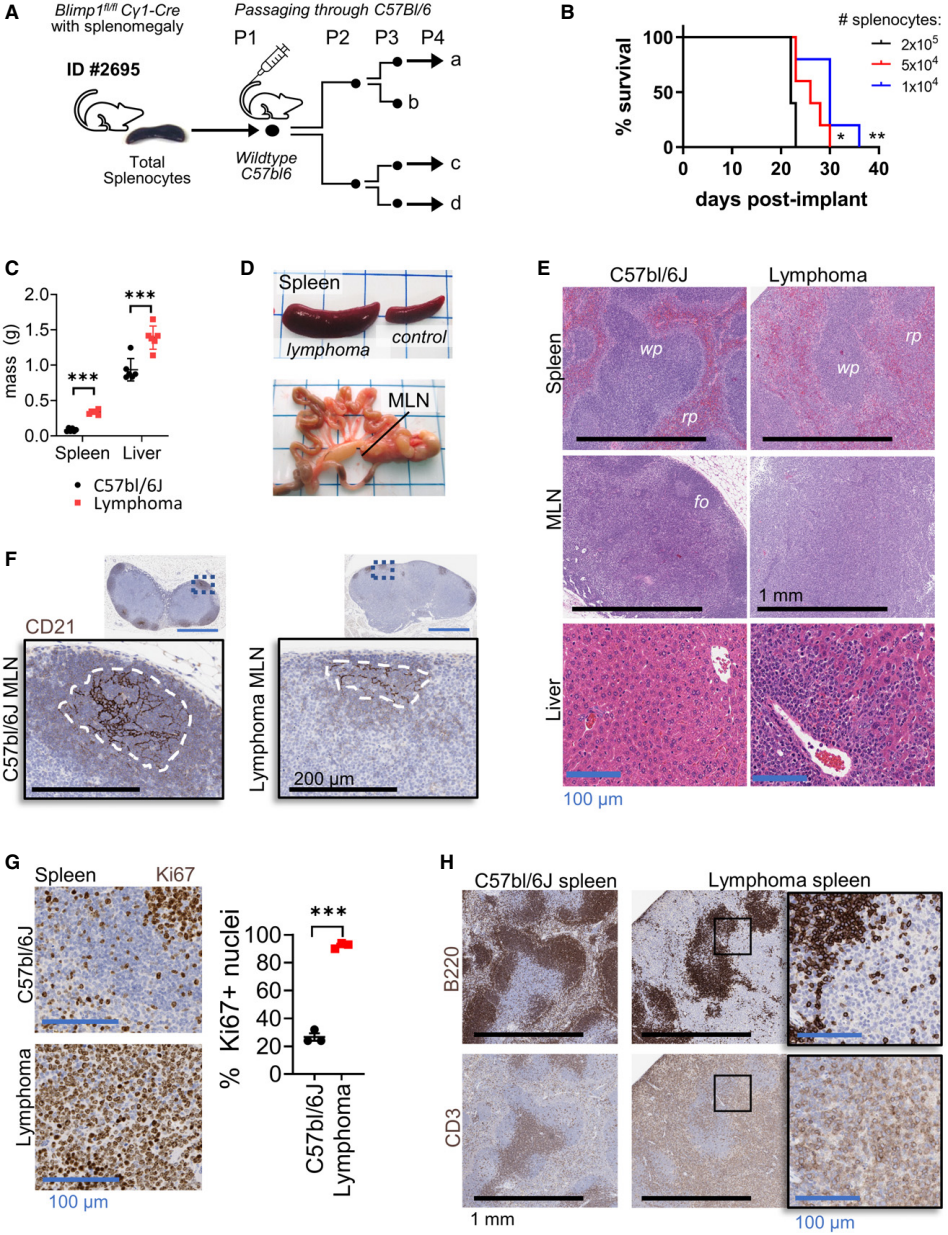
*In vivo e*xpansion of a novel transplantable disseminated lymphoma AA schematic showing the procedure by which a transplantable lymphoma line was passaged from a *Cγ1*‐Cre *Blimp1* ^fl/fl^ mouse into C57bl/6 mice. Total splenocytes were inoculated intravenously (IV) and a lymphoma serially expanded *in vivo*. Each black dot represents a single mouse passage.BTime‐to‐welfare endpoint in C57bl/6J mice intravenously injected with a varying inoculum of splenocytes (Passage (P) 5). The log‐rank test results per inoculum: 1 × 10^4^ versus 2 × 10^5^ cells = *P* = 0.008. 5 × 10^4^ versus 2 × 10^5^ cells = *P* = 0.0179.C, D(C) Mass of lymphoma‐infiltrated versus tumor‐free C57bl/6J tissues (*n* = 6 biological replicates) and (D), gross morphology of spleens and mesenteric lymph nodes (MLN) at welfare endpoint in representative mice. *T*‐test result was corrected for multiple comparisons by the Holm–Sidak method. Adjusted *P* < 0.000001 (spleen) and *P* = 0.000629 (liver). Error bars represent SEM.EH&E stain of infiltrated secondary lymphoid tissues with follicle structures (fo), red pulp (rp), and white pulp (wp) indicated in spleens.FImmunostain for CD21 in MLN labels B‐cells at a low staining intensity, and follicular dendritic cells (FDC) at a high staining intensity. IHC data in *E* and *F* are representative of 6 mice. Dotted lines represent the region magnified in the lower images. Dashed lines show the outline of the FDC network.GImmunostain for Ki67 in wildtype and lymphoma‐bearing spleen. Representative images are shown at left. The proportion of nuclei positive for Ki67 (proliferation index) is quantified at right for *n* = 3 spleens (*T*‐test *P = *0.00002331).HSequential sections of spleen stained by H&E, for B‐cells (B220) and T‐cells (CD3). The box shows an expanded region at right. *wp* = white pulp and *rp* = red pulp. *P < 0.05, **P < 0.01, ***P < 0.001.

Splenocytes from a *Cγ1*‐Cre *Blimp1* ^fl/fl^ mouse (ID #2695) that had developed splenomegaly after > 1 year (Calado *et al*, 2010) were inoculated by intravenous (IV) injection into one C57bl/6N immune‐competent mouse recipient. Within two months, the transplanted cells had engrafted and expanded in the host mouse. Splenocytes from the host mouse were subsequently passaged through a series of wildtype mice, thus establishing an aggressive lymphoma line with complete penetrance (Fig 1A). The origins of the lymphoma were traceable within the initial rounds of expansion and gave rise to four independent sub‐lineages hereon referred to as “a”, “b”, “c” and “d” (Appendix Fig S1). Transplantation of as few as 10,000 splenocytes (P4) led to aggressive disease in 100% of mice after 4–5 weeks (Fig 1B). However, the lymphoma was unable to survive *ex vivo* in standard culture conditions. Mice at the welfare endpoint showed a consistent pattern of spleen, liver, and mesenteric lymph node (MLN) enlargement (Fig 1C and D). Histological analysis of enlarged tissues showed diffuse infiltration with atypical lymphocytes (Fig 1E) that expanded the splenic red pulp and depleted follicle structures and FDC networks in the spleen and MLN (Fig 1E and F). These cells were also concentrated in liver portal tracts, bone marrow, and alveolar walls of the lung (Fig 1E, Appendix Fig S2A and B) and had a high Ki67‐index indicative of malignant proliferation (Fig 1G).

### Characterization of *Cγ1*‐Cre *Blimp1* ^fl/fl^‐derived T‐cell transplantable lymphoma

**Figure 2:**
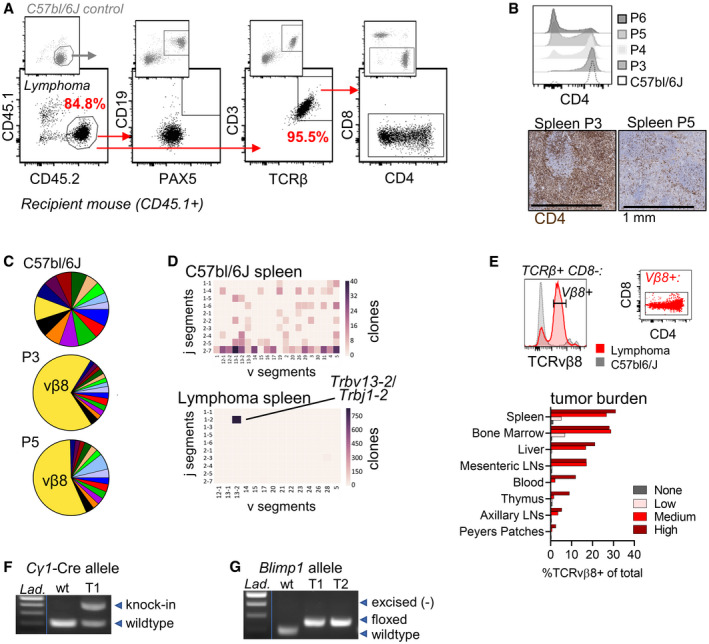
The lymphoma originated from a single positive T‐cell clone in a *Cγ1*‐Cre *Blimp1* ^fl/fl^ mouse AP3 splenocytes (5 × 10^5^) were implanted IV into congenic CD45.1+ mice and resultant lymphomas that developed were evaluated for expression of B‐ and T‐cell lineage markers by flow cytometry. Top plots are of a control C57bl/6J (CD45.2^+^) spleen. Bottom plots show immunophenotype of splenocytes from a CD45.1 mouse with an expanded CD45.2^+^ lymphoma. The data are representative of five animals.BFlow cytometry analysis of CD4 expression on lymphomas cells from four mice at different passages (P3‐P6). In top panel, CD8^−^TCRβ^+^ cells are gated. IHC for CD4 in P3 and P5 spleens. Each IHC image is representative of 3 mice.CFlow cytometric TCRvβ repertoire analysis of whole spleens from two lymphoma passages. Each color represents the relative usage of a single TCRvβ on total Thy1^+^CD3^+^ T‐cells containing a mix of lymphoma cells and host T‐cells. The percent of positive stained cells was normalized to the sum of total TCRvβ^+^ cells. Data are representative of two experiments and all passages.DT‐cell transcript complementary determining region 3 (CDR3) sequencing of C57bl/6J (top) and lymphoma‐bearing spleen. The chord diagram shows numbers of distinct TRBV and TRBJ clones. The dominant CDR3 sequence in the lymphoma is CASGDTQANSDYTF. *Trbv13‐2* gene corresponds to TCRvβ8.2 protein.ECD8‐TCRβ8^mid^ T‐cell quantification across lymphoid and nonlymphoid tissues. Samples were collected from three lymphoma‐bearing mice with low, medium, and high tumor burden. Data are compared to that of a lymphoma‐free mouse (tumor burden = “none”). The CD4 and CD8 expression pattern of gated TCRvβ8^+^ cells is shown in the dot plot at right. Sizes of tumor dissemination was confirmed in three independent experiments.F, G(F) PCR for *Cγ1*‐Cre and wildtype *Cγ1* alleles and (G), wildtype, floxed, or excised *Blimp1* alleles in DNA from wildtype (wt) control C57bl/6J cells and purified T‐cells or CD45.2^+^ cells from mouse with lymphoma (T1, T2). PCR data were confirmed in at least three independent tests. Lad. = ladder.

*Cγ1*‐Cre *Blimp1* ^fl/fl^ mice typically succumb to B‐cell hyperplasia that resembles features of ABC‐DLCBL over an extended period of time (> 300 days) (Calado *et al*, 2010; Mandelbaum *et al*, 2010). Unexpectedly, B220^+^ B‐cells were scarce in transplanted lymphoma sections (Fig 1H) whereas CD3 T‐cell stain was ubiquitous (Fig 1H, Appendix Fig S2B). To confirm hematopoietic lineage, lymphoma cells (CD45.2^+^) were transplanted into CD45.1^+^ congenic mice and tissues analyzed by flow cytometry. CD45.2^+^ lymphoma cells were negative for all evaluated B‐cell markers, including CD19, CD20, and Pax5, and instead expressed T‐cell markers CD3, Thy1, and TCRβ (Fig 2A and Appendix Fig S2C). CD45.2^+^ cells were negative for CD8 cytotoxic T‐cell marker but expressed varied levels of CD4 helper T‐cell marker. Tracking the evolution of successive *in vivo* passages attributed the aberrant CD4 expression to a gradual loss of CD4 during passaging (Fig 2B). The origin of the mouse transplantable lymphoma was therefore of mature CD4^+^ T‐helper cell origin, reflecting a PTCL.

Human (h)PTCLs derive from a clonal origin as indicated by frequent clonal TCRα/β rearrangements (van Dongen *et al*, 2003). By assaying the TCRβ variable chain repertoire, we found TCR Vβ8 to dominate across passages (Fig 2C). TCRβ complementary determining region 3 (CDR3) sequencing showed extremely biased variable (V) and joining (J) segment usage (Fig 2D) confirming tumor clonality. TCR Vβ8^‐^‐expressing T‐cells were readily detected in diseased tissues and could thus indicate disease burden (Fig 2E).

*BLIMP1* loss is found in a fraction of hPTCL (Calado *et al*, 2010; Heavican *et al*, 2019). Therefore, we considered the possibility of ectopic Cre‐mediated recombination of *Blimp1* in T‐cells in the *Cγ1*‐Cre *Blimp1* ^fl/fl^ mouse. Genotyping showed that mPTCL cells carried the *Cγ1*‐Cre allele (Fig 2F). However, cells were homozygous for the floxed *Blimp1* allele, demonstrating that *Blimp1* had not been excised (Fig 2G). Taken together, the mPTCL originated from a clonal bystander CD4^+^ T‐cell with intact *Blimp1* in a *Cγ1*‐Cre *Blimp1* ^fl/fl^ mouse (Calado *et al*, 2010).

### mPTCL phenotypically and functionally resemble T‐follicular helper cells

**Figure 3:**
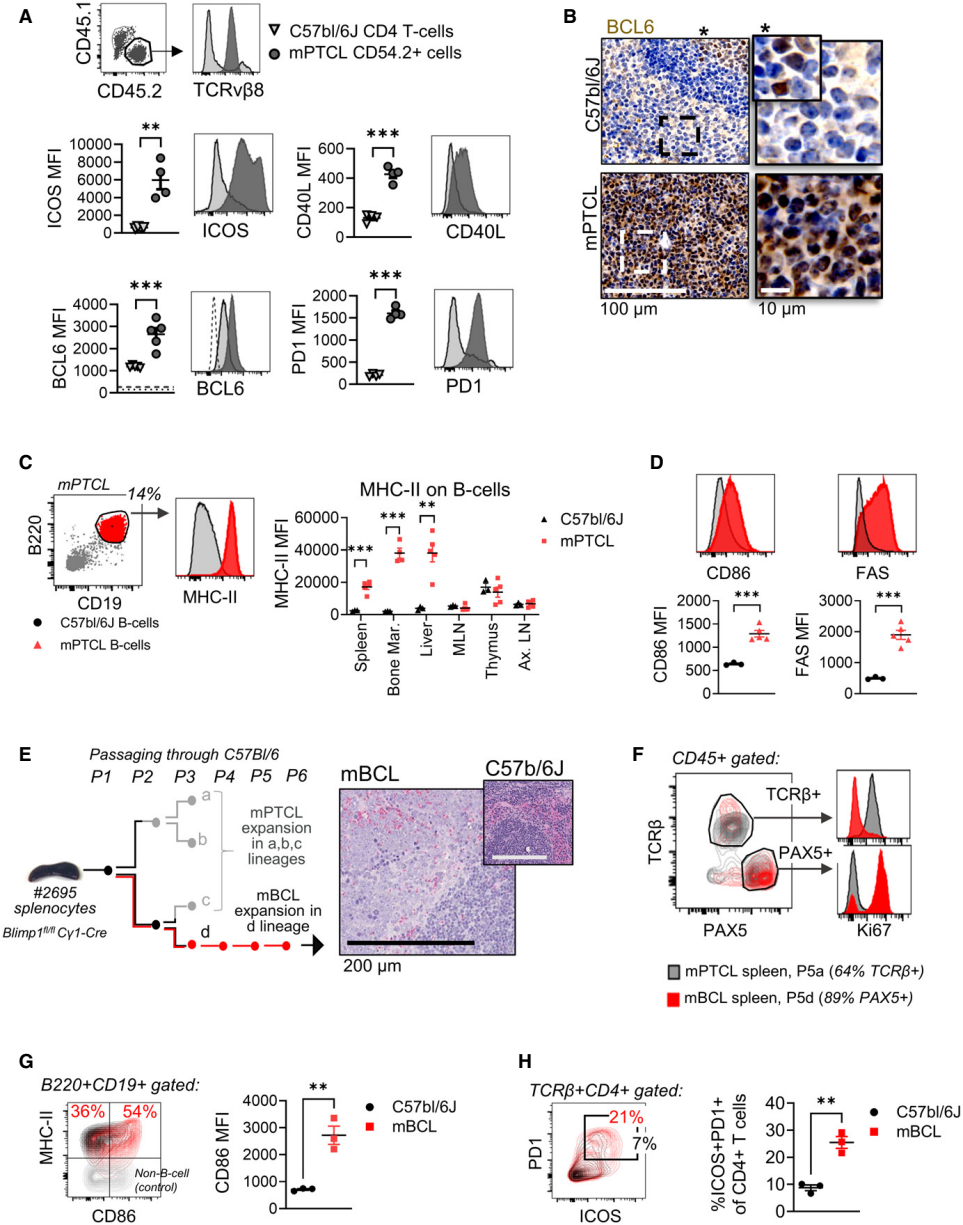
mPTCL has a T‐follicular helper cell‐like phenotype and associates with activated B‐cells AmPTCL splenocytes from passage 3 were transplanted into CD45.1 congenic C57bl6/J mice. Host mice were sacrificed at welfare endpoint between days 31 and 36 post‐transplantation and spleens analyzed by flow cytometry. CD45.2^+^ lymphoma cells were gated and compared with C57bl6/J CD4^+^ T‐cells (*n* = 4). TCRvβ8^+^CD8^−^ T‐cells are gated for BCL6 analysis (*n* = 5; shown against isotype control in dashed line). *T*‐test *P* = 0.0021 (ICOS), *P* = 0.00005723 (CD40L), *P* = 0.0008 (BCL6), *P* = 0.00000067 (PD1). Bars represent SEM.BImmunohistochemistry for BCL6 in a C57bl/6J versus mPTCL spleen (*rp* = red pulp). The magnified images at right represent the regions enclosed in dashed boxes. In the images from C57bl/6J mice (top subpanels), a normal germinal center with positive nuclear BCL6 staining is shown by an asterix and magnified in the inset. Data are representative of 8 mice.CExpression levels of MHC‐II on CD45^+^CD19^+^B220^+^ host B‐cells from tissues collected from C57bl/6J (*n* = 3) and mPTCL‐bearing mice (*n* = 5). Example dot plot of B‐cell gating in an mPTCL spleen is shown in the subpanel at left. *T*‐test results were corrected for multiple comparisons by the Holm–Sidak method. Adjusted *P* = 0.002323 (spleen), *P* = 0.001133 (bone marrow), *P* = 0.013809 (liver), *P* = 0.604980 (mesenteric lymph node, MLN), *P* = 0.762589 (thymus), *P* = 0.762589 (axillary lymph node, Ax LN). Bars represent SEM.DExpression levels of CD86 and FAS on splenic B‐cells from mice in panel *C*. *T*‐test *P* = 0.0004 (CD86), *P* = 0.0004 (FAS). Results were confirmed in three independent experiments. Bars represent SEM.EA schematic showing how a distinct B‐cell lymphoma (mBCL) was derived from lineage “d” of passaged lymphomas. Lineages “a”, “b”, and “c” resulted in only T‐cell lymphomas (mPTCL). H&E stain of a P7 spleen (representative of 5 mice) infiltrated with mBCL and compared with a wildtype C57bl/6J spleen (inset, scale = 200 µm).FProliferation as assessed by Ki67 stain levels in T‐cells (TCRβ^+^/PAX^−^) versus B‐cells (TCRβ‐/PAX^+^) from P5a (mPTCL) versus P5d (mBCL) spleens. Data are representative of five lymphomas.G, H(G) Activation marker expression on CD19^+^B220^+^ B‐cells and (H) on bystander CD4^+^ T‐cells from P6d mBCL and wildtype spleens (*n* = 3). CD86 on B‐cells/mBCL: *P = *0.0041. On T‐cells: *P* = 0.0024. Results are representative of three independent *in vivo* experiments. MFI=mean fluorescence intensity. **P < 0.01, ***P < 0.001.

On the basis that *Cγ1*‐Cre *Blimp1* ^fl/fl^ mice develop dysregulated GC B‐cells (Calado *et al*, 2010), we surmised that the crosstalk with a Tfh cell may play a role in T‐cell transformation. mPTCL cells displayed expression of the surface Tfh markers ICOS, PD1, and CD40L. Importantly, cells also expressed BCL6, the master transcription regulator of Tfh cell differentiation (Johnston *et al*, 2009; Nurieva *et al*, 2009) (Fig 3A). Immunostaining confirmed nuclear BCL6 expression throughout mPTCL‐infiltrated spleens (Fig 3B). Thus tumor cells expressed several markers of normal Tfh cells. The homogeneity of PD1 and ICOS on tumor cells and their maintained expression throughout passaging (Appendix Fig S3A and B) suggested a possible Tfh cell origin. However, since tumor cells were negative for the Tfh chemokine receptor CXCR5 (Appendix Fig S3C and D), a definitive Tfh origin could not be concluded.

AITL and related PTCL‐Tfh is pathologically associated with B‐cell activation and dysfunction (Xu *et al*, 2002; Attygalle *et al*, 2007; Hoffmann *et al*, 2016; Lunning & Vose, 2017). We found that B‐cells were relatively depleted in tumor‐infiltrated spleens (Appendix Fig S3E). Yet, B‐cells across infiltrated tissues had a highly activated phenotype (CD86^hi^ MHC‐II^hi^ Fas^hi^; Fig 3C and D), reminiscent of hPTCL. We did not detect GC B‐cell expansion, immunoglobulin class‐switching, and hypergammaglobulinemia (Appendix Fig S3F–I). Plasma cell frequencies were only modestly increased in the spleen (Appendix Fig S3J).

An occasional feature of hPTCL is the development of a co‐occurring B‐cell lymphoma (Xu *et al*, 2002; Zettl *et al*, 2002; Attygalle *et al*, 2007; Wang *et al*, 2014b; Hoffmann *et al*, 2016). We observed that in one series of passages in mice (Lineage “d”), the mPTCL was overtaken by a lethal B‐cell lymphoma (mBCL; Fig 3E and F and Appendix Figs S1 and S3K). We traced the lymphoma to a mouse receiving the first passage (P1) and the absence of *Cγ1*‐Cre *Blimp1* ^fl/fl^ transgenic alleles confirmed it had arisen spontaneously in wildtype recipient mice (Appendix Fig S3L). The mBCL cells had an activated phenotype (MHC‐II^+^ CD86^hi^; Fig 3G), and although frequencies of associated resident CD4^+^ T‐cells declined with mBCL progression (Appendix Fig S3M), a high proportion was of Tfh‐like phenotype (TCRβ^+^ ICOS^hi^ PD1^hi^; Fig 3H). Thus, mPTCL phenotypically and functionally resembled Tfh cells, and Tfh and B‐cell activation were prominent features of passaged lymphomas.

### mPTCL has a Tfh/Th2‐cell phenotype and resembles PTCL‐GATA3

**Figure 4:**
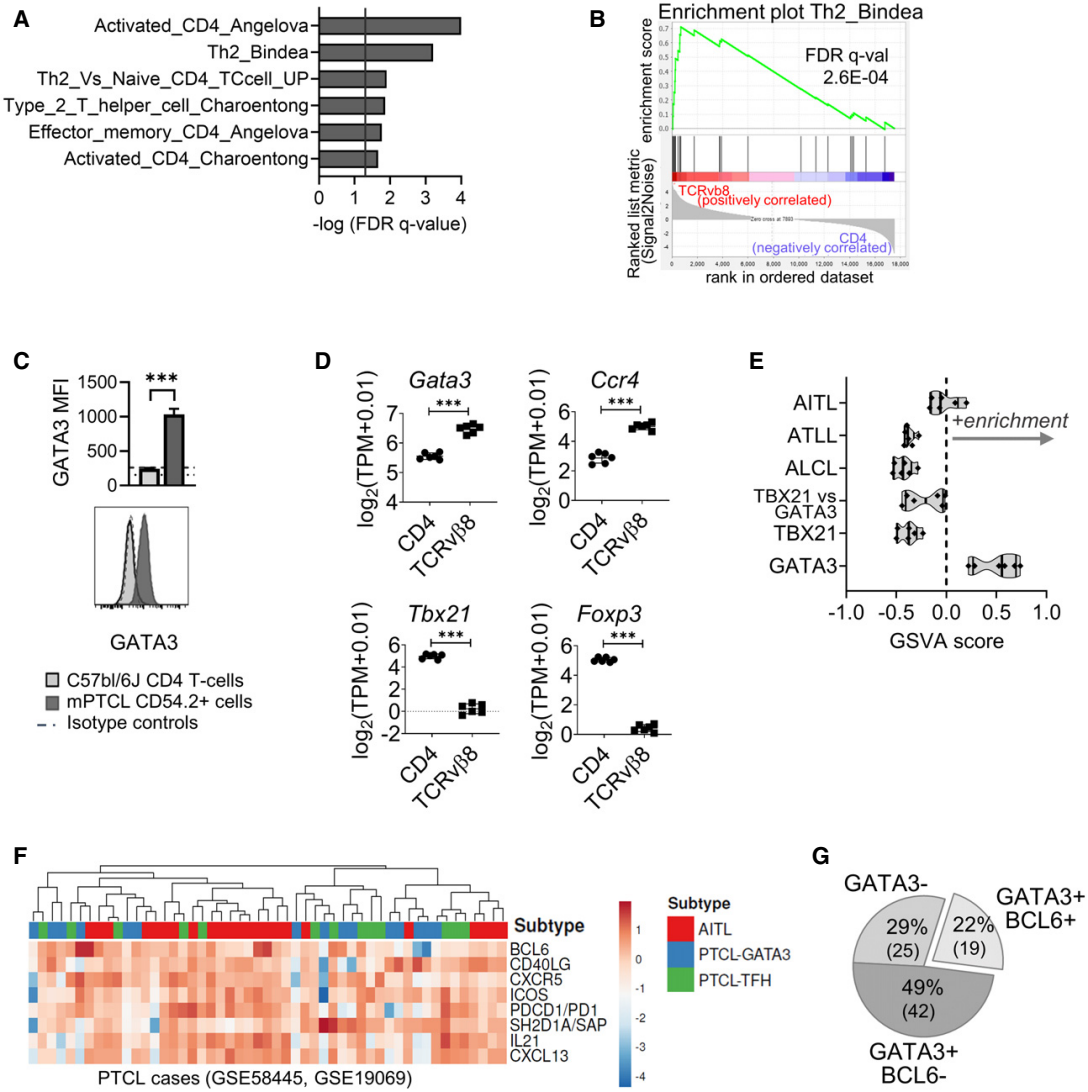
mPTCL overexpresses GATA3 and Th2 genes, resembling molecular defined PTCL‐GATA3 FACS‐sorted TCRβ^+^ CD8^−^ TCRvβ8^mid^ ICOS^hi^ mPTCL cells and C57bl/6J CD4^+^ T‐cells were RNA sequenced. Differential expression of T‐cell subset‐associated genes were analyzed (*n* = 6). GSEA analysis of 23 gene signatures, representative of Th1, Th2, Th17, Tfh, Treg, T effector memory, and T central memory cells. Significantly enriched signatures are plotted (where *q* < 0.05—indicated by the vertical line). FDR Q‐values can be found in Dataset EV2.Gene enrichment plot for the Th2_Bindea gene signature.Expression levels of GATA3 in wildtype CD4^+^ T‐cells versus CD45.2^+^ gated PTCL cells expanded in CD45.1 congenic hosts (*n* = 4). *P* = 7.19E‐05. Representative histograms are shown against an isotype‐stained mPTCL control (dashed line). Dotted line represents the isotype control stain for CD4 T‐cells. Data are representative of three experiments. Bars represent SEM.Average log2 expression levels of Th2 (*Gata3, Ccr4*), Th1 (*Tbx21*), and Treg (*Foxp3*) genes in purified TCRvβ8^+^ mPTCL cells versus wildtype C57bl/6J CD4^+^ T‐cells (*n* = 6). Adjusted *P* = 5.08E‐07 (*Gata3*), *P* = 9.38E‐07 (*Ccr4*), *P* = 2.87E‐08 (*Tbx21*), *P = *6.75E‐10 (*FoxP3*).The transcriptome of mPTCL spleens (*n* = 6) were analyzed by gene set variance analysis (GSVA) for enrichment of representative genes of molecular defined human PTCL subgroups (Iqbal *et al* 2014). A positive score indicates enrichment in a single sample of mPTCL‐infiltrated spleen. Scores are shown in truncated violin plots in which vertical lines represent the 1^st^ quartile, the median, and the 3^rd^ quartile of the data.Log2‐normalized expression of Tfh immunophenotypic markers in 51 cases of human PTCL from previously published datasets, with hierarchical clustering by PTCL subtype. “PTCL‐Tfh” represents “nodal PTCL with Tfh phenotype.”The GATA3 and BCL6 expression status of 86 cases of human PTCL‐NOS as assessed by IHC using data published in Watatani *et al* (2019). ***P < 0.001.

Gene expression profiling is increasingly used to stratify PTCL subtypes and their underlying biology (Fiore *et al*, 2020). We therefore performed RNAseq analysis on FACS‐purified TCR Vβ8^+^ mPTCL cells and investigated enrichment for signatures relative to CD4^+^ T‐cells. We observed mPTCL cells to be enriched for genes of Th2 cells, activated and effector memory T‐cells (FDR < 0.05; Fig 4A and B). An activated, memory‐like phenotype may be a broad feature across PTCL (Rüdiger *et al*, 2006; Pechloff *et al*, 2010; Wang *et al*, 2011), and to this point, the mPTCL highly expressed CD44 (Appendix Fig S4A–C). Further, the mPTCL cells were modestly rescued *in vitro* by the naïve/memory T‐cell homeostatic cytokine IL‐7 (Barata *et al*, 2019) and highly expressed IL‐7 co‐receptor IL7Rα (Appendix Fig S4D–F). We also found that the mPTCL expressed the Th2‐cell master regulator GATA3 (Zheng & Flavell, 1997) (Fig 4C) and stained cells homogenously, supporting Th2‐cell biology across tumor cells (Appendix Fig S4G). *Gata3* and Th2‐associated chemokine receptor *Ccr4* transcripts were significantly increased in mPTCL cells relative to naïve CD4^+^ T‐cells, whereas levels of Th1 (*Tbx21*) and Treg (*FoxP3*) master regulators transcripts were reduced (Fig 4D). Further, genes previously identified as upregulated in hPTCL‐GATA3 versus other hPTCL subtypes (Iqbal *et al*, 2014) were over‐represented in mPTCL spleens by GSVA analysis (Fig 4E, Appendix Fig S4H).

There are anecdotal reports that a subset of PTCL‐GATA3 co‐express Tfh markers (Watatani *et al*, 2019; Drieux *et al*, 2020), a phenotype similar to that of mPTCL (Figs 3A and 4C). Using a published gene expression dataset of 51 cases of hPTCL (Iqbal *et al*, 2010, 2014), hierarchical clustering indicated that molecular‐classified PTCL‐GATA3 could not be reliably distinguished from nodal PTCL‐Tfh or AITL on the basis of Tfh marker gene expression (Fig 4F). Further analysis of published PTCL‐NOS IHC data (Watatani *et al*, 2019) demonstrated co‐expression of BCL6 with GATA3+ was frequent, observed in 22% of PTCL‐NOS (Fig 4G). Thus, the data suggested that the mPTCL modeled a clinically relevant PTCL of dual Tfh/Th2 tumor phenotype.

### Molecular characterization of mPTCL reveals potential genetic drivers and hallmarks of genomic stress

**Figure 5:**
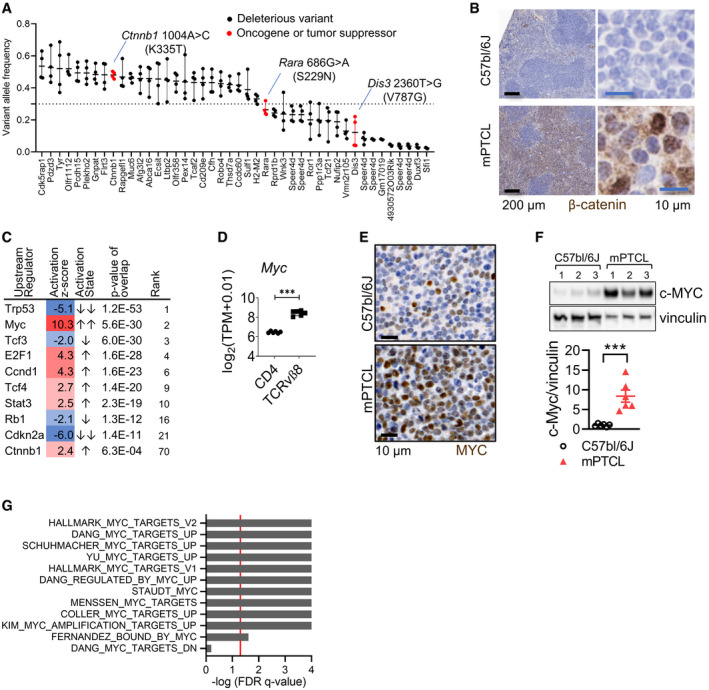
mPTCL has an oncogenic β‐catenin mutation and overactive MYC Allele frequencies of predicted deleterious mutations detected by exome sequencing of mPTCL T‐cells (*n* = 4). Deleterious variants have a SIFT (sorting intolerant from tolerant) score ≤ 0.05. In red are OncoKB‐listed oncogenes or tumor suppressors. Each oncogene in red has a SIFT score = 0, indicating high likelihood to be deleterious. Bars represent SEM. The dotted line represents the lower allele frequency cutoff for clonal heterozygous variants.IHC for β‐catenin in spleens (left) and magnified regions of mesenteric lymph node (right) in a normal C57bl/6J mouse and a mouse with mPTCL. Images are representative of six mice.Ingenuity pathway analysis predicted upstream regulators in purified TCRvβ8^+^ mPTCL versus CD4^+^ T‐cells (*n* = 6). A selection of upstream regulators is shown. The full set can be found in Dataset EV4. *Z*‐score ≥ 2 or ≤ 2 represents predicted activation or inhibition of a regulator, respectively.Log2‐transformed expression levels of *Myc* transcript in TCRvβ8^+^ versus wildtype CD4^+^ T‐cells (*n* = 6). Adjusted *P* = 5.78E‐08. *P*‐values were calculated by ROSALIND.Immunostain for c‐MYC in mPTCL versus C57bl/6J spleens (representative of three mice).Western blot for c‐MYC in whole spleen lysates from control C57bl/6J and mPTCL mice. Quantification of c‐MYC expression normalized to vinculin housekeeper gene levels (*n* = 6). *T*‐test *P* = 0.0007. Bars represent SEM.GSEA for the top 12 (of 16) enriched MYC‐regulated gene signatures in purified TCRvβ8^+^ lymphoma cells. Red line indicates FDR *q* = 0.05. FDR Q‐values can be found in Dataset EV3. *** *P < *0.001.

Approximately half of PTCLs carry mutations involving TCR signaling pathways (Schatz *et al*, 2015; Vallois *et al*, 2016). PTCL‐GATA3 in particular often displays loss or mutation of the CDKN2A/B‐TP53 axis and gains/amplifications in STAT3 and MYC (Heavican *et al*, 2019; Watatani *et al*, 2019). To understand whether similar genomic alterations contributed to mPTCL transformation, the lymphoma cells were profiled by exome sequencing. We identified 44 genes carrying deleterious somatic mutations that were common to four mPTCL samples (Fig 5A and Dataset EV6). Among these were three known oncogenes *Ctnnb1* (encoding β‐catenin), *Dis3,* and *Rara*. Low allele frequencies (< 0.3) suggested that *Dis3* and *Rara* mutations were subclonal, whereas a single copy of *Ctnnb1* 1004A>C (K335T substitution) with an allele frequency of 0.48 was likely to be clonal and thus possibly involved in tumor initiation (Fig 5A, Appendix Fig S5A). Whilst β‐catenin is critical for T‐cell differentiation and activation (Yu *et al*, 2009; Lovatt & Bijlmakers, 2010; van Loosdregt & Coffer, 2018), its dysregulation may also be oncogenic in T‐cells (Chiarini *et al*, 2020). Notably, *CTNNB1* mutations including exon 7 K335T were previously identified in 6% of PTCL (Vallois *et al*, 2016). Since the β‐catenin amino acid sequence is 100% conserved between mouse and human (Appendix Fig S5B), these data suggested that a biologically and clinically relevant oncogenic *Ctnnb1* K335T mutation arose spontaneously in a mouse T‐cell clone, possibly contributing to cellular transformation. In contrast, mutation or copy number alterations impacting MYC‐CDKN2A/B‐TP53 and PTEN‐PI3K axes, frequent in PTCL‐GATA3 (Heavican *et al*, 2019; Watatani *et al*, 2019), were not found in the mPTCL (Dataset EV7).

β‐catenin levels are regulated by a cytosolic destruction complex that when inactivated, causes β‐catenin stabilization, nuclear translocation, and target gene regulation with LEF/TCF co‐factors (van Loosdregt & Coffer, 2018). *Tcf3* and *Tcf7* were upregulated or at equal levels, respectively, in mPTCL relative to CD4^+^ T‐cells (Appendix Fig S5C). K335 mutations impair the binding of adenomatous polyposis coli (APC), a component of the destruction complex (Appendix Fig S5D), increasing β‐catenin pools, and its transcriptional activity (Pilati *et al*, 2014; Rebouissou *et al*, 2016; Liu *et al*, 2020). We found that β‐catenin protein and transcript were markedly upregulated in mPTCL‐infiltrated tissues (Fig 5B, Appendix Fig S5C) and nuclear localization was frequent, demonstrating heightened β‐catenin activation (Pilati *et al*, 2014; Rebouissou *et al*, 2016).

MYC is a key transcriptional target of wildtype and oncogenic β‐catenin (He *et al*, 1998; Barker *et al*, 2000) and plays an important role in PTCL‐GATA3 pathobiology (Iqbal *et al*, 2010, 2014; Manso *et al*, 2016; Heavican *et al*, 2019). Supporting a possible causative role for MYC and β‐catenin in mPTCL pathogenesis, ingenuity pathway analysis (IPA) identified MYC as the top activated upstream regulator (*Z*‐score = 10.3; *P* of overlap = 5.3 × 10^30^) followed by the cell cycle regulators E2F1, CCND1, TCF4, and β‐catenin among others (Fig 5C and Dataset EV4). Consistent with these analysis, *Myc* transcripts were significantly upregulated in mPTCL compared with wildtype CD4^+^ T‐cells (Fig 5D) as was MYC protein in the spleen by IHC and Western blot analysis (Fig 5E and F). GSEA analysis for gene signatures of activated MYC further indicated overactivity in mPTCL (Fig 5G). Notably, the analysis also showed reduced TP53, RB1, and CDKN2A activity (Fig 5C), echoing the loss of function of the CDKN2A/B‐TP53 axis in PTCL‐GATA3 (Heavican *et al*, 2019; Watatani *et al*, 2019). In summary, these data demonstrate that mPTCL displays molecular features found in human PTCL‐GATA3.

### ATR is a therapeutic target in PTCL

**Figure 6:**
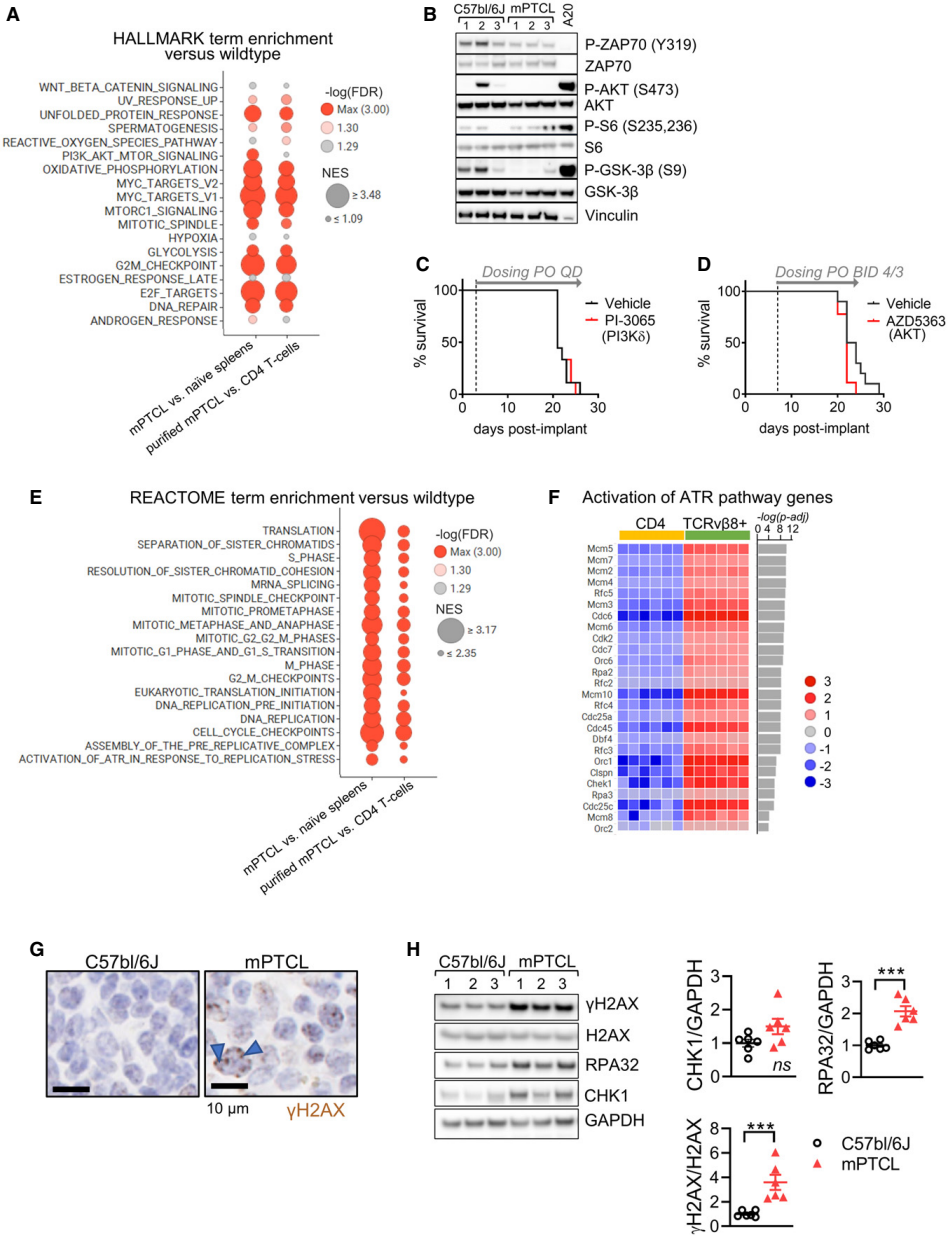
Interrogation of potential targets for therapeutic intervention in mPTCL AGSEA of HALLMARK gene signature was performed on whole mPTCL‐infiltrated versus wildtype spleens and purified TCRvβ8^+^ mPTCL versus wildtype CD4^+^ T‐cells (*n* = 6). Eighteen of the top 20 positive enriched signatures that were identified in whole spleen and purified cells are shown. Red circles depict significant enrichment (FDR *q*‐value < 0.05). NES—normalized enrichment score. FDR Q‐values can be found in Dataset EV5.BWestern blot for phosphorylated and total ZAP70, AKT, S6, and GSK3β in lysates from three control (C) C57bl/6J and three mPTCL (T) spleens. Murine DLBCL cell line A20 is a positive control for constitutively active AKT signaling.C, D(C) Kaplan–Meyer curve showing time to welfare endpoint (survival) in mice implanted IV with 10^4^ mPTCL cells and dosed with PI3Kδ inhibitor PI‐3065 at 75 mg/kg PO QD (*n* = 10) (log‐rank test *P* > 0.9999) or (D), the pan‐AKT inhibitor capivasertib (AZD5363) dosed at 130 mg/kg PO BID 4 days on/3 days off (4/3; *n* = 9) versus vehicle control (*n* = 10) (log‐rank test *P* = 0.0498 in favor of the control arm). Results in *C* and *D* are representative of three independent studies of PI3K pathway inhibitors.EGSEA of REACTOME terms in the comparison of transcriptome from whole spleens and purified cells as in (A). A selection of 18 of the top 35 pathways are shown (all with FDR *P*‐value < 0.05). FDR Q‐values can be found in Dataset EV5.FHeatmap of log2 normalized expression of significantly upregulated genes (*P*‐adjusted < 0.05) in “REACTOME Activation of ATR in response to replication stress” gene signature from panel *E*.GImmunostain for DNA damage marker γH2AX in wildtype and mPTCL spleens. mPTCL data are representative of six mice. Arrowhead indicates positive nuclear foci.HWestern blot for γH2AX, total H2AX, CHK1, and RPA32 in control C57bl/6J and mPTCL spleens. GAPDH is a loading control. Three representative samples per group are shown in the blot (left), and quantification was performed on *n* = 6 at right. *T*‐test *P* = 0.0798 (CHK1; not statistically significant, ns), *P = *8.69E‐05 (RPA32), *P* = 2.11E‐03 (γH2AX). ***P < 0.001.

We next used the mPTCL model to evaluate treatment strategies for possible clinical translation. Transcriptomes of mPTCL were queried to identify vulnerabilities potentially amenable to pharmacologic intervention. Similar to human PTCL‐GATA3 (Iqbal *et al*, 2010, 2014; Manso *et al*, 2016; Heavican *et al*, 2019), the mPTCL showed a modest but significant enrichment for HALLMARK PI3K/AKT/mTOR signature genes (Fig 6A). PI3K/mTOR signaling propagates TCR and co‐stimulatory signals that contribute to the pathogenesis of certain PTCL (Vallois *et al*, 2016; Horwitz *et al*, 2018). Yet, we did not find evidence of activated proximal TCR signaling or constitutive AKT signaling in untreated mPTCL spleen lysates as assessed by ZAP70 (Y319), AKT (S473), S6 (S235,S236), and GSK3β (S9) phosphorylation levels (Fig 6B). In agreement, administration of inhibitors of PI3Kδ (PI‐3065; Fig 6C) or AKT (AZD5363, capivasertib; Fig 6D) failed to prolong the survival of mice with mPTCL.

More prominent than AKT/mTOR was the enrichment in the mPTCL for signatures related to cell cycle regulation, DNA replication, and the DNA damage response (DDR; Fig 6A and E). MYC can induce sustained proliferation, which in turn leads to replication stress and generation of extended regions of single‐stranded (ss)DNA (Dominguez‐Sola *et al*, 2007; Dominguez‐Sola & Gautier, 2014; Kotsantis *et al*, 2018). If not effectively resolved by the replication stress response, a DDR pathway regulated by ataxia telangiectasia and Rad3‐related (ATR), ssDNA can be cleaved, generating DNA double‐strand breaks (DSBs) and replication fork collapse (Forment & O'Connor, 2018). Consistent with heightened MYC activity (Fig 5), genes involved in the ATR response to replication stress were upregulated in lymphoma‐infiltrated spleens and in purified tumor cells relative to controls (Fig 6E and F). mPTCL further showed evidence of accumulating DNA DSBs and genome instability, marked by γH2AX‐positive nuclear foci (Fig 6G) and significantly elevated γH2AX in spleen lysates (Fig 6H). RPA32 (S33), ATR (S428), and CHK1 (S345) were not constitutively phosphorylated/activated in mPTCL samples (Appendix Fig S6A); however, the total levels of RPA32 and CHK1 were increased in malignant spleens (Fig 6H). An analysis of Cancer Cell Line Encyclopedia data showed that, like mPTCL, human T‐cell lymphoma lines tend to express higher levels of *RPA2* (RPA32) and *CHEK1* compared with all cancer cell lines overall (Appendix Fig S6B). Thus, the mPTCL displayed evidence of endogenous DDR activation and both mPTCL and human T‐cell lymphomas upregulate ATR pathway genes, a possible indicator of the ongoing response to replication stress.

**Figure 7:**
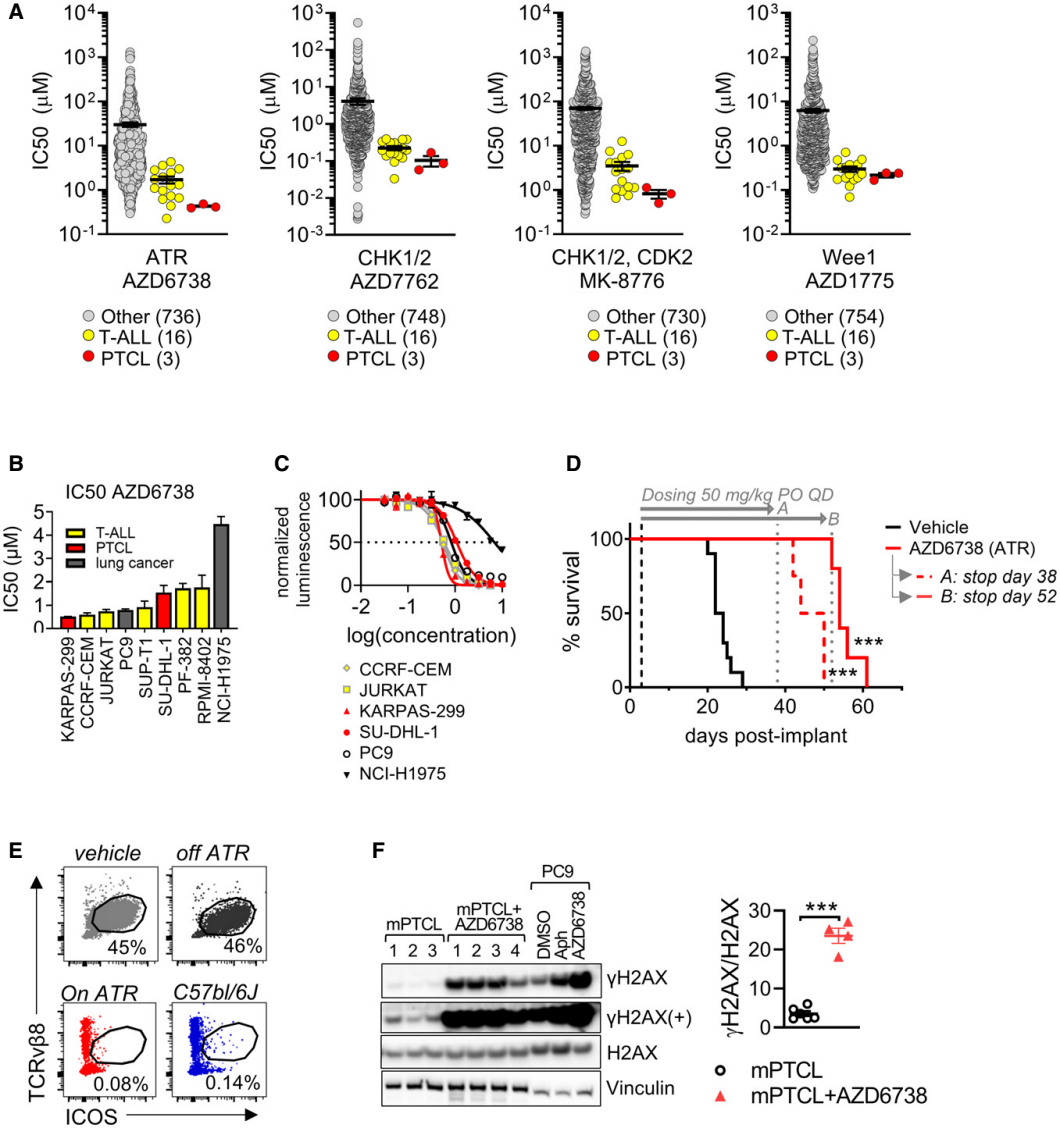
Sensitivity of PTCL to ATR pathway inhibition Sanger screen IC50 drug sensitivity data (IC50 values) for DDR inhibitors targeting ATR, CHK1/2, and Wee1 in T‐ALL, PTCL (DEL, KARPAS‐299, and SUP‐M2), and pan‐cancer cell lines. ANOVA *P* = 0.413 (AZD6738, ATR inhibitor), *P* = 0.750 (AZD772, CHK1/2 inhibitor), *P* = 0.119 (MK‐8776, ATR inhibitor), and 0.282 (AZD1775, Wee1 inhibitor). Bars represent SEM of biological replicates.PTCL (red) and T‐ALL (yellow) human cell lines were dosed *in vitro* with AZD6738 for 72 h and viable cell numbers assayed by luminescence readout using Cell TiterGlo. Average IC50 values from three experiments are plotted. Bars represent SEM.Representative dose–response curve of AZD6738. PC9 and NCI‐H197 lung cancer cell lines are DDR inhibitor sensitive and resistant, respectively, and included as drug sensitivity controls. Dotted line represents 50% normalized luminescence levels.Kaplan–Meyer curve of mice implanted IV with mPTCL splenocytes and dosed with ATR inhibitor ceralasertib (AZD6738) 50 mg/kg PO QD (*n* = 9) or vehicle (*n* = 10). On day 38, mice on AZD6738 therapy were randomized to discontinue therapy (*n* = 4, group A) or continue therapy for a further 14 days (*n* = 5, group B). The dosing period is indicated by gray arrows. Log‐rank test *P = *0.0004 (ATR group A, stop day 38) and *P* = 0.0001 (ATR, stop day 52) versus the vehicle control arm. Group A versus B: *P* = 0.0040. Median survival times: days 23 (vehicle), 47 (A), and 54 (B).Flow cytometry dot plots of TCRvβ8^mid^ ICOS^hi^ infiltrating TCRβ^+^ T‐cells from spleens. Mice were implanted IV with mPTCL splenocytes and administered intermittent AZD6738 (50 mg/kg PO QD) for 71 days post‐implant (see Appendix Fig S6D). Tumor burden representative of 6 out of 8 mice on prolonged ATR inhibition therapy (day 71 post‐implant) is compared with endpoint spleens from a vehicle‐treated mouse and an ATR‐treated mouse that relapsed off‐therapy. A wildtype C57bl/6J spleen is shown in blue. Percentages of total viability marker‐negative cells are gated.Western blot for γH2AX relative to total H2AX levels of spleen lysates from mice dosed for 71 days with AZD6738 (ATR; *n* = 4) relative to untreated mPTCL spleens taken at endpoint (*n* = 6). *T*‐test *P* = 2.70E‐06. PC9 cells dosed *in vitro* 24 h with 1 µM aphidicolin (Aph; an inhibitor of DNA replication) or with AZD6738 (ATR) are positive controls for DNA damage. Bars represent SEM. ***P < 0.001.

During the replication stress response, RPA proteins coat ssDNA causing the recruitment of ATR and associated proteins. ATR subsequently phosphorylates and activates CHK1, initiating a response that slows cell cycle progression and attempts to stabilize or repair replication forks (Forment & O'Connor, 2018). Among other functions, CDK1 inhibitor Wee1 contributes to these responses downstream of CHK1 activation (Forment & O'Connor, 2018). As consequence, inhibitors of ATR, CHK1, and Wee1 promote premature cell cycle entry and mitotic catastrophe (Forment & O'Connor, 2018). To test the potential of targeting the replication stress response in PTCL, we analyzed Sanger drug sensitivity screen data. Reports show that T‐cell acute lymphoblastic leukemia (T‐ALL) cell lines and xenografts are sensitive to inhibitors of ATR, CHK1, and Wee1 (Boudny & Trbusek, 2020). In our analysis, T‐ALL cell lines showed increased sensitivity to inhibitors of ATR (AZD6738, ceralasertib), CHK1/2 (AZD7762 and MK‐8776), and Wee1 (AZD1775, adavosertib) as measured by a reduced IC50 compared with other non‐T‐cell cancer cells (Fig 7A–C). Compared with T‐ALL, the three available PTCL cell lines representing ALCL were at least as sensitive to each DDR inhibitor (ANOVA *P* < 0.05 for AUCs of AZD6738, MK‐8776, and AZD1775, *P* ≥ 0.05 for IC50 values; Fig 7, Appendix Fig S6C). We confirmed comparable sensitivity of five T‐ALL and two ALCL PTCL cell lines to ATR and Wee1 *in vitro* (Fig 7B and C, Appendix Fig S6D). These data suggested a potential therapeutic vulnerability of precursor and mature T‐cell malignancies to DDR inhibitors.

Finally, to extend these data into an *in vivo* setting, we evaluated the anti‐tumor efficacy of the ATR inhibitor in the mPTCL model. In agreement with observations in cell lines, daily treatment with AZD6738 durably and significantly increased the survival of mice implanted with mPTCL (Fig 7D). Therapeutic benefit was observed regardless of whether AZD6738 treatment was initiated soon after implant (day 3) or later (day 14; Appendix Fig S6E). Characterization of mice undergoing prolonged ATR inhibition therapy indicated minimal tumor burden and a further increase in γH2AX induction, indicating greater DNA damage (Fig 7E and F). These data demonstrate that AZD6738 treatment suppressed lymphoma progression. In summary, the analysis of mPTCL uncovered a DDR vulnerability, providing the rationale for ATR inhibition therapy for human PTCL‐GATA3 treatment.

## Discussion

PTCL are aggressive cancers with an unmet need for effective therapies. Biologic heterogeneity and disease rarity hinder clinical trials, raising the demand for translationally relevant preclinical models. Here, we characterized a transplantable murine T‐cell lymphoma (mPTCL) resembling human PTCL‐GATA3 that spontaneously arose from a mouse bearing a GC B‐cell‐directed *Blimp1* deletion. Notably, the mPTCL was sensitive to ATR inhibition *in vivo*, and this was corroborated by the study of human PTCL cell lines. This work uncovered DDR vulnerability in PTCL and indicates that targeting this pathway, particularly through ATR inhibition, is a viable and novel therapeutic strategy for PTCL.

Like many human PTCL, the mPTCL was clonal, had CD4 single positive T‐cell origin, expressed Tfh markers, displayed bystander B‐cell activation, and a human‐relevant oncogenic mutation in β‐catenin (Vallois *et al*, 2016; Fiore *et al*, 2020). The mPTCL seemed to recapitulate the poor prognostic Th2‐like GATA3 subgroup of PTCL, owing to common GATA3 and Th2‐associated gene expression, MYC activation, and genome instability (Iqbal *et al*, 2010, 2014; Wang *et al*, 2014a; Manso *et al*, 2016; Amador *et al*, 2019; Watatani *et al*, 2019). PI3K/mTOR signatures (Iqbal *et al*, 2010, 2014; Manso *et al*, 2016; Heavican *et al*, 2019) were also enriched, but in the absence of pathway mutations or evidence of signaling activation. The mPTCL may therefore serve as an experimental tool to investigate PTCL biology and trial novel therapies. We are aware of one report of a murine PTCL‐GATA3‐like lymphoma in the literature, in which mature T‐cell lymphomas develop on a background of *VAV1‐*mutant transgenic (Tg)/*p53^nul^* mice (Fukumoto *et al*, 2020). *VAV1‐*mutant tumor cells, like the mPTCL and clinical PTCL (Heavican *et al*, 2019; Watatani *et al*, 2019), co‐expressed Th2 and Tfh markers (Fukumoto *et al*, 2020). Together, co‐expression of these markers raises a possible limitation of classifying PTCL into a discrete cell‐of‐origin.

A unique feature of the mPTCL in our study is that T‐cells were not directly transformed by an engineered genetic mutation. *ITK‐SYK^CD4‐Cre^*, *Rhoa* ^G17V^ *Tet2* ^nul^ mice, and *VAV1‐Tg/p53^nul^* mice developed from genomic or T‐cell‐targeted introduction of clinically relevant mutational events (Ellyard *et al*, 2012; Wartewig *et al*, 2017; Cortes *et al*, 2018; Ng *et al*, 2018; Fukumoto *et al*, 2020). Lymphomas from these mice each exhibit disease‐relevant cellular phenotypes and chronic T‐cell activation. Despite the strength of genetically‐induced tumor models, they may not necessarily represent early transformation events. Conversely, lymphomagenesis of the mPTCL occurred in the context of genetically modified and dysregulated bystander GC B‐cells (Casola *et al*, 2006), yet displayed biology and an oncogenic mutation consistent with clinical observations. While it is unclear what triggers PTCL lymphomagenesis and whether B‐cells are involved (Gru *et al*, 2015; Fiore *et al*, 2020), our observations of a Tfh‐like tumor phenotype and activation/transformation of B‐cells and T‐cells implicate a possible connection with the GC. The *Cγ1‐*Cre *Blimp1* ^fl/fl^ mouse models a scenario of blocked B‐cell differentiation and hyperplasia (Calado *et al*, 2010), a setting that might result in chronic co‐stimulation and activation of T‐cells within the GC that may be permissive to T‐cell transformation. The mechanisms governing a role of GC B‐cells in T‐cell lymphomagenesis is an area for future investigation.

The clonal occurrence of the known oncogenic mutation *Ctnnb1* K335T (Pilati *et al*, 2014; Rebouissou *et al*, 2016; Liu *et al*, 2020) in the mPTCL was of significance for the gene’s relevance to normal T‐cell functions and to human PTCL. In untransformed T‐cells, β‐catenin is expressed and stabilized downstream of TCR signaling (Lovatt & Bijlmakers, 2010) and directly regulates GATA3 expression during Th2‐cell differentiation (Yu *et al*, 2009; Notani *et al*, 2010). While aberrant β‐catenin in thymic T‐cells is sufficient for leukemia initiation (Kaveri *et al*, 2013; Dose *et al*, 2014; Gekas *et al*, 2016), a role for oncogenic β‐catenin in mature T‐cell malignancies is not well‐defined. However, whole‐exome and targeted sequencing studies identified stabilizing *CTNNB1* mutations in 6% of PTCL (in a series of 85 cases) and mutations in Wnt/β‐catenin negative‐regulator genes (Schatz *et al*, 2015; Laginestra *et al*, 2020). Further reports of Wnt/β‐catenin activation in human PTCL cases (Groen *et al*, 2008; Iqbal *et al*, 2014), suggest that β‐catenin may be oncogenic in a subset of T‐cell lymphomas. However, *CTNNB1* mutations were found to co‐occur with *RHOA* or *TET2* mutations in human PTCL cases (Vallois *et al*, 2016). It is therefore unlikely that *CTNNB1* is sufficient as a tumor‐driver in PTCL, and other genetic or epigenetic alterations are likely involved in lymphomagenesis.

Anti‐tumor activity of PI3K and mTOR inhibitors has been shown in human PTCL cell lines and mouse models with constitutive PI3K/AKT pathway activation (Cortes *et al*, 2018; Horwitz *et al*, 2018; Ng *et al*, 2018). However, this biology did not appear relevant to the mPTCL, which was resistant to AKT pathway‐targeting agents. Rather, our work proposes capitalizing on high levels of replication stress by therapeutically targeting the DDR (Kotsantis *et al*, 2018). We thus implicate ATR inhibitor sensitivity in the setting of possible MYC oncogene‐induced replication stress in PTCL. These data are consistent with reports of ATR‐CHK1 pathway dependency or therapeutic vulnerability of a range of MYC‐overexpressing tumors (Hoglund *et al*, 2011; Murga *et al*, 2011; Ferrao *et al*, 2012; Kruger *et al*, 2018; Young *et al*, 2019).

The extent to which high replication stress characterizes human PTCL is unknown, though our preliminary analysis of ALCL cell lines (a form of PTCL) suggests a degree of sensitivity of these cancers to ATR/CHK1/Wee1 inhibitors. There is a possibility of application of DDR inhibitors across additional PTCL subgroups, since the features of MYC activity, genomic instability, and loss of TP53/CDKN2A/B, found in PTCL‐GATA3, are linked with increased replication stress and DDR activation in other cancers (Kwok *et al*, 2016; Gadhikar *et al*, 2018; Young *et al*, 2019). Further PTCL‐relevant genotypes such as ATM‐deficiency (Schatz *et al*, 2015; Heavican *et al*, 2019; Watatani *et al*, 2019; Laginestra *et al*, 2020) are also associated with replication stress and DDR inhibitor sensitivity (Kwok *et al*, 2016; Min *et al*, 2017; Young *et al*, 2019; Yap *et al*, 2021). Normal antigen‐activated T‐cells display extremely high proliferation rates, signs of DNA damage, such as γH2AX induction, and a DDR (McNally *et al*, 2017). Immature T‐cell leukemia (T‐ALL) cell lines were highly sensitive to a single agent and combination treatment with DDR inhibitors WEE1, CHK1, and ATR (Ghelli Luserna Iacobucci *et al*, 2015; Di Rorà *et al*, 2019). A CHK1 inhibitor (PF‐0477736) significantly prolonged the survival of mice implanted with a mutagen‐induced murine lymphoid T‐cell leukemia (Iacobucci *et al*, 2015). These data suggest a potential inherent sensitivity of proliferating T‐cells to DDR inhibitors, which might also be a vulnerability of PTCL.

The ATR inhibitor ceralasertib (AZD6738) was shown to be well‐tolerated in patients with advanced tumors (Dillon *et al*, 2019) and was actively undergoing phase I/II clinical trials as monotherapy and in combination with other agents (e.g., NCT03328273, NCT04564027, and NCT03770429). The prolonged control of mPTCL tumor progression *in vivo* supports the use of ATR inhibition as a single agent for the treatment of the GATA3 subgroup of PTCL. Notably, we observed toxicity with continuous dosing of AZD6738 in mice. In the clinic, various ATR inhibitors have led to dose‐dependent and reversible hematologic adverse events that were managed by treatment breaks and/or intermittent dose schedules (Yap *et al*, 2020, 2021). Therefore, alternate dosing schedules should be considered for PTCL. Given the urgency for effective PTCL treatments, ATR inhibition therapy may represent an opportunity for immediate application to PTCL‐GATA3 patients. We do not exclude that a broader range of PTCL might be eligible for a DDR treatment strategy, but this requires further investigation using appropriate models of other PTCL subtypes.

In conclusion, through the characterization of a novel transplantable murine lymphoma that resembled clinical PTCL‐GATA3, we provide the first evidence for ATR inhibition as a candidate therapy for PTCL.

## Materials and Methods

### *In vivo* studies

*In vivo* studies were performed in the United Kingdom and Home Office approved them in accordance with the Animal Scientific Procedures Act 1986 (ASPA), IACUC guidelines, and AstraZeneca Global Bioethics policy. Studies were conducted on project licenses PCE886633 and P0EC1FFDF and were approved by the local AWERB committee. Animals were housed in compliance with Home Office guidelines. A novel murine lymphoma was isolated from a *Cγ1*‐Cre *Blimp1* ^fl/fl^ mouse (Mouse ID #2695) that developed splenomegaly (Calado *et al*, 2010). Total splenocytes in single‐cell suspension were expanded *in vivo* by intravenous injection into a single naïve C57bl/6N mouse (passage (P) 1), which itself subsequently developed a disseminated lymphoma (Fig 1, Appendix Fig S1). P1 splenocytes were serially passaged through female C57BL/6J mice aged 8–12 weeks (Jackson Laboratories), generating four parallel and traceable lineages of lymphomas by the fourth passage (termed “a”, “b”, “c” and “d”). These lineages were phenotypically stable for up to seven passages.

Lineage “a” splenocytes were passaged once through CD45.1 congenic mice (*Ptprc^a^* allele; in‐house colonies) and stocks of pooled cells were prepared from these neoplastic spleens. Lineage “a” cells were implanted for efficacy and RNA sequencing studies at 10^4^ cells/mouse. Murine lymphoma cells can be made available for non‐commercial purposes under an MTA with The Francis Crick Institute.

Tumor progression and animal welfare were monitored using a scoring system that included gentle palpation of the abdomen to estimate the degree of liver enlargement. Welfare endpoint was defined as liver enlargement reaching 12 mm in diameter or presentation of abnormal respiration and/or other clinical signs. Tissues for lymphoma characterization were obtained at the time of or preceding welfare endpoint. Additional tumor‐free (wildtype) C57bl/6J mice were included as control tissues.

PI‐3065, a small molecule inhibitor with selectivity for PI3K p110δ (Ali *et al*, 2014), was administered at 75 mg/kg PO QD, formulated in 0.5% HPMC, 0.1% Tween 80. Capivasertib (AZD5363), a pan‐AKT isoform inhibitor (Davies *et al*, 2012) was administered at 130 mg/kg PO BID 4 days on, 3 days off for 4 weeks, and formulated in 10% DMSO, 25% kleptose pH 5.0. Ceralasertib (AZD6738), an inhibitor of ATR (Vendetti *et al*, 2015), was administered at 50 mg/kg PO QD and formulated in 10% DMSO, 40% propylene glycol. Control groups received vehicle treatment. Dosing was initiated 3 or 7 days post‐inoculation of cells (as indicated). Therapeutic efficacy was measured by time to reach the welfare endpoint (“survival”). Mice were randomized to groups using a random number generator. The experimenter was not blinded to the treatment group.

### Flow cytometry

Freshly collected tissues were kept on ice, passed through a 40 µm filter then cells stained with a viability marker (dilution 1:1,000 in PBS; Live/Dead Fixable Blue, ThermoFisher Scientific). Cells were washed once in FACS buffer (0.5% BSA in PBS) then stained for surface antigens in FACS buffer for 30 min in the dark. Washed cells were fixed and permeabilized using Cytofix (BD Biosciences) for surface stains, or the Transcription Factor Staining kit (eBioscience) for intracellular stains. TCR Vβ repertoire was assessed using the anti‐mouse TCR Vβ Screening Panel (BD Biosciences). Data were acquired on a BD Fortessa and analyzed using FlowJo software (V.10, Treestar). Primary antibodies and their dilutions are listed under “Antibodies.”

### TCR sequencing

One normal and one lymphoma‐infiltrated spleen were analyzed using ArcherDX mouse beta version Immunoverse™‐HS TCR alpha/delta/beta/gamma Kit and sequenced on Novaseq600 (PE150). TCR sequences were first error corrected and deduplicated using the Archer analysis pipeline with default parameters on Amazon Web Services (ArcherDX, Boulder CO). This was followed by alignment of the sequence reads to reference V, (D), J, and C regions of TCRs, assembly of clonotypes and extraction of CDR3 regions, and the export of data by chain per sample using the MiXCR algorithm. The reported clonotype count is the total number of unique CDR3 sequences present in the sample.

### RNA sequencing and analysis

Whole tumor‐free or lymphoma‐infiltrated C57bl/6J spleens were collected from mice 21 days post‐implant. One‐half of each spleen was flash‐frozen and lysed using Qiazol. From the other half spleen, up to 400,000 viability dye‐negative CD45^+^TCRβ^+^CD8^−^TCRβ8^+^ (lymphoma) or CD45^+^TCRβ^+^CD8^−^CD4^+^ (wildtype) CD4^+^ T‐cells were purified using an Aria sorter (BD Biosciences). RNA was extracted from spleens using a miRNAeasy kit and from sorted cells using a RNeasy kit with an adapted MinElute protocol (Qiagen). The resulting RNA was quality validated using a Nanodrop and Bioanalyser (Agilent). Libraries were prepared using Illumina TruSeq mRNA Stranded kit (spleens) and Takara SMART Seq V4 + Nextera XT ultra‐low input protocol (sorted cells) and sequenced on Illumina NovaSeq6000 PE50.

The python toolkit bcbio 1.1.6 (https://github.com/bcbio/bcbio‐nextgen) was used for quality control and for gene expression quantification. Reads were aligned to the Genome Reference Consortium genome build GRCm38 augmented with transcript information from Ensembl release using his at 2 2.1.0 (https://www.ncbi.nlm.nih.gov/pmc/articles/PMC5130069/#ref‐8). Alignments were evaluated for evenness of coverage, rRNA content, genomic context of alignments, and complexity using a combination of FastQC, Qualimap, and custom tools. Transcripts per million (tpm) measurements were made against the mouse mm10 Ensembl transcriptome using Salmon 0.14.1 without alignment or adapter trimming. The R package tximport generated a gene by sample count table. DESeq2 R package (version 1.22.1) was used to normalize for library size and perform differential expression analysis.

Single sample Gene Set Variance Analysis (GSVA), which compares pathway or gene set expression across populations (Hänzelmann *et al*, 2013) was analyzed on spleen samples. Normalized counts were uploaded into GSEA (v2.2.4) for gene enrichment analysis. Signatures were accessed from MSigDB (v7.2) and published papers. Differential gene expression was analyzed using ROSALIND^®^ (https://rosalind.bio/). The limma R library was used to calculate fold changes and *P*‐values and perform optional covariate correction. Genes of fold change +/−2 and adjusted *P*‐value < 0.05 were analyzed by Ingenuity Pathway Analysis (IPA; Ingenuity Systems, Redwood City, CA). Differential gene expression, gene set enrichment, and upstream regulator pathway analysis results are presented in the Datasets [Link], [Link], [Link], [Link], [Link], [Link].

### Whole exome sequencing and analysis

2 × 10^6^ T‐cells from four lymphoma‐infiltrated spleens were isolated by EasySep (StemCell Technologies). Exon libraries were prepared using Agilent SureSelectXT Mouse Exon kit and sequenced on Illumina’s NextSeq300.

Quantification of the data was done using bcbio 1.1.4 (https://github.com/bcbio/bcbio‐nextgen). BWA version 0.7.17 (https://www.ncbi.nlm.nih.gov/pmc/articles/PMC2705234/) was used within BCBIO to align the data against the mouse mm10 assembly. Agilent mouse exome mm9 genome panel bedfile was changed to mm10 using liftover tool, which was then used for variant and copy number calling. Vardict 1.5.8 (https://academic.oup.com/nar/article‐abstract/44/11/e108/2468301) was run within Bcbio on the aligned bams to detect SNVs and indels. Copy number calls were generated using seq2c and cnvkit 0.9.6a0 and structural variants using manta 1.5.0 to analyze the sequencing data.

A matched germline *Cγ1*‐Cre *Blimp1* ^fl/fl^ DNA sample was unavailable therefore extensive filtering steps were necessary to remove all possible likely germline variants and thus identify somatic mutations acquired during T‐cell transformation in the *Cγ1*‐Cre *Blimp1* ^fl/fl^ mouse. The sequencing genome datasets of 129P2/OlaHsd, 129S1/SvImJ, 129S5SvEvBrd, C57Bl/6NJ, and Balb/c mice were accessed (https://www.sanger.ac.uk/data/mouse‐genomes‐project/) due to the possible involvement of these (or closely related) strains in the generation of the *Cγ1*‐Cre *Blimp1* ^fl/fl^ line. Any matching variants with mPTCL were removed. Next, calls of variant depth ≥ 4 shared by all four mPTCL but not found in wildtype C57bl/6J underwent variant effect prediction using Ensembl SIFT (Sorting Intolerant From Tolerant; https://www.ensembl.org/Tools/VEP). Deleterious variants (SIFT score ≤ 0.05) were further filtered to remove dbSNPs or matches in the SNP viewer (i.e., additional likely mouse substrain‐associated germline variants; https://www.sanger.ac.uk/sanger/Mouse_SnpViewer/rel‐1505). This resulted in a list of 44 distinct mutations deemed “somatic.” Those listed in OncoKB (https://www.oncokb.org/) (accessed June 2019) were prioritized. The distribution of allele frequencies of dbSNPs (before variant filtration steps) on chromosomes 10 (location of *Blimp1* locus) and 6 (*Cγ1* locus) was used to establish cutoffs for clonality and heterozygosity. CTNNB1 K335T protein structure was accessed from COSMIC‐3D. *CTNB1* protein alignment was performed at Uniprot.org.

### Histology

Formalin‐fixed tissue sections were stained using Vision Biosystems BondMax II and antibodies: CD4 (EPR19514), CD21 (EP3093), c‐MYC (Y69), CD3 (EPR4517), Ki67 (SP6), γH2AX (HRP conjugated, EP854(2)Y; all Abcam), β‐catenin (C14, BD Biosciences), BCL6 (C‐19, SantaCruz), and B220/CD45R (RA3‐6B2, BD) and counterstained with hematoxylin. Slides were scanned using the Aperio AT2 system, visualized, and quantified using QuPath (0.2.2).

### PCR genotyping

DNA was extracted from the lymphoma‐infiltrated spleen and analyzed for the presence of *Blimp1* ^fl/fl^ *Cγ1*‐Cre and YFP alleles. Primer sequences are listed below. Wildtype, total T‐cells, total B‐cells, or CD45.2^+^ positively selected cells were evaluated. Cells were isolated using kits and a biotinylated anti‐CD45.2 (clone 104) antibody from Stem Cell Technologies. Results from mPTCL and mBCL samples were independently validated by Transnetyx using a customized qPCR assay.

#### *Cγ1*‐Cre primers

Wildtype/forward: TGT TGG GAC AAA CGA GCA ATC, Common/reverse: GTC ATG GCA ATG CCA AGG TCG CTA G, Mutant/forward: GGT GGC TGG ACC AAT GTA AAT A.

#### *Blimp1* ^fl/fl^ primers

##### Common/forward

GCC CAG TGA CTC AAA GCA CTA, Mutant(floxed)/reverse: TAT GGT CTT CTC ATG TTG GGG, Mutant(excised)/reverse: GGT GTC TGA AGA GCA AAG CTG.

##### Generic Rosa26Sor (GFP) primers

Wildtype/forward: CTG GCT TCT GAG GAC CG, Wildtype/reverse: CAG GAC AAC GCC CAC ACA, Mutant/forward: AGG GCG AGG AGC TGT TCA, Mutant/reverse: TGA AGT CGA TGC CCT TCA G.

### Human PTCL analysis

Affymetrix datasets GSE19069 (Iqbal *et al*, 2010) and GSE58445 (Iqbal *et al*, 2014) of previously classified PTCL (Heavican *et al*, 2019) were analyzed for expression of Tfh marker genes using ClustVis (https://biit.cs.ut.ee/clustvis/). Principal component analysis of the top 200 genes lacked clustering by dataset therefore both datasets were included for analysis. The most specific probeset was selected if multiple probesets mapped to a gene. The proportion of GATA3 and BCL6 positivity by IHC was calculated from data of 86 PTCL‐NOS cases from which data on both stains was available (Watatani *et al*, 2019).

### Western blotting

Snap‐frozen tissues were mechanically homogenized and lysed in KDR lysis buffer containing protease inhibitor (Roche) and phosphatase inhibitor cocktails I and II (Sigma). Primary antibodies and their dilutions are listed under “Antibodies.” Band intensity was analyzed by ImageJ. Control protein lysates were obtained from A20 murine DLBCL cell line and human PC9 cells dosed 24 h *in vitro* with DMSO, 1 µM aphidicolin (a DNA replication inhibitor), or 1 µM AZD6738.

### Antibodies

**Table:** 

Application	Protein	Conjugate	Working dilution	Supplier	Catalog no.
Flow Cytometry	B220/CD45R	BV711	1:800	BioLegend	103255
Flow Cytometry	BCL6	PE	1:100	BioLegend	648303
Flow Cytometry	CD138	BV605	1:200	BioLegend	142515
Flow Cytometry	CD19	BUV737	1:800	BD Biosciences	564296
Flow Cytometry	CD3	BUV396	1:200	BD Biosciences	563565
Flow Cytometry	CD38	BV421	1:400	BioLegend	102732
Flow Cytometry	CD4	BV711	1:800	BioLegend	100557
Flow Cytometry	CD40L/CD154	PE‐Cy7	1:100	BioLegend	106511
Flow Cytometry	CD44	BUV737	1:1,000	BD Biosciences	564392
Flow Cytometry	CD45	BV786	1:1,000	BD Biosciences	564225
Flow Cytometry	CD45.1	BV605	1:400	BioLegend	110737
Flow Cytometry	CD45.2	Alexa488	1:400	BioLegend	109816
Flow Cytometry	CD62L	PE‐CF594	1:800	BD Biosciences	562404
Flow Cytometry	CD8	BV650	1:800	BioLegend	100742
Flow Cytometry	CD86	BUV395	1:200	BD Biosciences	564199
Flow Cytometry	CD95/Fas	PE‐CF594	1:1,000	BD Biosciences	562499
Flow Cytometry	CXCR5	buv395	1:200	BD Biosciences	563980
Flow Cytometry	CD16/CD32 (Fc block)		1:200	ThermoFisher	14‐0161‐86
Flow Cytometry	GATA3	PE	1:20	BioLegend	653804
Flow Cytometry	ICOS	PE‐Dazzle	1:800	BioLegend	313531
Flow Cytometry	IgD	BV650	1:1,000	BioLegend	405721
Flow Cytometry	IgM	PE/Cy7	1:1,200	ThermoFisher	25‐5790‐82
Flow Cytometry	IL7Ra	PE	1:200	BioLegend	135010
Flow Cytometry	Ki67	Alexa488	1:1,000	BioLegend	151204
Flow Cytometry	Ki67	Alexa647	1:1,000	BioLegend	652407
Flow Cytometry	MHC‐II	Af700	1:1,200	BioLegend	107622
Flow Cytometry	Mouse IgG1, κ Isotype Ctrl	PE	1:200	BioLegend	400112
Flow Cytometry	Mouse IgG2b, κ Isotype Ctrl	PE	1:1,600	BioLegend	400311
Flow Cytometry	PAX5	PE	1:400	BioLegend	649707
Flow Cytometry	PD1	BV421	1:150	BioLegend	135218
Flow Cytometry	TCR Vβ8.1, 8.2	PE	1:200	BioLegend	140104
Flow Cytometry	TCRb	A647	1:400	BioLegend	109217
Flow Cytometry	Thy1/CD90.2	BUV786	1:400	BioLegend	105331
Western blotting	AKT		1:4,000	Cell Signaling Technologies	2972
Western blotting	CHK1		1:1,000	Cell Signaling Technologies	2360
Western blotting	c‐MYC		1:500	Cell Signaling Technologies	5605
Western blotting	GAPDH		1:2,000	Cell Signaling Technologies	2118
Western blotting	Goat anti‐mouse	HRP	1:2,000	Cell Signaling Technologies	7076
Western blotting	Goat anti‐rabbit	HRP	1:2,000	Cell Signaling Technologies	7074
Western blotting	GSK3β		1:1,000	Cell Signaling Technologies	9315
Western blotting	H2AX		1:2,000	Cell Signaling Technologies	2595
Western blotting	pAKT (Ser473)		1:1,000	Cell Signaling Technologies	4060
Western blotting	pGSK3β (Ser9)		1:1,000	Cell Signaling Technologies	9323
Western blotting	pS6 (Ser235/236)		1:1,000	Cell Signaling Technologies	4858
Western blotting	pZAP70 (Y319)		1:500	Cell Signaling Technologies	2717
Western blotting	RPA32		1:1,000	Cell Signaling Technologies	52448
Western blotting	S6		1:5,000	Cell Signaling Technologies	2217
Western blotting	Vinculin		1:20,000	Cell Signaling Technologies	13901
Western blotting	ZAP70		1:500	Cell Signaling Technologies	3165
Western blotting	γH2AX (Ser139)		1:1,000	Cell Signaling Technologies	2577

### Pan‐human cancer cell line analysis

Gene and protein expression analyses of human T‐ALL cell lines were downloaded from the Cancer Cell Line Encyclopedia (Ghandi *et al*, 2019) (https://portals.broadinstitute.org/ccle). Expression data were RMA Normalized, log2 converted, and centered around the mean. Drug sensitivity data were downloaded in June 2020 from the Genomics of Drug Sensitivity in Cancer database (http://www.cancerrxgene.org/). Cell lines were annotated by cancer type according to CCLE.

### ELISA

The Mouse IgG ELISA Kit (ab151276, Abcam) was used according to the manufacturer’s recommendation for the detection of IgG in mouse serum samples.

### Cell cultures

Lymphoma cells were freshly isolated and cultured in RPMI 1640 media (with Glutamax, ThermoFisher), 10% fetal calf serum (FCS; Gibco), 50 µM 2‐mercaptoethanol (Gibco), sodium pyruvate (1 mM, Sigma), HEPES (10 mM, Sigma), Penn/Strep (Gibco) and non‐essential amino acids (Gibco). Cells were purified using the EasySep CD4 T‐cell‐negative selection kit (Stem Cell Technologies) and 50,000 viable cells were seeded per well of a 96‐well plate (Costar). Media were supplemented with recombinant mouse IL‐21 (BioLegend), IL‐7 (R&D Systems), IL‐4 (Peprotech), or IL‐2 (Peprotech). TCR stimulation was performed by pre‐coating overnight 96‐well culture plates (Costar) with anti‐mouse CD3 antibody (10 µg/ml, BD Biosciences) diluted in PBS (Gibco), washing plates the next day in PBS and adding anti‐mouse CD28 antibody (2 µg/ml, eBioscience/ThermoFisher) to media. Cells were incubated for 72 h before assaying by flow cytometry. Cells were cultured at 37°C in a humidified incubator (5% CO_2_).

Cell lines were authenticated by STR profiling and tested for mycoplasma contamination prior to use. PTCL, T‐ALL, and lung cancer cell lines were seeded into black‐walled 96‐well plates (Costar) in RPMI 1640 + Glutamax with 10% FCS and penn/strep antibiotics. Cells were dosed 24 h later with a dose range of DDR inhibitors. DMSO was equalized across wells. Cell viability/proliferation was assayed 72 h later by Cell TiterGlo assay (Promega). KARPAS‐299 (PTCL), SU‐DHL‐1 (PTCL), JURKAT (T‐ALL), PF‐382 (T‐ALL), and RPMI‐8402 (T‐ALL) were obtained from DSMZ. PC9 (lung adenocarcinoma) was obtained from ECACC. SUP‐T1 (T‐ALL) and NCI‐H1975 (NSCLC) were obtained from ATCC. CCRF‐CEM (T‐ALL) was purchased from JCRB. A20 murine B‐cell lymphoma cell line was obtained from ATCC and cultured in RPMI 1640 + Glutamax with 10% FCS, antibiotics with 50 µM β‐mercaptoethanol.

### Statistics

GraphPad Prism (v8) was used for statistical analysis. Significant differences between groups were analyzed by Student’s *t*‐test (two‐tailed, unpaired), or an ANOVA, and corrected for multiple comparisons where indicated using either the Holm–Sidak method or Tukey’s test. Statistical methods were not used to determine the sample size. Survival data were analyzed by log‐rank test. No samples or animals were excluded from analysis except in immunohistochemical analysis where no tumor cells could be detected in sections. Statistical significance is indicated as follows: *P < 0.05, **P < 0.01, ***P < 0.001, with *P* < 0.05 considered statistically significant. Error bars represent SEM unless otherwise indicated.

## Author contributions

**Elizabeth Kuczynski:** Conceptualization; Data curation; Formal analysis; Validation; Investigation; Visualization; Methodology; Writing—original draft; Writing—review & editing. **Giulia Morlino:** Resources; Investigation; Methodology. **Alison Peter:** Investigation; Methodology. **Anna M L Coenen‐Stass:** Software; Visualization; Methodology. **Jennifer I Moss:** Methodology; Project administration. **Neha Wali:** Investigation; Methodology. **Oona Delpuech:** Methodology; Project administration. **Avinash Reddy:** Data curation; Formal analysis; Visualization. **Anisha Solanki:** Investigation. **Charles Sinclair:** Conceptualization; Resources; Supervision; Funding acquisition; Methodology; Project administration; Writing—review & editing. **Dinis Calado:** Conceptualization; Resources; Supervision; Funding acquisition; Methodology; Project administration; Writing—review & editing. **Larissa S Carnevalli:** Conceptualization; Resources; Supervision; Funding acquisition; Methodology; Project administration; Writing—review & editing.

In addition to the CRediT author contributions listed above, the contributions in detail are:

EAK wrote the paper and performed experiments. CS, DPC, and LSC edited the manuscript and resourced experiments. EAK, AMLC‐S, NW, AS, CS, DPC, and LSC provided scientific input. EAK, AMLC‐S, and AR analyzed the genomic and gene expression data. AP, JIM, OD, NW, and AS provided technical support. DPC and GM provided the lymphoma samples. CS, DPC, and LSC funding acquisition and supervision.

## Disclosure and competing interests statement

EAK, AP, JIM, AR, AS, and LSC are current employees and shareholders at AstraZeneca. CS is a shareholder of AstraZeneca.

## Supporting information



Appendix

Dataset EV1

Dataset EV2

Dataset EV3

Dataset EV4

Dataset EV5

Dataset EV6

Dataset EV7

## Data Availability

The exome and RNA sequencing data have been deposited in the European Nucleotide Archive (ENA) at EMBL‐EBI under accession numbers PRJEB51833, PRJEB51834, and PRJEB51835. Source data are available in EV1‐7 and upon request. https://www.ebi.ac.uk/ena/browser/view/PRJEB51833
https://www.ebi.ac.uk/ena/browser/view/PRJEB51834
https://www.ebi.ac.uk/ena/browser/view/PRJEB51835
