# Supplementary material for: A preclinical model of peripheral T‐cell lymphoma GATA3 reveals DNA damage response pathway vulnerability
Source: EMBO Mol Med. 2022 May 5;14(6):e15816. doi: 10.15252/emmm.202215816 (PMC9174882; doi:10.15252/emmm.202215816)
Supplement: Supplementary file 1 — Appendix [file EMMM-14-e15816-s003.pdf]

Appendix: A preclinical model of peripheral T-cell lymphoma GATA3 reveals DNA damage response pathway vulnerability

Elizabeth A. Kuczynski et al.

Table of Content

Appendix Figure S1. Dendrogram of the passaging history of *Blimp1*$^{fl/fl}$ *Cγ1*-Cre-derived transplantable lymphomas....................3

Appendix Figure S2. Histologic and immunophenotypic analysis of lymphoma-bearing tissues. 5

Appendix Figure S3. The mPTCL has a Tfh-like phenotype and associates with activated B-cells and a coincident B-cell lymphoma. 7

Appendix Figure S4. Resemblance of mPTCL to normal and neoplastic T-cells.....9

Appendix Figure S5. Detection of an oncogenic mutation in mPTCL. 11

Appendix Figure S6. Targeting the DNA damage response in T-cell malignancies. 12

Appendix Figure S1

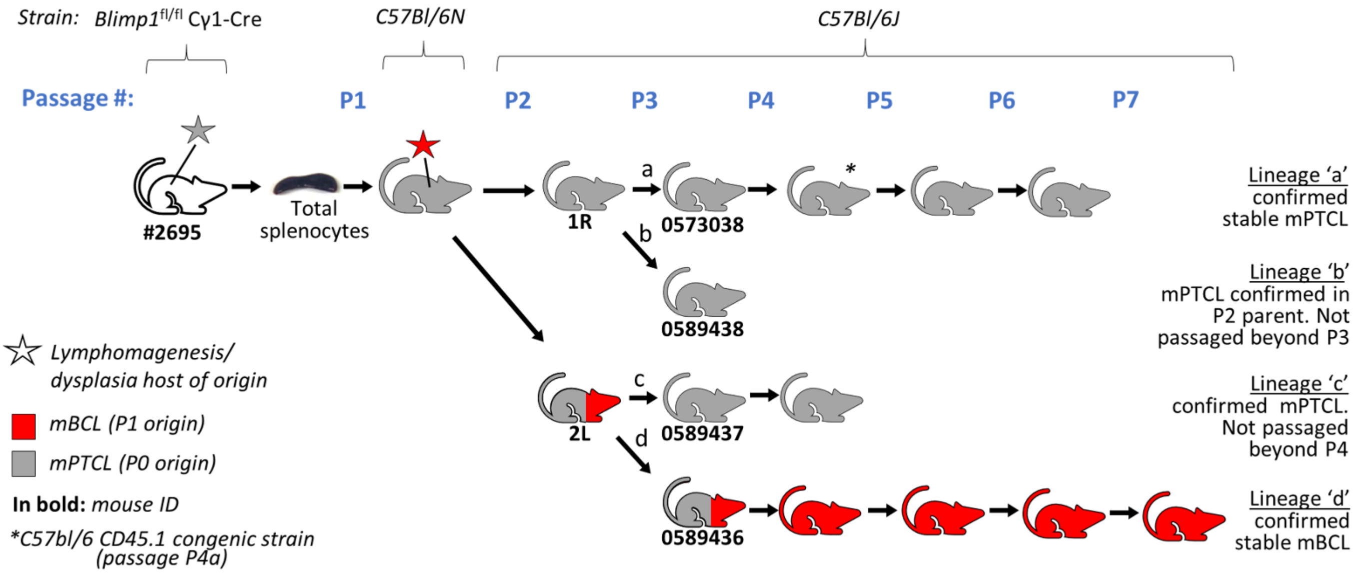

Appendix Figure S1. Dendrogram of the passaging history of *Blimp1*$^{fl/fl}$ *Cγ1*-Cre-derived transplantable lymphomas.

Total malignant splenocytes from a *Blimp1*$^{fl/fl}$ *Cγ1*-Cre-mouse with splenomegaly was passaged through wildtype C57bl/6 mice to generate a transplantable lymphoma model. Passage numbers, recipient mouse strains and the resulting lymphoma type found at each passage is indicated by color (T-cell lymphoma: mPTCL = grey, B-cell lymphoma: mBCL = red). Stars depict the host mouse and passage from which the T-cell and B-cell lymphomas originated.

Appendix Figure S2

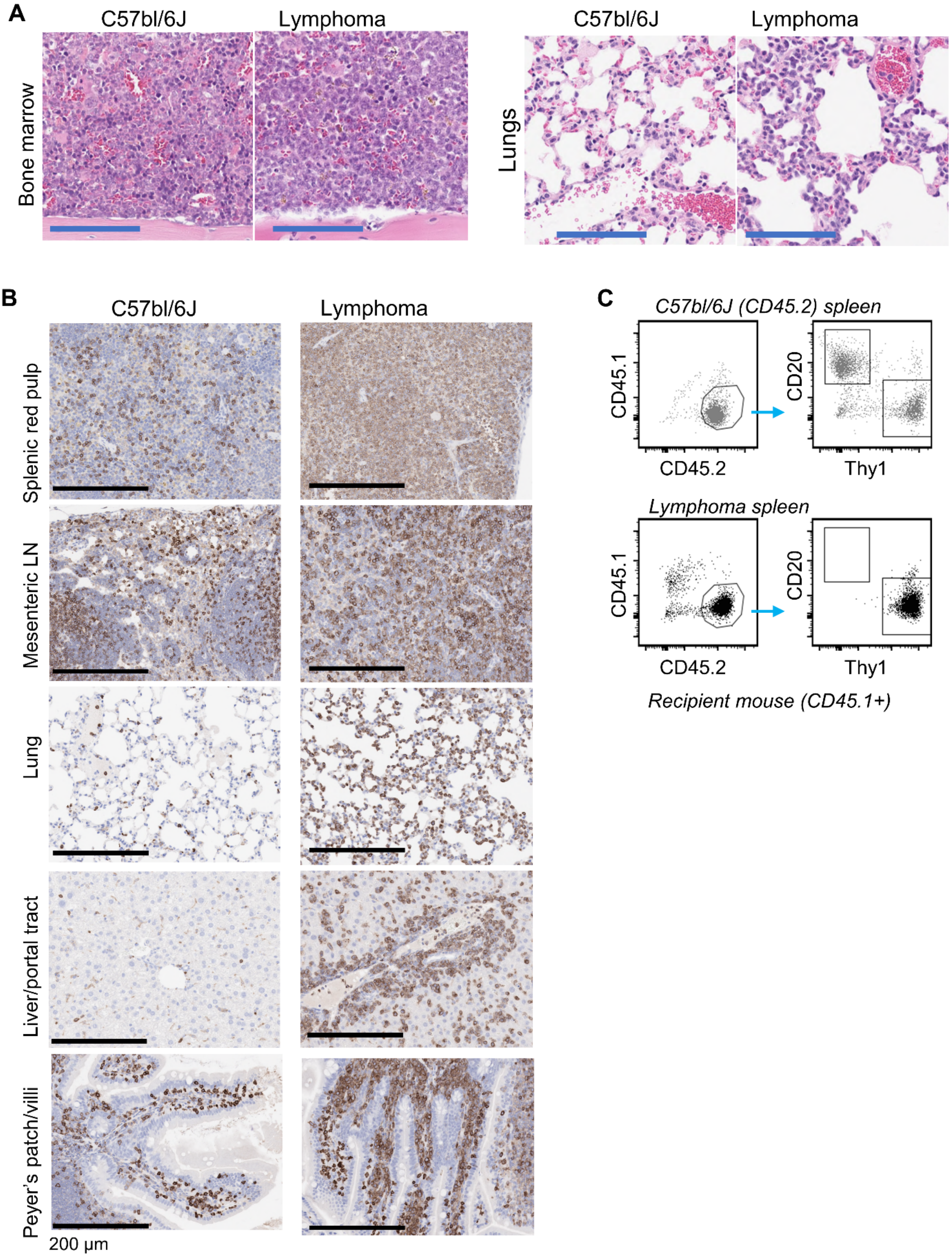

Appendix Figure S2. Histologic and immunophenotypic analysis of lymphoma-bearing tissues.

A. Lymphoma-bearing and wildtype C57bl/6J mouse femur (showing bone marrow) and lung stained by H&E. Images are representative of n=3 mice. **B.** Immunohistochemistry for CD3 in wildtype C57bl/6J and lymphoma-infiltrated mouse tissues. Images are representative of n=3 and n=6 mice, respectively. **C.** $5x10^5$ P3 splenocytes were implanted IV into congenic CD45.1 mice and resultant lymphomas were evaluated for expression of B and T-cell lineage markers. Expression levels of Thy1 and CD20 on CD45.2+ splenocytes are shown. CD45.2+ lymphoma cells were gated and compared to C57bl6/J CD4+ T-cells (n=4).

Appendix Figure S3

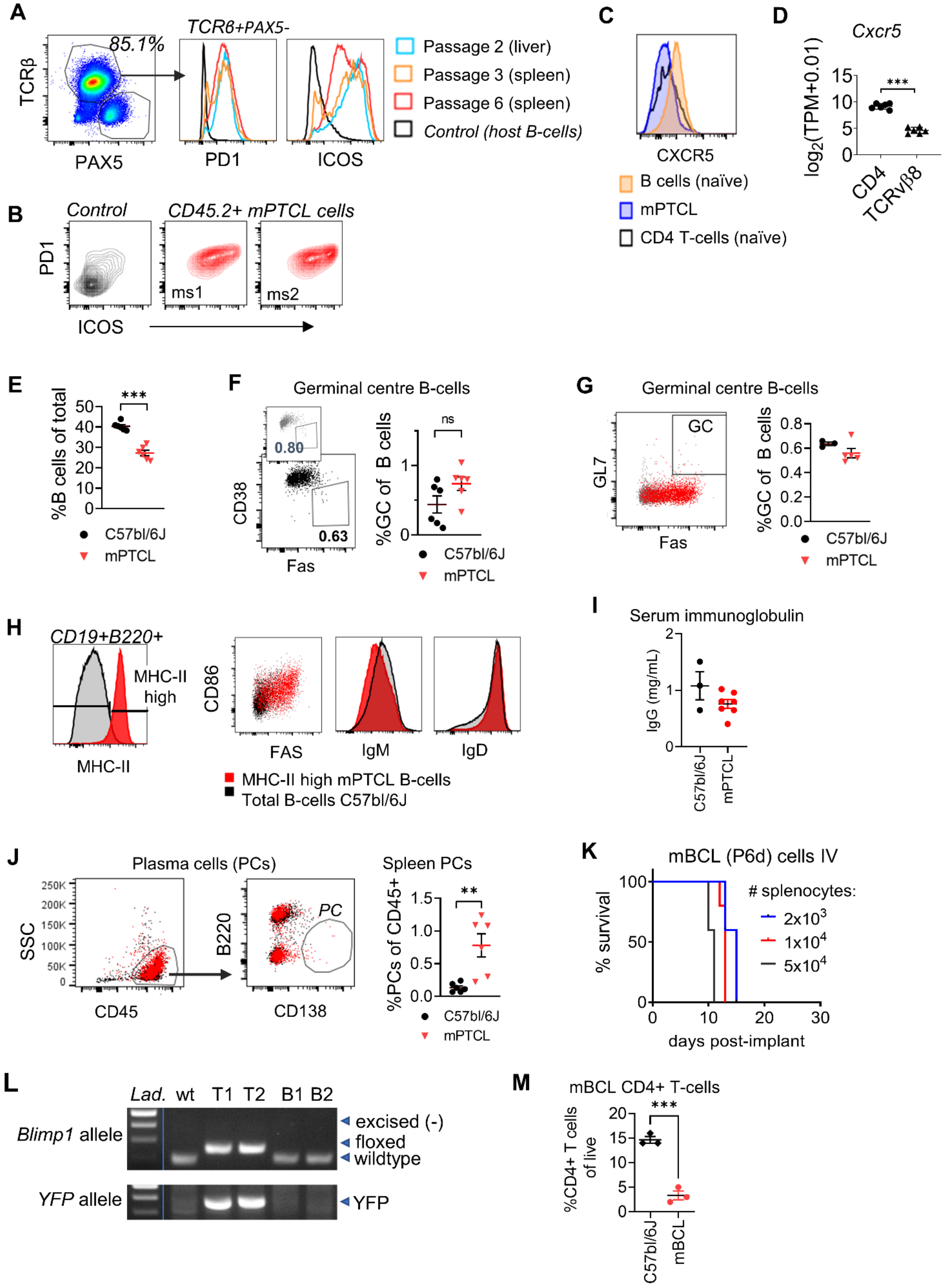

Appendix Figure S3. The mPTCL has a Tfh-like phenotype and associates with activated B-cells and a coincident B-cell lymphoma.

A. Fluorescence intensity of PD1 and ICOS expression on T-cells in lymphoma-infiltrated tissues of passages 2, 3 and 6. Host mPTCL B-cells serve as a negative control for staining. **B.** Expression of PD1 and ICOS on CD45.2+ lymphoma cells expanded in CD45.1 congenic hosts. Lymphomas from two representative (of 4) mice. Results are compared to naïve C57bl6/J T-cell controls. **C.** CXCR5 stain fluorescence intensity on passage 3 mPTCL cells (Thy1+ CD8- ICOS+). Data is representative of 3 mice. Naïve CD4+ T-cells and B-cells serve as negative and positive controls, respectively. **D.** Average log2 expression levels of *Cxcr5* in purified TCRvβ8+ mPTCL cells vs. wildtype C57bl/6J CD4 T-cells (n=6). *p*=7.91E-07. **E.** Quantification of total B-cells (CD19+B220+) in mPTCL-bearing vs wildtype C57bl/6J mouse spleens (n=6). *p*=8.8E-06. **F** and **G:** Flow cytometry gating for germinal center B-cells and their quantification in mPTCL-bearing vs wildtype C57bl/6J mouse spleens (n=6). Data is from two independent studies using different strategies for gating of GC B-cells: CD38-Fas+ B-cells (**F;** *p*=0.0870) and GL7+Fas+ B-cells (**G;** *p*=0.1949). **H.** Expression of surface immunoglobulin and activation markers on wildtype vs. MHC-II high mPTCL associated B-cells (CD19+B220+). **I.** Serum IgG concentration in wildtype and mPTCL-bearing mice (*p*=0.135, results not statistically significant). **J.** Plasma cell quantification in spleens gated as CD45+B220^{lo}CD138+ cells (n=6). *p*=0.0049. **K.** Kaplan-Meyer survival (time to welfare endpoint) curve of C57bl/6J mice implanted IV (P6 lineage “d”) with varying quantities of splenocytes from mBCL-infiltrated spleens. **L.** PCR for Rosa26-YFP allele and wildtype, floxed or excised *Blimp1* alleles which are present on *Cγ1*-Cre *Blimp1*$^{fl/fl}$ mice (Calado et al. *Cancer Cell* 2010). PCR was performed on DNA extracted from wildtype (wt) C57bl/6J cells, purified T-cells or CD45.2+ cells from mPTCL (T1, T2) and purified B-cells from mBCL (B1, B2). T1 and T2 data are also shown in *Appendix Figure 2G*. **M.** TCRβ+ CD4-T cells (non-malignant) quantification in P6 lineage “d” mBCL and wildtype spleens (n=3; *p=0.0005).* T-test ***p*<0.01, ****p*<0.001. MFI = mean fluorescence intensity.

Appendix Figure S4

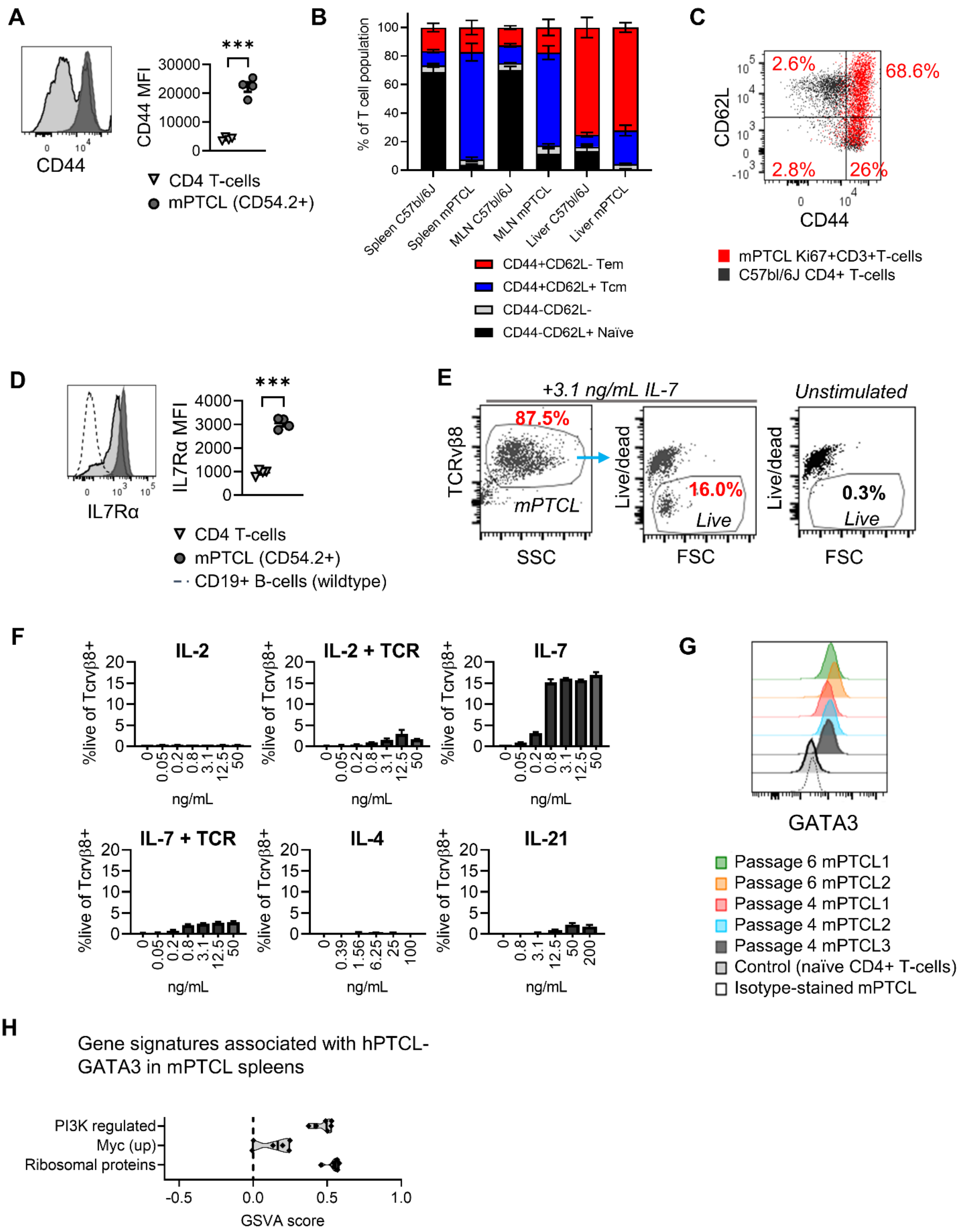

Appendix Figure S4. Resemblance of mPTCL to normal and neoplastic T-cells.

A. Expression levels of CD44 in wildtype CD4+ T-cells vs. CD45.2+ gated PTCL cells expanded in CD45.1 congenic hosts (n=4). Representative histograms are shown *p*=3.47E-05. **B.** The relative frequencies of central memory-like T cells ($CD44^{hi}$ $CD62L^{hi}$) and effector memory-like T cells ($CD44^{hi}$ $CD62L^{lo}$) in wildtype (n=3) vs. mPTCL-infiltrated spleen, MLN and liver (n=5). Total CD4+ T-cells were gated for C57bl/6J spleen, Ki67+ CD8- T-cells gated for mPTCL spleen, with an example of spleen shown in **C,** of CD62L vs. CD44 dot plot. **D.** Expression levels of IL7Ra in wildtype CD4+ T-cells vs. CD45.2+ gated PTCL cells expanded in CD45.1 congenic hosts (n=4). *p*=4.1E-06. Representative histograms are shown against wildtype CD19+ B-cells (dashed line). **E.** 72h *in vitro* culture of mPTCL cells following CD4 T-cell isolation. A fraction of mPTCL cells (TCRvβ8+) survive after 72h in the presence of recombinant murine IL-7. **F.** 72h dose-response effect of homeostatic T-cell cytokines in the presence or absence of TCR stimulation (agonistic antibodies against CD3 and CD28) on *in vitro* survival of TCRvβ8+ mPTCL cells. **G.** MFI of GATA3 expression on CD45.2+ mPTCL cells in congenic CD45.1+ hosts (P4 lineage “a”) or on total T-cells in mPTCL-bearing wildtype hosts (P6 lineage “a”). **H.** Gene set variance analysis (GSVA) scoring for ‘PI3K regulated’, ‘Myc (up)’ and ‘Ribosomal proteins’ gene signatures which were reported by Iqbal et al. *Blood* 2014 as ‘downregulated in PTCL-NOS TBX21 vs. GATA3’. A positive score indicates gene enrichment in a single sample of mPTCL-infiltrated spleen. N=6 scores are shown in truncated violin plots with the median indicated by a central line. MFI = mean fluorescence intensity. ***T-test $p<0.001$.

Appendix Figure S5

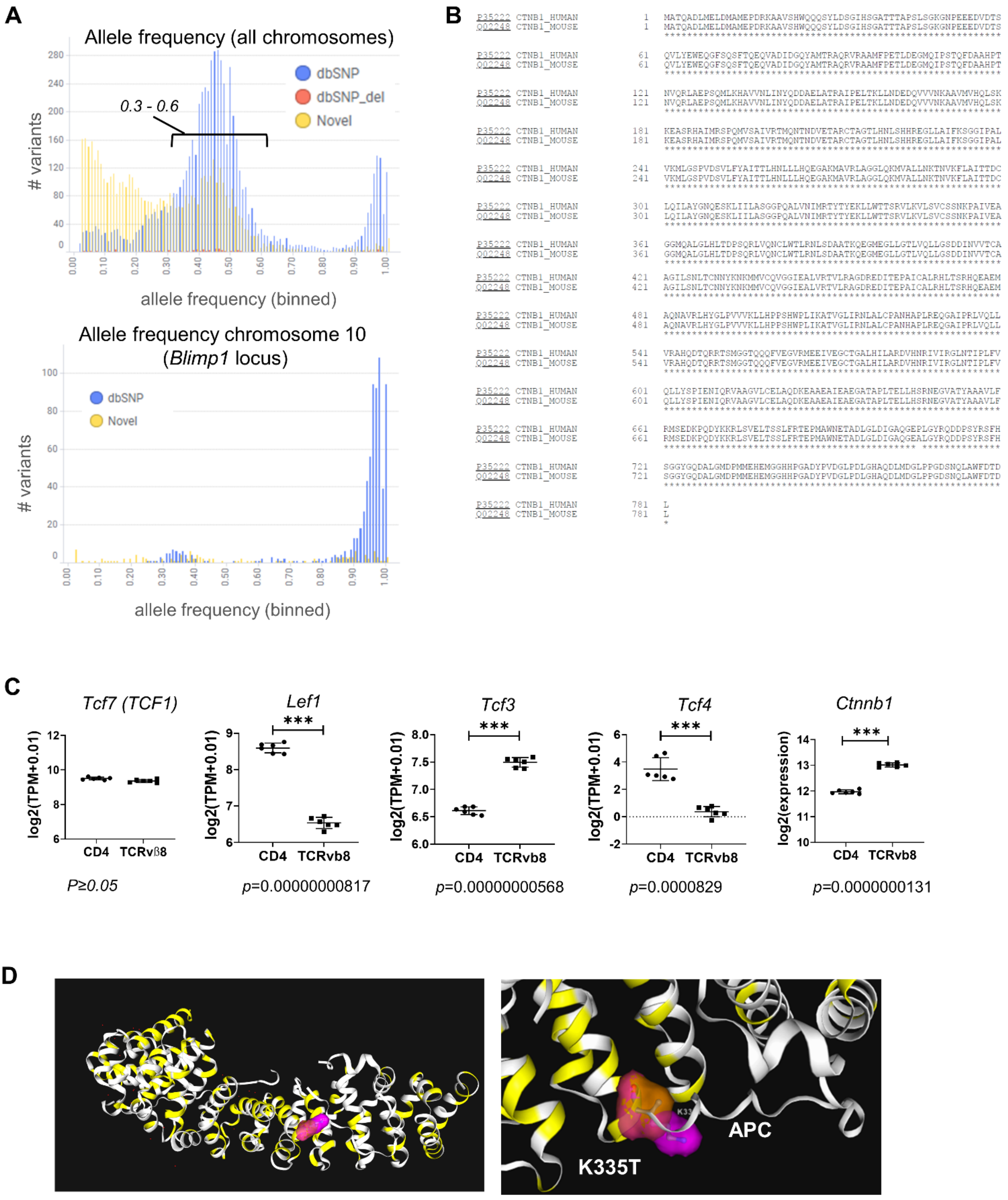

Appendix Figure S5. Detection of an oncogenic mutation in mPTCL.

A. Histogram of allele frequencies of total novel vs. dbSNP-listed exome sequencing variant calls in mPTCL. Shown are variants common to 4 mPTCL and not in wildtype control samples at variant depth ≥4 and preceding filtering on likely somatic mutations. Likely germline variants may be represented by dbSNPs on account that genetic material and associated mouse substrain-specific SNPs of genetically modified mice has a high probability of being retained following backcrossing with wildtype mice. The probability increases for variants in close proximity to targeted transgenes (e.g. *Cγ1* and *Blimp1* loci). A high frequency of dbSNPs on chromosome 10, the location of the homozygous *Blimp1*$^{fl/fl}$ allele, has allele frequencies >0.9 which approximates frequencies for biallelic clonal variants. **B.** Alignment of human and mouse *CTNNB1/CTNB1* (β-catenin) amino acid sequence. Alignment was performed at Uniprot.org. **C.** Expression of β-catenin (*Ctnnb1*) and *Tcf* co-factor gene transcripts in TCRvβ8+ mPTCL cells vs. wildtype CD4 T-cells (n=6). ***Adjusted *p*<0.001. **D.** K335T mutation is localized to the armadillo repeats domain of *Ctnnb1* and is shown in 3D structure crystallized with adenomatous polyposis coli (APC). Data was accessed from COSMIC-3D.

Appendix Figure S6

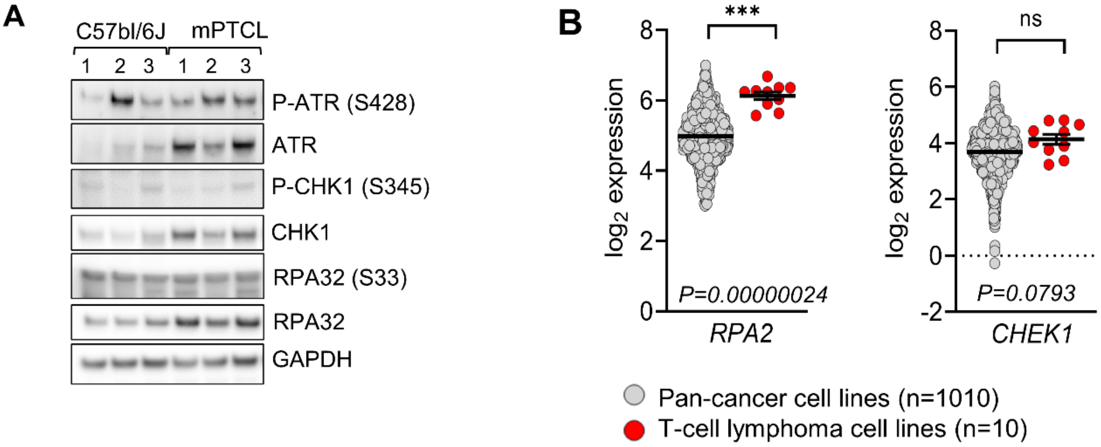

C

Agent	Target	Total # cell lines	IC50 mean (μM)*			IC50 ANOVA	AUC mean*			AUC ANOVA**
			**PTCL (n=3)**	T-ALL (16)	Other	*P-value*	**PTCL (n=3)**	T-ALL (n=16)	Other	*P-value*
AZD6738	ATR	752	**0.430**	1.71	30.1	**0.413**	**0.531**	0.690	0.829	**1.76E-13**
MK-8776	CHK1/2 / CDK2	749	**0.818**	3.50	69.8	**0.119**	**0.722**	0.837	0.922	**8.18E-10**
AZD7762	CHK 1/2	764	**0.103**	0.223	4.13	**0.750**	**0.331**	0.429	0.645	**0.411**
AZD1775	WEE1	770	**0.216**	0.298	6.23	**0.282**	**0.444**	0.470	0.697	**6.20E-14**

**GDSC screen 2*

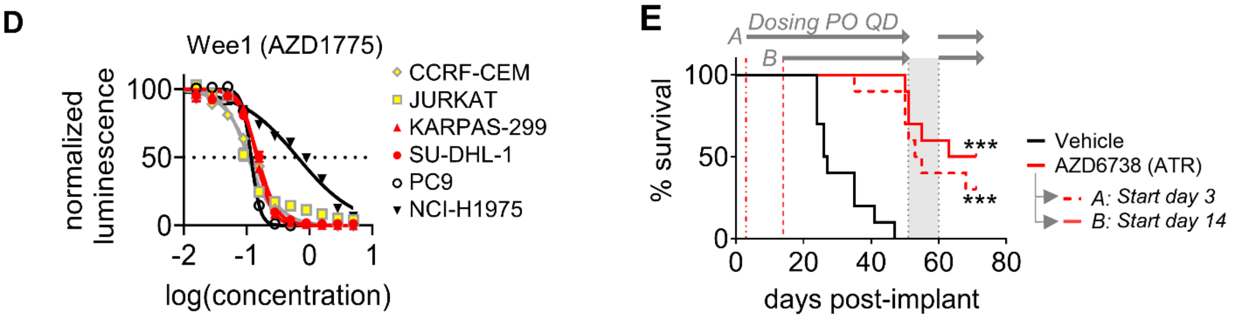

Appendix Figure S6. Targeting the DNA damage response in T-cell malignancies.

A. Western blot for phosphorylated and total ATR (S428), CHK1 (S345) and RPA32 (S33) in control C57bl/6J and mPTCL spleens. GAPDH is a loading control. Three representative samples per group are shown at left, with quantification performed on n=6. **B.** *RPA2* and *CHEK1* log2 normalized gene expression in n=10 T-cell lymphoma cell lines vs. other cancer cell lines (pan-cancer; n=1010). T-test p-values are shown on each graph. Data was accessed from the Cancer Cell Line Encyclopaedia (https://portals.broadinstitute.org/ccle). **C.** Mean IC50 and AUC values for AZD6738, MK-8776, AZD7762 and AZD1775 in T-ALL, PTCL and other cancer cell lines. Data was accessed from Sanger Genomics of Drug Sensitivity in Cancer (GDSC) database. **D.** PTCL (red) and T-ALL (yellow) human cell lines were dosed *in vitro* with AZD1775 Wee1 inhibitor for 72h. Cell proliferation and viability were assayed by luminescence readout using Cell TiterGlo. Data is representative of 3 independent experiments. PC9 and NCI-H197 lung cancer cell lines are DDR inhibitor sensitive and resistant, respectively, and included as controls. **E.** Kaplan-Meyer survival analysis of mice implanted with 10^4 mPTCL cells and dosed with vehicle control (n=10) or AZD6738 50 mg/kg PO QD starting day 3 (A) or day 14 (B) post-implant. A therapy break was issued to mitigate toxicity in some mice, after which therapy was resumed. ***Log-rank test *p*<0.001.
