# Supplementary material for: A preclinical model of peripheral T‐cell lymphoma GATA3 reveals DNA damage response pathway vulnerability
Source: EMBO Mol Med. 2022 May 5;14(6):e15816. doi: 10.15252/emmm.202215816 (PMC9174882; doi:10.15252/emmm.202215816)
Supplement: Supplementary file 9 [file EMMM-14-e15816-s007.pdf]

EMBO Molecular Medicine

A preclinical model of peripheral T-cell lymphoma GATA3 reveals DNA damage response pathway vulnerability

Elizabeth Kuczynski, Giulia Morlino, Alison Peter, Anna Coenen-Stass, Jennifer Moss, Neha Wali, Oona Delpuech, Avinash Reddy, Anisha Solanki, Charles Sinclair, Dinis Calado, and Larissa Carnevalli
DOI: 10.15252/emmm.202215816

Corresponding authors): Elizabeth Kuczynski (elizabeth.kuczynski@astrazeneca.com) , Charles Sinclair (csinclair@flagshippioneering.com)

Review Timeline:

Transfer from Review Commons:	2nd Feb 22
Editorial Decision:	17th Feb 22
Revision Received:	17th Mar 22
Editorial Decision:	28th Mar 22
Revision Received:	5th Apr 22
Accepted:	7th Apr 22

Editor: Lise Roth

Transaction Report:

This manuscript was transferred to EMBO Molecular Medicine following peer review at Review Commons.

(Note: With the exception of the correction of typographical or spelling errors that could be a source of ambiguity, letters and reports are not edited. Depending on transfer agreements, referee reports obtained elsewhere may or may not be included in this compilation. Referee reports are anonymous unless the Referee chooses to sign their reports.)

Review #1

1. How much time do you estimate the authors will need to complete the suggested revisions:

Estimated time to Complete Revisions (Required)

(Decision Recommendation)

Less than 1 month

2. Evidence, reproducibility and clarity:

Evidence, reproducibility and clarity (Required)

This manuscript describes a new preclinical model of peripheral T-cell lymphona and shows that this tumor type is sensitive to ATR inhibitors using both the new preclinical model and human cell lines. This is a well-performed study. The new preclinical model can help advance our understanding of this rare tumor type. Most importantly, this study suggests a potentially new therapy indication for ATR inhibitors. This is relevant, because ATR inhibitors have shown promise in clinical trials, but the cancer types that would benefit most from this type of therapy remain elusive. There are no major issues with this manuscript. I would therefore support publication after minor revisions.

Specific comments (all minor):

1. The Introduction describes various subtypes of PTCL, but only at the end of the Introduction we learn that there are up to 27 subtypes. It would be better, if the authors mentioned in the beginning of the Introduction that there are 27 subtypes, then list the most important subtypes (in just one sentence: "the most important subtypes are: 1) 2) 3) etc") and then describe briefly each one of the most important subtypes.
2. In the Introduction, the authors state that "ATR inhibition was previously shown to be well-tolerated in the clinic [28]", but cite only on reference of their own ATR inhibitor. I think that the authors should have a paragraph describing the most important clinical trials of ATR inhibitors and cite the studies of the Merck and Bayer ATR inhibitors, as well. It is important to mention that ATR inhibitors have efficacy (see for example, Yap et al 2020), but also to highlight the issue of toxicity, which limits the dose that can be used in the clinic. It is for this reason that it is important to identify tumor types that respond well to ATR inhibitors.
3. Materials and Methods: In vivo studies. This section is not very clear. Perhaps, a figure would help and be part of the main figures, not the supplement. The text here can also be improved.
4. Figs 1-5 characterize the lymphoma as a PTCL. No concerns, but I wonder if 5 figures are needed. Perhaps, some material can be moved to the supplement.
5. Figs 6-7 show that the PTCLs exhibit a DNA damage response and that treatment with the ATR inhibitor prolongs survival of mice implanted with the PTCL. The effect of the

ATR inhibitor is quite good (Fig. 7D) and could justify clinical trials in human PTCL patients.

3. Significance:

Significance (Required)

See above.

Referee Cross-commenting

I find some of the requests for major new experiments by reviewers 2 and 3 as being too onerous for the authors. The main conclusion in terms of clinical significance is that this tumor type may respond to ATR inhibitors. This can only be tested in the clinic reliably. The data presented already justify enrolling a few patients with this tumor type in ongoing phase I/II studies of ATR inhibitors.

Review #2

1. How much time do you estimate the authors will need to complete the suggested revisions:

Estimated time to Complete Revisions (Required)

(Decision Recommendation)

Between 1 and 3 months

2. Evidence, reproducibility and clarity:

Evidence, reproducibility and clarity (Required)

The authors presented a nice and comprehensive work which is well performed and described. The experimental part consists of broad spectrum of advanced techniques. The interpretation of the many data generated is very clear, however additional aspects needs to be considered.

In the abstract, the description is comprehensible for experts in this specific field. For readers less deeply involved in this topic, the combined consideration of B- and T-cell lymphomas without further explanation might raise confusion. Of course, I am well aware of a potential co-existence and additional development of B-cell lymphoma under similar conditions, the wording should be more explanatory - despite length restrictions.

As very correctly stated by the authors, T-cell lymphoma is a very heterogenous disease entity which is due to morphology, immunophenotype and molecular characteristics. Most likely, these differences is not only a reflections of their features but also of their pathogenesis. The disease model described in the manuscript appears comprehensible and raises not much criticism. But the quite broad and general extension to more or less all type of T-cell lymphoma should be reconsidered. In fact, the findings are mainly/exclusively related to T-cell lymphoma GATA3 and general statements such as "This work provides mechanistic insight into the molecular and 16 immunological drivers of T-cell lymphomagenesis ..." should avoided. This is also due to the suggested potential therapeutic options which might also only relevant for the above mentioned T-cell lymphoma subtype. A central result of this work was the beneficial therapeutical role of DDR inhibitors based on DNA replication deficiencies and genomic instability. However, despite the many findings described, this specific (and important) is not demonstrated on the molecular level. Finally, the topic „T-cell clonality" is confusingly described. Whereas the material and methods section is merely dealing with the detection of the TCRVß using by parallel sequencing, the data of the results section are also based on the CDR3 proportion of the rearranged TCR genes or a combination of TCRVß usage and CDR composition. This is very confusing and lacks a clear definition of clonality. A biased TCRVß usage is not sufficient; T-cell clonality is defined by the uniqueness of the combination of Vß, Dß and Jß usage in conjunction with the CDR3 DNA sequence.

3. Significance:

Significance (Required)

There is clear advance and extension of knowledge based on the data presented. There is also potential high significance if the suggested treatment option (DDR inhibition) can be confirmed and is further substantiated by clinical data. My expertise is based on a long history in the research dealing with molecular mechanisms in malignant lymphoma, including T-cell lymphoma.

Review #3

1. How much time do you estimate the authors will need to complete the suggested revisions:

Estimated time to Complete Revisions (Required)

(Decision Recommendation)

Between 3 and 6 months

2. Evidence, reproducibility and clarity:

Evidence, reproducibility and clarity (Required)

Summary:

In the current manuscript, Kuczynski et al establish and characterize a transplantable mouse model of peripheral T-cell lymphoma (PTCL), in an immunocompetent context, which resembles human PTCL-GATA3. The lymphoma did not result from direct genetic manipulation but rather spontaneously developed in bystander cells from mice bearing germinal center-restricted Blimp1 deletion. The mouse PTCL (mPTCL) cells display features of human PTCL and of Tfh cells and also a profile indicative of a Th2 phenotype, similar to that previously reported in p53-/- VAV1 transgenic mice that also developed PTCL-GATA3-like disease. MYC activation is observed and associated with replication stress (although this was not formally demonstrated), whereas PI3K/Akt pathway activation was hinted from pathway enrichment analysis but shown not to be relevant for the maintenance of the disease as demonstrated by the lack of in vivo effect of two small molecule inhibitors of PI3K/Akt pathway. The relevance of the model for human disease is underlined by observations that include the presence of a mutation in Ctnnb1 (encoding beta-catenin) that is present in some PTCL patients. Importantly, the authors provide evidence that replication stress may be an Achilles' heel that can be explored therapeutically by the use of ATR and CHK inhibitors. This is of particular potential importance in a malignancy that remains a clinical challenge.

Major comments:

1) The claim that the mPTCL cells resemble Tfh could be better substantiated. The immunophenotype of mPTCL cells (Fig 3A), indicative of Tfh origin, does not appear to be in agreement with the Cibersort RNAseq deconvolution data in Fig 3C, where CD4 follicular T cells appear to be a minor population as compared to naïve and memory CD4 T cells. Can the authors clarify why this apparent discrepancy? The data in 3C are from cells are from day 21 post transplant. Which passage? What about in 3A, which day post transplant and which passage? Importantly, in Fig S3 please include all the subsets that are analyzed in Fig 3C, including evidently the different CD4 T cell subsets that were deconvoluted. I would suggest presenting immunophenotypic data from different time points post transplant and/or different passages to make the data supporting the Tfh origin of the mPTCL more robust. Also, it would be critical to evaluate CXCR5 and demonstrate the lymphoma cells are BCL6+ CXCR5+. Finally, the authors should use the RNAseq data of sorted mPTCL cells that they have already generated (Fig 6A,E) to perform for instance GSVA comparing different T cell subsets to provide further evidene that the lymphoma cells are derived from/resemble Tfh cells.

2) The claim that "mPTCL has a Tfh/Th2 cell phenotype" is intriguing. It seems to be based mainly on gene expression data of bulk cells and a few immunophenotypic markers that are not analyzed simultaneously. So, not clear if the mPTCL is a mix of Tfh-like cells and Th2-like cells or a single population with an aberrant phenotype that has features of both. The authors should perform a deeper flow cytometry analysis with

markers of both populations in order to better characterize the actual phenotype and possible heterogeneity (or lack thereof) of the lymphoma cells.

3) The biological rationale for exploring ATR/CHK1 inhibitors should be made stronger. Clearer evidence of replication stress in mPTCL and human PTCL cells (cell lines, and if possible PDX or patient-derived cell lines) should be presented. This could be easily done by performing western blot analysis of indicators of replication stress besides γ-H2AX, e.g. phospho-ATR, phospho-CHK1, phospho-RPA.

4) KARPAS 299 and SU-DHL-1 cells are ALK+ ALCL cell lines, which do not correspond to the PTCL cell type(s) that the mPTCL model more closely appears to resemble: PTCL-GATA3 or PTCL-TFH. The therapeutic significance of their studies would be considerably improved if they managed to use a human PDX (e.g. from PROXe) or patient-derived cell lines (e.g. see Am J Hematol 2017 Dec;92(12):1287-1294) closer to these entities (or at least more representative of the heterogeneity of PTCL in humans) for their in vivo and/or in vitro drug-related studies.
Performing the studies indicated in 4) would likely require getting PDX or patient-derived cell lines from external sources and then performing at least in vitro experiments (quick) or in vivo studies (which may take up to a couple of months). Altogether, at least 2-4 months would be expectable to address this point, which nonetheless would be highly significant in broadening the potential translational/clinical relevance of the manuscript. The other 3 critiques can be addressed in a considerably shorter period of time.

Minor comments:

1) Why were differences considered statistically significant for P=0.05? This is an unusual criterion that should be properly justified or changed to P<0.05 and data reanalyzed accordingly.

2) Please indicate the number mice/replicates analyzed in the IHC data presented throughout the manuscript.

3) The authors show clearly in Fig 2 that, after transfer, the tumor that arises is a PTCL. However, the way in which the main text is written regarding the neoplasia in the original mouse and establishment of the PTCL line in the first section of the results ("Isolation of an in vivo transplantable murine lymphoma") and how Fig 1A and Supp Fig 1 were formulated are confusing. Was the mouse #2695 (from which the splenocytes were collected that eventually originated the line in B6 mice) originally catalogued as displaying ABC DLBCL? From Fig S1 it is dubious if the mouse had lymphoma or not (mere B-cell hyperplasia leading to splenomegaly?) but it seems not (based on the color code used for mBCL and mPTCL), whereas from Fig 1A it seems probably yes (since the donor mouse, 2695, from which the spleen is collected is referred to as has having a "lymphoma" - of which type?). Please clarify and reformulate text and figures to make this clear. This is not mere semantics, since it is important to understand whether the mPTCL originated from a minor population of bystander T cells that were present in an otherwise DLBCL or B-cell "hyperplasia" in the original mouse or whether this mouse was an exception that developed already bystander PTCL (or T cell proliferative disease) originally. Also, clarify in the legend what the grey x black

colors mean in the mice depicted in Fig 1A.

4) For better clarity, instead of columns and (possibly) standard deviation (or is it standard error of mean?) in Figs such as 1G, 3A,D,E,H,I or 4C, the authors should present data as dot plots.

5) In Fig 1B, although the authors do not make any claims regarding number of transplanted cells and disease 'kinetics', please indicate whether the survival curves for the different transplant cell numbers were statistically different or not. Either way the information is important.

6) Line 194: where it is "Fig S1D" it should be, I believe, "Fig. S2C".

7) mBCL arising in the context of mPTCL appears to be of Blimp1 wild type origin. So, the mBCL cells should be CD45.1. Was this the case?

8) The identity of all the 3 PTCL cell lines should be indicated.

9) In the main text the authors mention they tested 6 T-ALL cell lines but the corresponding figure (Fig B) only shows 5 cell lines. Please correct main text or include the missing cell line in the figure.

3. Significance:

Significance (Required)

The significance of the studies presented in the manuscript by Kuczynski et al lies mainly on 3 aspects:

1) the generation (and deep characterization) of a novel in vivo model of PTCL, which may help advance the understanding of the underlying biology while providing a tool for translational studies. The authors provide good evidence that the model recapitulates several features of human PTCL, and particularly of PTCL-GATA3. The existence of other models, including of PTCL-GATA3, does not, in my view, decrease the relevance of the current studies, since the strategies to generate them are considerably different and thus the information on etiology of the disease and its heterogeneity will be complementary to previous studies.

2) the demonstration that replication stress occurs in the mPTCL (which should be made more robust and complemented with data from human PTCL but is exciting)

3) the demonstration that replication stress can be exploited therapeutically by the use of small molecule inhibitors of ATR and CHK1/2. This, if properly complemented with stronger human data, not only validates the importance of the model for human PTCL but is in itself a potentially major step forward for the targeting of PTCL cells.
Given its characteristics, the model may be especially proper also for further characterization of the crosstalk between GC B cells and Tfh cells in their ability to drive mature B and T cell lymphomas.
Overall, the studies should be of interest to both basic researchers and clinicians

working in PTCL in particular, and also DLBCL, as well as other hematological malignancies.

Reviewer expertise: T-cell leukemia; signaling; mouse models

Full Revision

Manuscript number: RC-2021-00947
Corresponding author(s): Kuczynski, Elizabeth

[Please use this template only if the submitted manuscript should be considered by the affiliate journal as a full revision in response to the points raised by the reviewers.

If you wish to submit a preliminary revision with a revision plan, please use our "Revision Plan" template. ***It is important to use the appropriate template to clearly inform the editors of your intentions.****]*

1. General Statements [optional]

Please see letter on page 2.

Full Revision

2 February 2022

Sara Monaco
Managing Editor
Review Commons

Re: Reviewer comments

Dear Dr. Monaco,

I am writing on behalf of the authors, in response to your letter and the reviewer comments to our manuscript '**A preclinical model of peripheral T-cell lymphoma GATA reveals DNA damage response pathway vulnerability**'.

We would like to thank the Review Commons editorial team & the reviewers for consideration of our manuscript and the very thoughtful comments. We were encouraged by the positive feedback and enthusiasm shown by all three reviewers, who highlight both the novelty of our preclinical research on PTCL and potential therapeutic implications of our work.

I have therefore enclosed a revised manuscript, which we feel has been greatly strengthened by incorporating major suggestions from the reviewers. Our revised manuscript includes the following additions:

- Profiling of the molecular phenotype of the mPTCL, which provides further support that intrinsic DNA damage/replication stress likely underlies model sensitivity to ATR inhibition.
- Further analysis of the clonal phenotypes of the mPTCL, which substantiates its use as a model of the PTCL-GATA3 subtype.
- Numerous minor additions and clarifications to the text as suggested by the reviewers, which improve the clarity and flow of the manuscript.

I have also appended a point-by-point response to reviewers comments, and am hopeful that our revisions address any outstanding concerns.

We look forwards to hearing positively about the next steps for our manuscript. Please do not hesitate to get in touch for any further matters relating to our paper.

Yours Sincerely,

Dr. Elizabeth A. Kuczynski

2. Point-by-point description of the revisions

Reviewer #1 (Evidence, reproducibility and clarity (Required)):

This manuscript describes a new preclinical model of peripheral T-cell lymphona and shows that this tumor type is sensitive to ATR inhibitors using both the new preclinical model and human cell lines. This is a well-performed study. The new preclinical model can help advance our understanding of this rare tumor type. Most importantly, this study suggests a potentially new therapy indication for ATR inhibitors. This is relevant, because ATR inhibitors have shown promise in clinical trials, but the cancer types that would benefit most from this type of therapy remain elusive. There are no major issues with this manuscript. I would therefore support publication after minor revisions.

Specific comments (all minor):

1. The Introduction describes various subtypes of PTCL, but only at the end of the Introduction we learn that there are up to 27 subtypes. It would be better, if the authors mentioned in the beginning of the Introduction that there are 27 subtypes, then list the most important subtypes (in just one sentence: "the most important subtypes are: 1) 2) 3) etc") and then describe briefly each one of the most important subtypes.

> **Author comment 1:** Discussion of the PTCL subtypes and most important subtypes are now introduced in the first paragraph of the Introduction.

2. In the Introduction, the authors state that "ATR inhibition was previously shown to be well-tolerated in the clinic [28]", but cite only on reference of their own ATR inhibitor. I think that the authors should have a paragraph describing the most important clinical trials of ATR inhibitors and cite the studies of the Merck and Bayer ATR inhibitors, as well. It is important to mention that ATR inhibitors have efficacy (see for example, Yap et al 2020), but also to highlight the issue of toxicity, which limits the dose that can be used in the clinic. It is for this reason that it is important to identify tumor types that respond well to ATR inhibitors.

> **Author comment 2:** Suggested references for both Merck and Bayer ATR inhibitors (Yap et al., 2020; Yap et al., 2021) have now been cited in the introduction (5th paragraph, final sentence). We also highlight the issue of toxicity of continuous dose schedules in the discussion (paragraph 7).

3. Materials and Methods: In vivo studies. This section is not very clear. Perhaps, a figure would help and be part of the main figures, not the supplement. The text here can also be improved.

Author comment 3: We have revised the Materials and Methods section to incorporate a more detailed description of the *in vivo* study methodology and tumor passaging (subheading "*In vivo* studies"). In addition, we have modified Fig. 1A in the manuscript to enhance the clarity of the transplant methodology during the initial model development.

Briefly, a lymphoma originated from Cγ-Cre *Blimp1*$^{fl/fl}$ mice (Calado et al., 2010) and was transplantable to WT mice. Early transfers were recorded across recipients and passages to ensure traceability of the model development, yielding three independent and phenotypically stable lineages of the T-cell lymphoma by passage 4 (termed lineages "a", "b", "c", and "d").

We also identified a single recipient mouse at the 4th passage of cells with an established B-cell lymphoma (termed lineage "d"), which was traced to an earlier passage. This is discussed in detail in the results section (Results section "mPTCL phenotypically and functionally resemble T-follicular helper cells", and Fig. 3d). Importantly, B-cell lymphoma cells have never been identified in transplanted lymphomas arising from the "a", "b", or "c" lineages.

4. Figs 1-5 characterize the lymphoma as a PTCL. No concerns, but I wonder if 5 figures are needed. Perhaps, some material can be moved to the supplement.

Author comment 4: We feel that a major strength of the manuscript has been the depth of the analysis performed, which is essential given the high novelty of the mPTCL model. This is especially pertinent considering that diverse researchers may have distinct interests and areas of focus. We therefore request further guidance and recommendations from the editorial team around moving any of our main figures to supplementary information.

5. Figs 6-7 show that the PTCLs exhibit a DNA damage response and that treatment with the ATR inhibitor prolongs survival of mice implanted with the PTCL. The effect of the ATR inhibitor is quite good (Fig. 7D) and could justify clinical trials in human PTCL patients.

Author comment 5: We thank the reviewer for highlighting this major finding from our paper. We are in process of engaging with clinical trial physicians who have interest in PTCL and the publication of the work will further support the development of those trials.

Reviewer #1 (Significance (Required)):

See above.

Referee Cross-commenting

I find some of the requests for major new experiments by reviewers 2 and 3 as being too onerous for the authors. The main conclusion in terms of clinical significance is that this tumor type may respond to ATR inhibitors. This can only be tested in the clinic reliably. The data presented already justify enrolling a few patients with this tumor type in ongoing phase I/II studies of ATR inhibitors.

Reviewer #2 (Evidence, reproducibility and clarity (Required)):

The authors presented a nice and comprehensive work which is well performed and described. The experimental part consists of broad spectrum of advanced techniques. The interpretation of the many data generated is very clear, however additional aspects needs to be considered.

6. In the abstract, the description is comprehensible for experts in this specific field. For readers less deeply involved in this topic, the combined consideration of B- and T-cell lymphomas without further explanation might raise confusion. Of course, I am well aware of a potential co-existence and additional development of B-cell lymphoma under similar conditions, the wording should be more explanatory - despite length restrictions.

> **Author comment 6:** We have revised the abstract accordingly, with the following:
>
> *"Human PTCL is often associated with B-cell hyperplasia and coincident B-cell lymphomagenesis. We observed coincident B-cell activation and an example of a de novo B-cell lymphoma development in the mPTCL model."*

7. As very correctly stated by the authors, T-cell lymphoma is a very heterogenous disease entity which is due to morphology, immunophenotype and molecular characteristics. Most likely, these differences is not only a reflections of their features but also of their pathogenesis. The disease model described in the manuscript appears comprehensible and raises not much criticism. But the quite broad and general extension to more or less all type of T-cell lymphoma should be reconsidered. In fact, the findings are mainly/exclusively related to T-cell lymphoma GATA3 and general statements such as "This work provides mechanistic insight into the molecular and 16 immunological drivers of T-cell lymphomagenesis ..." should avoided. This is also due to the suggested potential therapeutic options which might also only relevant for the above mentioned T-cell lymphoma subtype.

Author comment 7: The relevant text in the discussion has been revised to clarify that the data presented in the work directly suggests a benefit of ATRi primarily to PTCL-GATA3 and that further work is required to understand the relevance of ATRi for the treatment of other PTCL subtypes (paragraph 7):

"Given the urgency for effective PTCL treatments, ATR inhibition therapy may represent an opportunity for immediate application to PTCL-GATA3 patients. We do not exclude that a broader range of PTCL might be eligible for a DDR treatment strategy, but this requires further investigation using appropriate models of other PTCL subtypes."

In addition, the final statement in the Results now refers to ATR inhibition in PTCL-GATA3:

*"In summary, the analysis of mPTCL uncovered a DDR vulnerability, providing rationale for ATR inhibition therapy for human PTCL-**GATA3** treatment.*

8. A central result of this work was the beneficial therapeutical role of DDR inhibitors based on DNA replication deficiencies and genomic instability. However, despite the many findings described, this specific (and important) is not demonstrated on the molecular level.

Author comment 8: The reviewer raises an important conceptual point that is pertinent to the entire field of DNA damage response therapeutics. At present, there is no unifying molecular biomarker that is specifically indicative of DNA replication deficiency, genomic instability or replication stress.

In the manuscript, we present data showing that MYC is overexpressed in the mPTCL model. MYC is well established to drive tumor proliferation and has been strongly implicated also as a driver of DNA replication stress (Dominguez-Sola et al., 2007; Kotsantis et al., 2018). Whilst MYC can drive cells towards a more proliferative/replicative stress state, we agree with the reviewer that this is not definitive. In the original submitted manuscript, we had therefore included evidence that ATR-dependent gene expression profiles are elevated, which implicates the activation of the ATR pathway (Fig. 6F). We also included data showing evidence that γH2AX was elevated in splenocytes from mPTCL mice (Figs. 6G, 6H), which indicates high levels of DNA damage at the molecular level.

To further support the presence of elevated DDR responses and replicative stress in mPTCL cells, we have now performed additional Western blotting analysis of splenocytes (Fig. 6H).

The data in the revised manuscript also shows a trend for induction of CHK1 expression in mPTCL splenocytes and a significant induction of RPA32 at a molecular level,

validating previous gene expression data. CHK1, the key effector of ATR pathway, is overexpressed in T-ALL cell lines and patients, and is linked to ATR/CHK1 pathway sensitivity (Iacobucci et al., 2015; Sarmento et al., 2015). Elevated RPA pools are essential to respond to excessive ssDNA during the replication stress response (Toledo et al., 2013). Thus, our data aligns with the observed efficacy of ATR inhibitors in the mPTCL.

9. Finally, the topic „T-cell clonality" is confusingly described. Whereas the material and methods section is merely dealing with the detection of the TCRVß using by parallel sequencing, the data of the results section are also based on the CDR3 proportion of the rearranged TCR genes or a combination of TCRVß usage and CDR composition. This is very confusing and lacks a clear definition of clonality. A biased TCRVß usage is not sufficient; T-cell clonality is defined by the uniqueness of the combination of Vß, Dß and Jß usage in conjunction with the CDR3 DNA sequence.

Author comment 9: We agree with the reviewer's precise assertions of the challenges to measure T-cell clonality, it is important to clarify the nature of our use of TCR Vß and orthogonal TCR sequencing to determine the clonal origin of tumor cells. We took advantage of the availability of TCR Vß antibodies and TCR sequencing to provide evidence that the mPTCL tumors themselves were derived from a clonal origin, an approach taken by several other groups (Cortes et al., 2018; Pechloff et al., 2010; van Dongen et al., 2003). The same TCR Vß8 clone was expanded consistently across passages and in discrete tissues (Fig. 2C, 2E), which strongly implicated a monoclonal expansion of tumor cells. Moreover, we confirmed using an orthogonal sequencing method that Vß and VJ regions were monoclonal as suggested by the reviewer. This data was included in our original submission (Fig 2D).

In the results section, "Characterization of *Cγ1*-Cre *Blimp1*$^{fl/fl}$-derived T-cell transplantable lymphoma", paragraph 2, we have revised the text for clarity, stating that "*Human (h)PTCLs derive from a* ***clonal origin*** *as indicated by frequent clonal TCRα/β rearrangements [35]*".

We have also amended the Materials and Methods section to avoid confusion as to the techniques used to measure the clonal origin of tumors. We firstly, concatenated the TCR Vß flow cytometry methodology into the flow cytometry section. Secondly, we included a separate section on methods for orthogonal TCR sequencing.

Importantly, both approaches are highly concordant, supporting a clonal origin of the mPTCL cells from a Vß8 (encoded by *Trbv 13-2*) and *Tcbj1-2* expressing progenitor cell. Our data exclude the possibilities of a 'transplantable hyperplasia', or multiple distinct lineages of PTCL cells.

Reviewer #2 (Significance (Required)):

There is clear advance and extension of knowledge based on the data presented. There is also potential high significance if the suggested treatment option (DDR inhibition) can be confirmed and is further substantiated by clinical data. My expertise is based on a long history in the research dealing with molecular mechanisms in malignant lymphoma, including T-cell lymphoma.

Reviewer #3 (Evidence, reproducibility and clarity (Required)):

Summary:

In the current manuscript, Kuczynski et al establish and characterize a transplantable mouse model of peripheral T-cell lymphoma (PTCL), in an immunocompetent context, which resembles human PTCL-GATA3. The lymphoma did not result from direct genetic manipulation but rather spontaneously developed in bystander cells from mice bearing germinal center-restricted Blimp1 deletion. The mouse PTCL (mPTCL) cells display features of human PTCL and of Tfh cells and also a profile indicative of a Th2 phenotype, similar to that previously reported in p53-/- VAV1 transgenic mice that also developed PTCL-GATA3-like disease. MYC activation is observed and associated with replication stress (although this was not formally demonstrated), whereas PI3K/Akt pathway activation was hinted from pathway enrichment analysis but shown not to be relevant for the maintenance of the disease as demonstrated by the lack of in vivo effect of two small molecule inhibitors of PI3K/Akt pathway. The relevance of the model for human disease is underlined by observations that include the presence of a mutation in Ctnnb1 (encoding beta-catenin) that is present in some PTCL patients. Importantly, the authors provide evidence that replication stress may be an Achilles' heel that can be explored therapeutically by the use of ATR and CHK inhibitors. This is of particular potential importance in a malignancy that remains a clinical challenge.

Major comments:

10. The claim that the mPTCL cells resemble Tfh could be better substantiated. The immunophenotype of mPTCL cells (Fig 3A), indicative of Tfh origin, does not appear to be in agreement with the Cibersort RNAseq deconvolution data in Fig 3C, where CD4 follicular T cells appear to be a minor population as compared to naïve and memory CD4 T cells. Can the authors clarify why this apparent discrepancy?

> **Author comment 10:** A significant finding in the work is that the murine lymphoma resembles a model of PTCL-GATA3, and the model additionally harbors lineage-defining biomarkers of Tfh cells. This is directly substantiated by characterization of the major

phenotypic markers of Tfh, namely the presence of ICOS, CD40L, BCL6 and PD1 (Fig. 3A).

The Cibersort algorithm is based on inference of RNAseq data to gene expression profiles of healthy immune cell populations rather than tumor cells, many of which have overlapping or closely related gene expression profiles. It is likely that during its origination or in the process of oncogenesis, the mPTCL gene expression diverged significantly from a ‘pure’ Tfh signature, and thus is being incorrectly categorized by the Cibersort algorithm. Indeed, we did not observe the presence of the Tfh chemokine receptor CXCR5 on lymphoma cells (Fig. S3A), which further suggests their difference from a normal Tfh population.

Although the Cibersort data is supportive of the T-cell origin of the lymphoma, we wish to avoid any confusion, particularly around claims that the mPTCL may have originated from a Tfh cell of origin. We have therefore removed the Cibersort panel from Fig. 3 in the manuscript and corresponding results section. The deleted figure is shown below.

We have also amended the first paragraph in the results section **“mPTCL phenotypically and functionally resemble T-follicular helper cells”**. Importantly, we cannot make strong claims as to the definitive origin of the mPTCL cells as being Tfh derived, merely that they share many functional features of Tfh cells.

Removed Figure 3C:

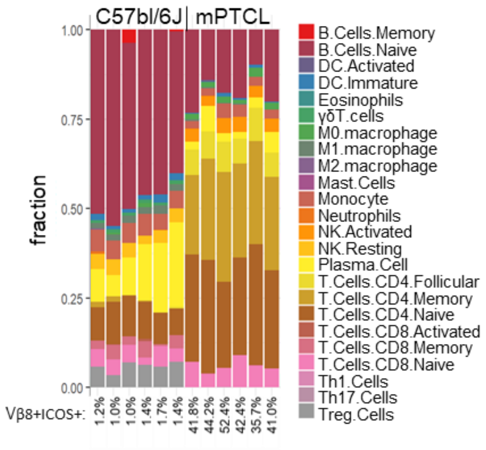

11. The data in 3C are from cells are from day 21 post transplant. Which passage? What about in 3A, which day post transplant and which passage?

Author comment 11: The data in Fig. 3A are from the 4th passage of mPTCL, and represent lineage “a” cells (Fig. S1). In this experiment, mice were implanted with mPTCL and analyzed when they reached the welfare endpoint (between days 31-36 post-implant). This has been clarified in the figure legend.

Review COMMONS

In the original Fig. 3C, cells were from the 5th passage of lineage “a” cells (Fig. S1). These cells were analyzed on day 21 post-implantation. Please note that based on our response to comment 10, we are recommending to remove panel Fig. 3C from our updated manuscript.

12. Importantly, in Fig S3 please include all the subsets that are analyzed in Fig 3C, including evidently the different CD4 T cell subsets that were deconvoluted.

Author comment 12: Based on previous comments raised by reviewer 3 (comment 10 & comment 11), we have removed Cibersort analysis depicted in Figs. S3 and 3C from the manuscript. The requested data and graphs are provided below for the reviewer.

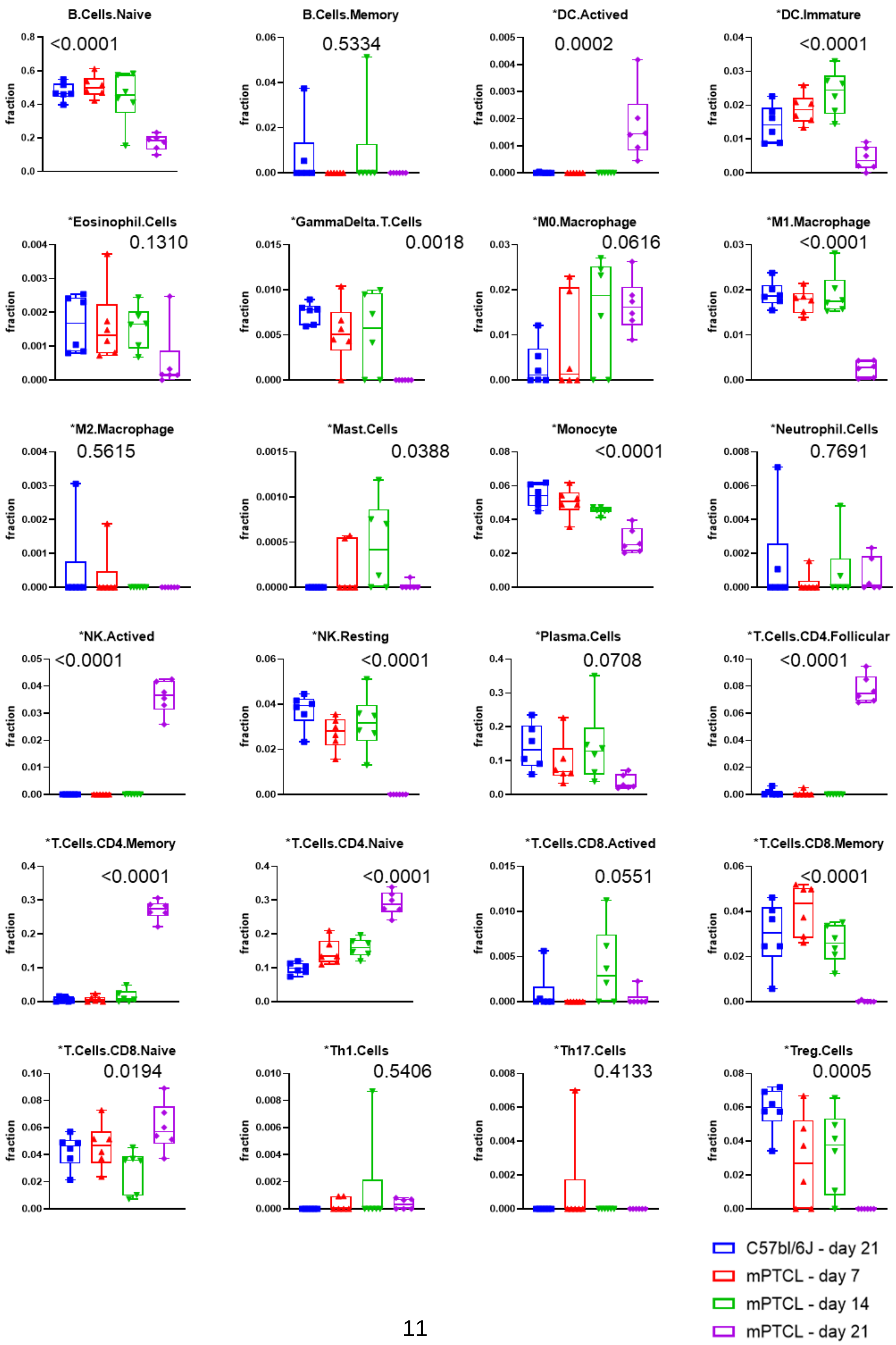
B.Cells.Naive
<0.0001
B.Cells.Memory
0.5334
*DC.Actived
0.0002
*DC.Immature
<0.0001
*Eosinophil.Cells
0.1310
*GammaDelta.T.Cells
0.0018
*M0.Macrophage
0.0616
*M1.Macrophage
<0.0001
*M2.Macrophage
0.5615
*Mast.Cells
0.0388
*Monocyte
<0.0001
*Neutrophil.Cells
0.7691
*NK.Actived
<0.0001
*NK.Resting
<0.0001
*Plasma.Cells
0.0708
*T.Cells.CD4.Follicular
<0.0001
*T.Cells.CD4.Memory
<0.0001
*T.Cells.CD4.Naive
<0.0001
*T.Cells.CD8.Actived
0.0551
*T.Cells.CD8.Memory
<0.0001
*T.Cells.CD8.Naive
0.0194
*Th1.Cells
0.5406
*Th17.Cells
0.4133
*Treg.Cells
0.0005
fraction
C57bl/6J - day 21
mPTCL - day 7
mPTCL - day 14
mPTCL - day 21

13. I would suggest presenting immunophenotypic data from different time points post transplant and/or different passages to make the data supporting the Tfh origin of the mPTCL more robust.

Author comment 13: We thank the reviewer for this helpful suggestion, as this information lends further weight to the stability of the mPTCL model over time. We have now included analysis showing stability of the Tfh markers PD-1 and ICOS across passages 2, 3 and 6 (Fig. S3C). The following text has also been included in the first paragraph of "**mPTCL phenotypically and functionally resemble T-follicular helper cells**" in the results section:

'Expression of several Tfh markers suggested a potential Tfh cell lymphoma origin, which was reinforced by the finding that expression of PD1 and ICOS was ubiquitous across tumor cells and maintained throughout passaging (**Fig. S3C**).'

14. Also, it would be critical to evaluate CXCR5 and demonstrate the lymphoma cells are BCL6+ CXCR5+.

Author comment 14: The mPTCL cells do not express CXCR5 as shown by flow cytometry and gene expression analysis, and we have now included these data in Figs. S3A, S3B.

15. Finally, the authors should use the RNAseq data of sorted mPTCL cells that they have already generated (Fig 6A,E) to perform for instance GSVA comparing different T cell subsets to provide further evidence that the lymphoma cells are derived from/resemble Tfh cells.

Author comment 15: GSEA analysis on sorted mPTCL cells was included in the original submission (Fig. 4A). This analysis shows that mPTCL has activated and effector memory CD4+ and Th2 features. Tfh signatures are not significantly detected with this methodology.

We would like to reiterate that the mPTCL shared lineage defining biomarkers of Tfh cells and newly incorporated data in Fig. S3C shows maintenance of ICOS and PD-1 expression from the earliest passage profiled (P2). While these data suggest the possibility that a bone fide Tfh cell represented the cell of origin of the tumor, we do not wish to make strong claims in this respect.

16. The claim that "mPTCL has a Tfh/Th2 cell phenotype" is intriguing. It seems to be based mainly on gene expression data of bulk cells and a few immunophenotypic markers that are not analyzed simultaneously. So, not clear if the mPTCL is a mix of Tfh-like cells and Th2-like cells or a single population with an aberrant phenotype that has features of both. The authors should

perform a deeper flow cytometry analysis with markers of both populations in order to better characterize the actual phenotype and possible heterogeneity (or lack thereof) of the lymphoma cells.

Author comment 16: The newly added Fig. S3C shows that the mPTCL cells are stable across different passages in terms of their expression of Tfh markers PD1 and ICOS. This figure additionally reveals the synchronous expression of the Tfh markers PD1 and ICOS across all mPTCL cells.

We have additionally included analysis of the lineage defining transcription factor of Th2 cells, GATA3, which was also expressed across all mPTCL cells (Fig. S4G). Collectively our data supports a lack of heterogeneity within the mPTCL cells, Tfh and the Th2-associated markers that we have examined as these are synchronously expressed.

17. The biological rationale for exploring ATR/CHK1 inhibitors should be made stronger. Clearer evidence of replication stress in mPTCL and human PTCL cells (cell lines, and if possible PDX or patient-derived cell lines) should be presented. This could be easily done by performing western blot analysis of indicators of replication stress besides γ-H2AX, e.g. phospho-ATR, phospho-CHK1, phospho-RPA.

Author comment 17: We thank the reviewer for this suggestion. We completed this analysis on the mPTCL cells and human PTCL cell lines (presented in Figs. 6H, S6A and below) showing that total RPA32 and CHK1 expression is upregulated in mPTCL and human PTCL cell lines, which are potential indicators of a replication stress phenotype.

While we looked for direct evidence of ATR pathway activation, ATR protein was not constitutively activated in mPTCL relative to control spleens.

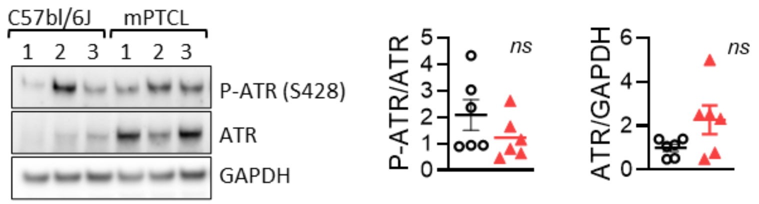

Phosphorylated CHK1 (S345) or RPA32 (S33) were also variable across control and mPTCL spleens, however we detected signaling activation in purified mPTCL tumor samples versus CD4 T-cell controls. Importantly, we found a **consistent upregulation of RPA32 and CHK1** in mPTCL splenocytes and purified tumor cells, which may indicate an accumulation of ssDNA and a greater reliance on ATR-CHK1 pathway (Forment and O'Connor, 2018). An analysis of CHK1 and RPA32 in spleens is shown in Fig. 6H.

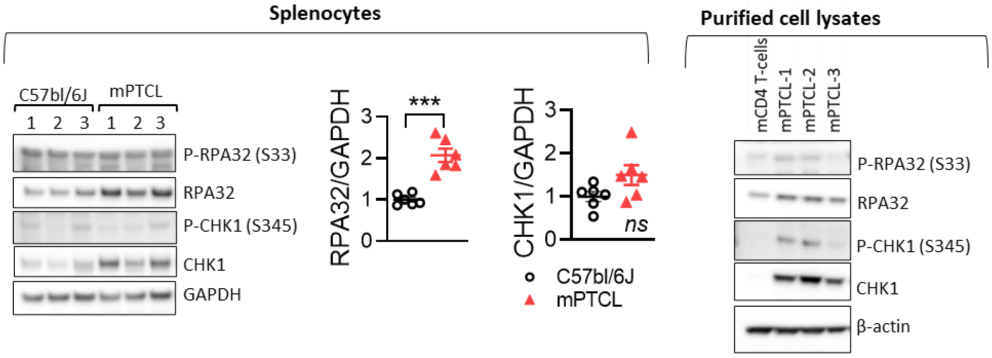

We have also performed similar analysis on human PTCL cell lines SU-DHL-1 and KARPAS-299, in parallel with two closely related T-ALL cell lines (all AZD6738-sensitive; see Fig. 7B). **PTCL cell lines also exhibited high baseline elevated CHK1 and RPA32** compared to NSCLC cell lines, whereas phosphorylated levels were not consistently elevated in ATR inhibitor-sensitive PTCL cell lines.

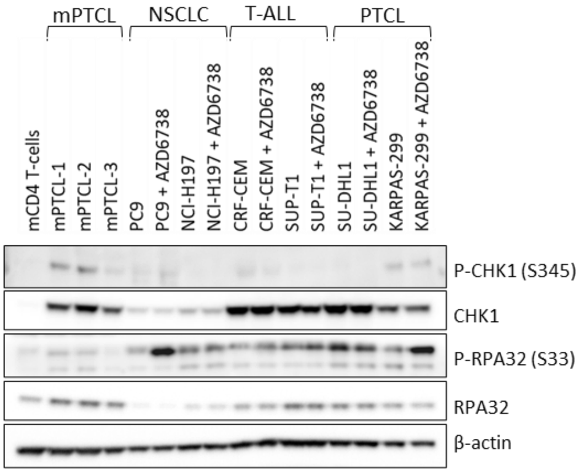

We extended and confirmed these findings by analyzing a CCLE RNAseq dataset, which we have included in Fig S6A. The data in Fig. S6A shows that in addition to mPTCL, human T-cell lymphoma cell lines express higher levels of *CHEK1* and *RPA2* (RPA32) transcript relative to all other cancer cell lines. These data reinforces the concept that cell cycle stress phenotypes such as upregulation of ATR pathway genes may be a common feature of PTCL, providing further rationale for the use DDR inhibitors to treat this disease.

18. KARPAS 299 and SU-DHL-1 cells are ALK+ ALCL cell lines, which do not correspond to the PTCL cell type(s) that the mPTCL model more closely appears to resemble: PTCL-GATA3 or PTCL-TFH. The therapeutic significance of their studies would be considerably improved if they

managed to use a human PDX (e.g. from PROXe) or patient-derived cell lines (e.g. see Am J Hematol 2017 Dec;92(12):1287-1294) closer to these entities (or at least more representative of the heterogeneity of PTCL in humans) for their in vivo and/or in vitro drug-related studies. Performing the studies indicated in 4) would likely require getting PDX or patient-derived cell lines from external sources and then performing at least in vitro experiments (quick) or in vivo studies (which may take up to a couple of months). Altogether, at least 2-4 months would be expectable to address this point, which nonetheless would be highly significant in broadening the potential translational/clinical relevance of the manuscript. The other 3 critiques can be addressed in a considerably shorter period of time.

Author comment 18: The reviewer is correct that ALK+ ALCL lines do not directly correspond to the PTCL subtype in the work. We nonetheless consider that the analysis of these related tumor lines adds value by enabling us to benchmark the model (Fig. 7C).

We acknowledge that exploring PTCL-GATA3 or PTCL-TFH PDX and/or PDC models would add greatly to the understanding of PTCL disease. However, these models are not readily available, either from the PROXe database, commercial sources, or through our network of collaborators, thus precluding our ability to perform the proposed experiments. This is in agreement with the profound lack of availability of preclinical models of PTCL-GATA3 and supports need of new models such as the mPTCL described in this work.

Minor comments:

19. Why were differences considered statistically significant for $P=0.05$? This is an unusual criterion that should be properly justified or changed to $P<0.05$ and data reanalyzed accordingly.

Author comment 19: This has been amended throughout the new version of the manuscript.

20. Please indicate the number mice/replicates analyzed in the IHC data presented throughout the manuscript.

Author comment 20: This information has been added to figure legends throughout the revised manuscript.

21. The authors show clearly in Fig 2 that, after transfer, the tumor that arises is a PTCL. However, the way in which the main text is written regarding the neoplasia in the original mouse and establishment of the PTCL line in the first section of the results ("Isolation of an in vivo

transplantable murine lymphoma") and how Fig 1A and Supp Fig 1 were formulated are confusing.

Author comment 21: We have revised Fig. 1A and the description of Figs. 1A and S1 in the text to improve clarity (Results section "**Isolation of an in vivo transplantable murine lymphoma**", paragraph 2).

22. Was the mouse #2695 (from which the splenocytes were collected that eventually originated the line in B6 mice) originally catalogued as displaying ABC DLBCL? From Fig S1 it is dubious if the mouse had lymphoma or not (mere B-cell hyperplasia leading to splenomegaly?) but it seems not (based on the color code used for mBCL and mPTCL), whereas from Fig 1A it seems probably yes (since the donor mouse, 2695, from which the spleen is collected is referred to as has having a "lymphoma" - of which type?). Please clarify and reformulate text and figures to make this clear. This is not mere semantics, since it is important to understand whether the mPTCL originated from a minor population of bystander T cells that were present in an otherwise DLBCL or B-cell "hyperplasia" in the original mouse or whether this mouse was an exception that developed already bystander PTCL (or T cell proliferative disease) originally.

Author comment 22: Mouse #2695 has the *Cγ1*-Cre *Blimp1*$^{fl/fl}$ genotype which is known to develop splenomegaly and B-cell hyperplasia resembling features of ABC-DLBCL (Calado et al., 2010). Cells were passaged into B6 host mice to establish transplantable lymphoma models. No further analysis had been performed on the original mouse #2695, and it was not confirmed whether this mouse harbored a true ABC DLBCL.

Fig. 2F & Fig. 2G of the originally submitted manuscript characterize the presence of the Cg1-Cre and un-excised *Blimp1*$^{fl/fl}$ allele, which serves as a traceable genetic biomarker that confirms the cell of origin was mouse #2695. We have amended the results section "**Isolation of an in vivo transplantable murine lymphoma**", paragraph 2, and section "**Characterization of Cγ1-Cre Blimp1fl/fl-derived T-cell transplantable lymphoma**" paragraph 1 to improve clarity.

It is an interesting question raised by the reviewer as to whether mouse #2695 harbored a significantly expanded population of mPTCL prior to its transplantation and/or whether the mPTCL developed in concert with hyperplastic B-cells. Unfortunately, we do not have the necessary samples from the original donor mouse to address this question. Moreover, a definitive answer would require longitudinal samples from the mouse.

23. Also, clarify in the legend what the grey x black colors mean in the mice depicted in Fig 1A.

Author comment 23: Fig. 1A has been amended in the revised manuscript to improve clarity.

24. For better clarity, instead of columns and (possibly) standard deviation (or is it standard error of mean?) in Figs such as 1G, 3A,D,E,H,I or 4C, the authors should present data as dot plots.

Author comment 24: The suggested amendments have been made in the revised manuscript.

25. In Fig 1B, although the authors do not make any claims regarding number of transplanted cells and disease 'kinetics', please indicate whether the survival curves for the different transplant cell numbers were statistically different or not. Either way the information is important.

Author comment 25: Statistical analysis has been added to Fig 1B.

26. Line 194: where it is "Fig S1D" it should be, I believe, "Fig. S2C".

Author comment 26: We thank the reviewer for identifying this error, which has been corrected in the revised manuscript.

27. mBCL arising in the context of mPTCL appears to be of Blimp1 wild type origin. So, the mBCL cells should be CD45.1. Was this the case?

Author comment 27: The mBCL lymphoma lineage was not passage through a CD45.1 host strain, and the mBCL tumors are therefore CD45.2 positive.

28. The identity of all the 3 PTCL cell lines should be indicated.

Author comment 28: This information has been added to the figure legend of Fig. 7A. The PTCL cell lines are DEL, KARPAS-299, SUP-M2.

29. In the main text the authors mention they tested 6 T-ALL cell lines but the corresponding figure (Fig B) only shows 5 cell lines. Please correct main text or include the missing cell line in the figure.

Author comment 29: We thank the reviewer for identifying this error, the text has been corrected (results section "**ATR is a therapeutic target in PTCL**", paragraph 3.

Reviewer #3 (Significance (Required)):

The significance of the studies presented in the manuscript by Kuczynski et al lies mainly on 3 aspects:

1) the generation (and deep characterization) of a novel in vivo model of PTCL, which may help advance the understanding of the underlying biology while providing a tool for translational studies. The authors provide good evidence that the model recapitulates several features of human PTCL, and particularly of PTCL-GATA3. The existence of other models, including of PTCL-GATA3, does not, in my view, decrease the relevance of the current studies, since the strategies to generate them are considerably different and thus the information on etiology of the disease and its heterogeneity will be complementary to previous studies.

2) the demonstration that replication stress occurs in the mPTCL (which should be made more robust and complemented with data from human PTCL but is exciting)

3) the demonstration that replication stress can be exploited therapeutically by the use of small molecule inhibitors of ATR and CHK1/2. This, if properly complemented with stronger human data, not only validates the importance of the model for human PTCL but is in itself a potentially major step forward for the targeting of PTCL cells.
Given its characteristics, the model may be especially proper also for further characterization of the crosstalk between GC B cells and Tfh cells in their ability to drive mature B and T cell lymphomas.
Overall, the studies should be of interest to both basic researchers and clinicians working in PTCL in particular, and also DLBCL, as well as other hematological malignancies.

Reviewer expertise: T-cell leukemia; signaling; mouse models

Review COMMONS

References:

Calado, D. P., Zhang, B., Srinivasan, L., Sasaki, Y., Seagal, J., Unitt, C., Rodig, S., Kutok, J., Tarakhovsky, A., Schmidt-Supprian, M., and Rajewsky, K. (2010). Constitutive canonical NF-kappaB activation cooperates with disruption of BLIMP1 in the pathogenesis of activated B cell-like diffuse large cell lymphoma. Cancer cell *18*, 580-589.

Cortes, J. R., Ambesi-Impiombato, A., Couronné, L., Quinn, S. A., Kim, C. S., da Silva Almeida, A. C., West, Z., Belver, L., Martin, M. S., Scourzic, L.*, et al.* (2018). RHOA G17V Induces T Follicular Helper Cell Specification and Promotes Lymphomagenesis. Cancer cell *33*, 259-273.e257.

Dominguez-Sola, D., Ying, C. Y., Grandori, C., Ruggiero, L., Chen, B., Li, M., Galloway, D. A., Gu, W., Gautier, J., and Dalla-Favera, R. (2007). Non-transcriptional control of DNA replication by c-Myc. Nature *448*, 445-451.

Forment, J. V., and O'Connor, M. J. (2018). Targeting the replication stress response in cancer. Pharmacology & therapeutics *188*, 155-167.

Iacobucci, I., Di Rorà, A. G. L., Falzacappa, M. V. V., Agostinelli, C., Derenzini, E., Ferrari, A., Papayannidis, C., Lonetti, A., Righi, S., Imbrogno, E.*, et al.* (2015). In vitro and in vivo single-agent efficacy of checkpoint kinase inhibition in acute lymphoblastic leukemia. Journal of Hematology & Oncology *8*, 125.

Kotsantis, P., Petermann, E., and Boulton, S. J. (2018). Mechanisms of Oncogene-Induced Replication Stress: Jigsaw Falling into Place. Cancer Discov *8*, 537-555.

Pechloff, K., Holch, J., Ferch, U., Schweneker, M., Brunner, K., Kremer, M., Sparwasser, T., Quintanilla-Martinez, L., Zimber-Strobl, U., Streubel, B.*, et al.* (2010). The fusion kinase ITK-SYK mimics a T cell receptor signal and drives oncogenesis in conditional mouse models of peripheral T cell lymphoma. J Exp Med *207*, 1031-1044.

Sarmento, L. M., Póvoa, V., Nascimento, R., Real, G., Antunes, I., Martins, L. R., Moita, C., Alves, P. M., Abecasis, M., Moita, L. F.*, et al.* (2015). CHK1 overexpression in T-cell acute lymphoblastic leukemia is essential for proliferation and survival by preventing excessive replication stress. Oncogene *34*, 2978-2990.

Toledo, L. I., Altmeyer, M., Rask, M. B., Lukas, C., Larsen, D. H., Povlsen, L. K., Bekker-Jensen, S., Mailand, N., Bartek, J., and Lukas, J. (2013). ATR prohibits replication catastrophe by preventing global exhaustion of RPA. Cell *155*, 1088-1103.

van Dongen, J. J., Langerak, A. W., Brüggemann, M., Evans, P. A., Hummel, M., Lavender, F. L., Delabesse, E., Davi, F., Schuuring, E., García-Sanz, R.*, et al.* (2003). Design and standardization of PCR primers and protocols for detection of clonal immunoglobulin and T-cell receptor gene recombinations in suspect lymphoproliferations: report of the BIOMED-2 Concerted Action BMH4-CT98-3936. Leukemia *17*, 2257-2317.

Yap, T. A., O'Carrigan, B., Penney, M. S., Lim, J. S., Brown, J. S., de Miguel Luken, M. J., Tunariu, N., Perez-Lopez, R., Rodrigues, D. N., Riisnaes, R.*, et al.* (2020). Phase I Trial of First-in-Class ATR Inhibitor M6620 (VX-970) as Monotherapy or in Combination With Carboplatin in Patients With Advanced Solid Tumors. J Clin Oncol *38*, 3195-3204.

Yap, T. A., Tan, D. S. P., Terbuch, A., Caldwell, R., Guo, C., Goh, B. C., Heong, V., Haris, N. R. M., Bashir, S., Drew, Y.*, et al.* (2021). First-in-Human Trial of the Oral Ataxia Telangiectasia and RAD3-Related (ATR) Inhibitor BAY 1895344 in Patients with Advanced Solid Tumors. Cancer Discov *11*, 80-91.

17th Feb 2022

Dear Dr. Kuczynski,

Thank you for the submission of your manuscript to EMBO Molecular Medicine. We have now received the enclosed reports from the two referees who were invited to review your manuscript (and who had initially reviewed it for Review Commons). As you will see, they are supportive of publication pending minor revisions, and I am therefore pleased to inform you that we will be able to accept your manuscript once the following points will be addressed:

1/ Referees' comments: please address the remaining concerns from referee #1 (previously referee #3) and referee #2 (previously referee #2).

2/ Main manuscript text:
- Please address the queries from our data editors in track changes mode in the main manuscript file labelled 'Data edited MS file'. Please use this file for any further modification (in track changes mode).
- Please provide up to 5 keywords.
- Kindly place the Competing Interests section after the Author Contributions and rename it Disclosure Statement and Competing Interests. We updated our journal's competing interests policy in January 2022 and request authors to consider both actual and perceived competing interests. Please review the policy https://www.embopress.org/competing-interests and update your competing interests if necessary.
- We do not have abbreviations list, please rather introduce each abbreviation when it is first used in the text.
- Please remove Data not shown (p.10 and p.16). As per our guidelines on "Unpublished Data", all data referred to in the paper should be displayed in the main or Expanded View figures.
- Material and Methods: This section should be placed after the Discussion. Please also include in this section your current Supplemental Materials and Methods.
o Animal models: please indicate the age and gender of the mice. Please indicate the housing and husbandry conditions.
o Flow cytometry: please define "standard methods" or include a reference.
o Antibodies: please provide the dilutions used for all experiments.
o Cells: please indicate the origin of the cells, their culture conditions, and whether they were authenticated and tested for mycoplasma contamination.
- Data availability section: It is mandatory to include a 'Data Availability' section after the Materials and Methods. Before submitting your revision, primary datasets produced in this study must be deposited in an appropriate public database, and the accession numbers and database listed under 'Data Availability'. Please remember to provide a reviewer password if the datasets are not yet public (https://www.embopress.org/page/journal/17574684/authorguide#dataavailability). Note that the Data Availability Section is restricted to new primary data that are part of this study.
- Acknowledgements: All funding information provided in the manuscript should be entered in the submission system.
- References: Please list the references alphabetically, with 10 authors before et al.

3/ Figures and Appendix:
- Please upload your figures individually as .eps, .tif, .jpg (one file per figure).
- Please provide in the figures or in their legends the exact p values, not a range. Some people found that to keep the figures clear, providing a supplemental table in the Appendix with all exact p-values was preferable. You are welcome to do this if you want to.
- The supplementary Tables should be renamed Dataset EV1-7. Their legends should be removed from the manuscript and added to the table files in a separate tab.
- Appendix: The 6 supplementary figures should be in one Appendix file with a Table of Content, and each figure renamed Appendix figure S1-S6. The legends should be removed from the manuscript file and added to the Appendix file. Please note that you have the possibility to have Expanded View (EV) Figures that are collapsible/expandable online. A maximum of 5 EV Figures can be typeset. EV Figures should be cited as 'Figure EV1, Figure EV2" etc... in the text and their respective legends should be included in the main text after the legends of regular figures.

4/ Please provide a complete author checklist, which you can download from our author guidelines (https://www.embopress.org/page/journal/17574684/authorguide#submissionofrevisions). Please insert information in the checklist that is also reflected in the manuscript. The completed author checklist will also be part of the RPF.

5/ We would also encourage you to include the source data for figure panels that show essential data. Numerical data should be provided as individual .xls or .csv files (including a tab describing the data). For blots or microscopy, uncropped images should be submitted (using a zip archive if multiple images need to be supplied for one panel). Additional information on source data and instruction on how to label the files are available at
.

6/ Please include "The Paper Explained" in the manuscript: EMBO Molecular Medicine articles are accompanied by a summary of the articles to emphasize the major findings in the paper and their medical implications for the non-specialist reader. Please provide a draft summary of your article highlighting
- the medical issue you are addressing,
- the results obtained and
- their clinical impact.
This may be edited to ensure that readers understand the significance and context of the research. Please refer to any of our published articles for an example.

7/ Every published paper now includes a 'Synopsis' to further enhance discoverability. Synopses are displayed on the journal webpage and are freely accessible to all readers. They include a short stand first (maximum of 300 characters, including space) as well as 2-5 one-sentences bullet points that summarizes the paper. Please write the bullet points to summarize the key NEW findings. They should be designed to be complementary to the abstract - i.e. not repeat the same text. We encourage inclusion of key acronyms and quantitative information (maximum of 30 words / bullet point). Please use the passive voice. Please attach these in a separate file or send them by email, we will incorporate them accordingly.

Please also suggest a striking image or visual abstract to illustrate your article as a PNG file 550 px wide x 300-600 px high.

8/ As part of the EMBO Publications transparent editorial process initiative (see our Editorial at http://embomolmed.embopress.org/content/2/9/329), EMBO Molecular Medicine will publish online a Review Process File (RPF) to accompany accepted manuscripts.
This file will be published in conjunction with your paper and will include the anonymous referee reports, your point-by-point response and all pertinent correspondence relating to the manuscript. Let us know whether you agree with the publication of the RPF and as here, if you want to remove or not any figures from it prior to publication.
Please note that the Authors checklist will be published at the end of the RPF.

I look forward to receiving your revised manuscript.

Yours sincerely,

Lise Roth

Lise Roth, PhD
Editor
EMBO Molecular Medicine

You can submit your revised files by logging onto our online manuscript tracking system or simply follow this link:

Link Not Available

Please do not share this URL as it will give anyone who clicks it access to your account.

***** Reviewer's comments *****

Referee #1 (Remarks for Author):

The authors have properly addressed most of my previous criticisms and have adequately justified why they were not able to perform all the suggested experiments. The manuscript has been clearly improved and I congratulate them for their work.

Minor issues that remain:

1) The authors do not wish to make strong claims on Tfh cells being the cell of origin for their lymphomas. This is wise, given the lack of robust data supporting this. In addition, they should discuss the fact that although they do present some evidence in support of "a potential Tfh cell lymphoma origin" they also present some evidence against this possibility, namely the fact that the lymphoma cells do not express CXCR5.

2) In the revised Fig. S3C, the histograms show that the majority of the cells in Fig S3C are positive for PD1 and also for ICOS, and thus the conclusion of the authors that there is concomitant expression of PD1 and ICOS is, by and large, fair. However, the authors should show, in addition to the histogram overlays, a contour or dot plot with both markers (ideally for each of the three time points). This would clarify, for example, whether the few cells that are ICOS negative are also PD1 negative or not.

3) Since the authors use T-ALL cell lines as "reference" in their inhibitor studies, they should properly contextualize and cite the studies on the use of CHK1/ATR pathway inhibitors in T-ALL (which they mention in their response to Reviewer#2) in the manuscript.

Referee #2 (Remarks for Author):

Many thanks for the clear responses and the changes in the manuscript. However, one point is still unclear to me or misunderstood. There is significant emphasis on the fact that clonal T-cell with an expression of Vß8 segment are present in the lymphomas. However the data presented for clonotype detection via NGS (figure 2C) display no such predominance of Vß8 rearrangements. I would expect that the pronounced expression of Vß8 segments should be based on a respective TCRß rearrangement involving a Vß8 gene segment. Could you please clarify this?

***

Rev_Com_number: RC-2021-00947
New_manu_number: EMM-2022-15816
Corr_author: Kuczynski
Title: A preclinical model of peripheral T-cell lymphoma GATA3 reveals DNA damage response pathway vulnerability

Referee comments: Reviewer #1:

1) The authors do not wish to make strong claims on Tfh cells being the cell of origin for their lymphomas. This is wise, given the lack of robust data supporting this. In addition, they should discuss the fact that although they do present some evidence in support of "a potential Tfh cell lymphoma origin" they also present some evidence against this possibility, namely the fact that the lymphoma cells do not express CXCR5.

   Author comment: We have clarified the text in the Results section (heading: 'mPTCL phenotypically and functionally resemble T-follicular helper cells') that a Tfh cell origin was not definite due to an absence of CXCR5:

   'Immunostaining confirmed nuclear BCL6 expression throughout mPTCL-infiltrated spleens (**Fig. 3B**). Thus tumor cells expressed several markers of normal Tfh cells. The homogeneity of PD1 and ICOS on tumor cells and their maintained expression throughout passaging (Fig. **S3A**) suggested possible Tfh cell origin. However, since tumor cells were negative for Tfh chemokine receptor CXCR5 (Figs. **S3B, C**), a definitive Tfh origin could not be concluded.'

2) In the revised Fig. S3C, the histograms show that the majority of the cells in Fig S3C are positive for PD1 and also for ICOS, and thus the conclusion of the authors that there is concomitant expression of PD1 and ICOS is, by and large, fair. However, the authors should show, in addition to the histogram overlays, a contour or dot plot with both markers (ideally for each of the three time points). This would clarify, for example, whether the few cells that are ICOS negative are also PD1 negative or not.

   Author comment: This is a helpful suggestion made by the reviewer. We have added the suggested contour plots to the Supplemental Fig. which reinforce that lymphoma cells concomitantly express high levels of both ICOS and PD1.

   Below we show the contour plots of the three tumor passages depicted in Supplemental Fig. S3 (now Supplemental Fig. S3A) as requested by the reviewer. These data highlight concomitant expression of PD1 and ICOS, however a small subset of PD1- and ICOS- cells can also be observed (which the reviewer has also remarked upon based on the histograms). These ICOS-PD1- cells are normal T-cells -note that total T-cells were gated in this study. Indeed, the PD1+ICOS+ cells are Ki67-positive while PD1-ICOS- cells are largely Ki67-.

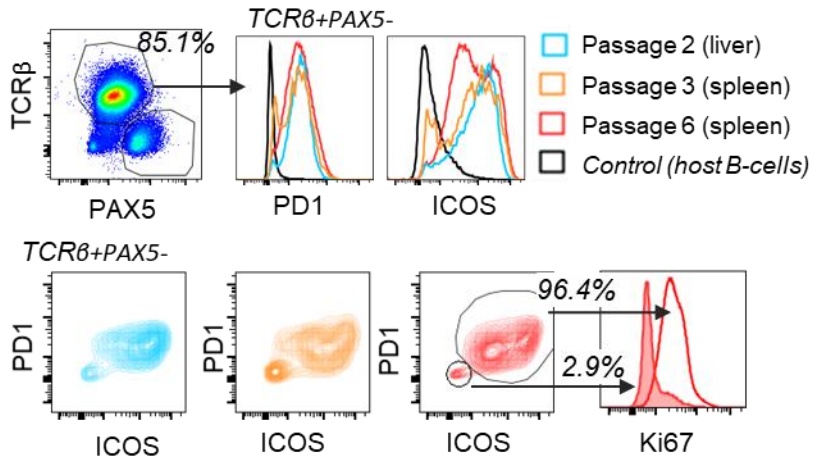

We propose instead to show contour plots of lymphoma cells passaged in congenic CD45.1 hosts. This will address the comment of the reviewer while avoiding misleading readers to think incorrectly that a subset of lymphoma cells do not express PD1 or ICOS. These new data are below, and have been added as a new panel in *Supplemental Figure S3B*. By specifically gating for lymphoma cells (CD45.2+), a single population of PD1+ICOS+ cells are observed, and normal T-cells do not confound the expression patterns.

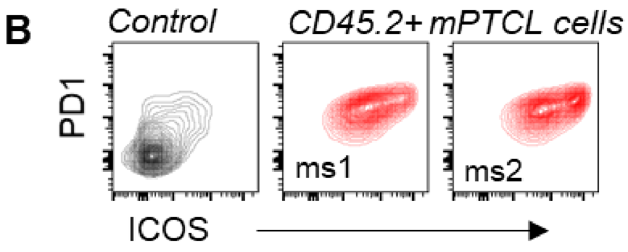

3) Since the authors use T-ALL cell lines as "reference" in their inhibitor studies, they should properly contextualize and cite the studies on the use of CHK1/ATR pathway inhibitors in T-ALL (which they mention in their response to Reviewer#2) in the manuscript.

Author comment: We have now included a brief discussion of T-ALL sensitivity to DNA damage response inhibitors under *Discussion, 3rd paragraph from the end.*

'Normal antigen-activated T cells display extremely high proliferation rates, signs of DNA damage (such as γH2AX induction) and a DDR (McNally et al, 2017). Moreover, immature T-cell leukemia (T-ALL) cell lines were highly sensitive to single agent and combination treatment with DDR inhibitors WEE1, CHK1 and ATR (Ghelli Luserna Di Rorà et al, 2019; Iacobucci et al, 2015). A CHK1 inhibitor (PF-0477736) significantly prolonged survival of mice implanted with a mutagen-induced murine lymphoid T-cell leukemia (Iacobucci et al., 2015). These data suggest a potential inherent sensitivity of proliferating T cells to DDR inhibitors which might also be a vulnerability of PTCL.'

Referee comments: Reviewer #2:

4) Many thanks for the clear responses and the changes in the manuscript. However, one point is still unclear to me or misunderstood. There is significant emphasis on the fact that clonal T-cell with an expression of Vß8 segment are present in the lymphomas. However the data presented for clonotype detection via NGS (figure 2C) display no such predominance of Vß8 rearrangements. I would expect that the pronounced expression of Vß8 segments should be based on a respective TCRß rearrangement involving a Vß8 gene segment. Could you please clarify this?

Author comment: We apologize for further confusion surrounding the nature of the T-cell monoclonal origin and expansion of vb8+ clones. We think that a possible source of this confusion lies in the naming convention for TCRβ variable genes vs. proteins in mouse. The expanded protein clonotype identified by flow cytometry (Fig. 2C) is vβ8 (8.1 or 8.2). However the corresponding gene for vβ8.2 is *Tcbv13-2*. Therefore the protein (TCRvβ8) and NGS (*Tcbv13-2)* data are in agreement, and equally show predominance of a single clone in the lymphoma.

While the vβ8 gene nomenclature was previously noted in the *Results* section, we have now also added this remark in the Fig. 2D legend for the TCR sequencing result:

‘**Fig. 2D.** T-cell transcript complementary determining region 3 (CDR3) sequencing of C57bl/6J (top right) and lymphoma-bearing spleen. Chord diagram shows numbers of distinct TRBV and TRBJ clones. The dominant CDR3 sequence in the lymphoma is CASGDTQANSDYTF. Note that Trbv13-2 gene encodes TCRvβ8.2 protein.’

Other:

1. Keywords have been added to p.1
2. The *Disclosure Statement* has been placed after Acknowledgements
3. The list of abbreviations has been removed.
4. The reference to ‘data not shown’ on p.10 has been deleted. ‘Data not shown’ from p.16 can now be found in Supplemental Figure S6A, showing no relationship between phosphorylated RPA32, CHK1 and ATR levels and the presence of mPTCL. Note that this data was previously reported in Author Comment #17 in response to Reviewer #3, but was not added to the manuscript during the last revision.
5. *Materials and Methods* have been placed after the *Discussion*. *Supplemental Materials and Methods* have also been placed here.
6. The sex and age of mice used in studies and the housing condition of animals has been defined in *Materials and Methods: In vivo studies.*
7. The methods for flow cytometry have been defined.
8. All antibody dilutions have been uploaded as a chart in *Supplementary Materials and Methods*.
9. Details on cell line origin, culture conditions and authentication/mycoplasma testing have been added to *Supplemental Materials and Methods: In Vitro Cultures*
10. Primary RNA and exome sequencing data have been uploaded on the European Nucleotide Archive (accession number PRJEB51324). Details have been added under *Materials and Methods: Data availability.*
11. Funding information has been removed from *Acknowledgements* in the manuscript file.
12. *EMBO Mol Med* reference style has been applied to the manuscript
13. The supplementary Tables have been renamed and legends inserted as a separate tab.
14. The six supplemental Figures with legends have been placed in an Appendix file. Three expanded view figures (EV1,2,3) have been proposed. These are Supplemental/Appendix Figures S1, S3, S6. Both figure citations have been inserted as a placeholder in the Manuscript file text.
15. The author checklist is completed
16. Source data in Supplemental/Datasets Tables 1-7 have already been supplied and further source data can be made available upon request.
17. A *Synopsis* and *Paper Explained* section have been added as an attachment file
18. A visual abstract has been supplied.

END

1st Revision - Editorial Decision 28th Mar 2022

28th Mar 2022

Dear Dr. Kuczynski,

Thank you for the submission of your revised manuscript to EMBO Molecular Medicine. I am pleased to inform you that we will be able to accept your manuscript once the following minor editorial points will be addressed:

- Please make sure to address all queries from our data editors in track changes mode in the file labelled 'Data edited MS file'.
- Please merge Materials and Methods and Supplemental Materials and Methods and remove the mention "Supplemental Materials and Methods". Please update the checklist accordingly.
- Data availability section: Please provide the URL to access the datasets in this section. Please note that the data must be made public before acceptance.
- Please make sure that the funding information in the submission system and in the manuscript match.
- Thank you for providing The Paper Explained, and a synopsis text and picture. Please include The Paper Explained in the main manuscript file and upload the synopsis figure individually as a png/tif/jpeg file 550 px wide x 300-600 px high. The text in the figure should remain legible.

I added minor modification to your synopsis text to fit our format, please let me know if you agree with the following:

A murine peripheral T-cell lymphoma (mPTCL) arose coincident to B-cell hyperplasia and was developed into a transplantable preclinical model. mPTCL and human PTCL lines responded to ATR inhibition, providing a rationale for clinical exploration of DNA-damage response inhibitors for PTCL treatment.

• A transplantable lymphoma arose in a Blimp1fl/flCγ1-Cre mouse strain that is predisposed to spontaneous B-cell hyperplasia.
• The lymphoma originated from a T-cell progenitor and modelled cellular phenotypic hallmarks of human PTCL-GATA3.
• The lymphoma bears a clonal Ctnnb1 1004A>C mutation encoding a K335T substitution in β-catenin, which is found in 6% of human PTCLs.
• mPTCL exhibited upregulation of β-catenin and the downstream target Myc, together with biomarkers of DNA replication stress, and responded to the ATR inhibitor Ceralasertib in vivo.
• Ceralasertib reduced proliferation of human PTCL cell lines, supporting a rationale for future clinical exploration of ATR inhibitors for the treatment of PTCL patients.

As part of the EMBO Publications transparent editorial process initiative (see our Editorial at http://embomolmed.embopress.org/content/2/9/329), EMBO Molecular Medicine will publish online a Review Process File (RPF) to accompany accepted manuscripts.
This file will be published in conjunction with your paper and will include the anonymous referee reports, your point-by-point response and all pertinent correspondence relating to the manuscript. Let us know whether you agree with the publication of the RPF and as here, if you want to remove or not any figures from it prior to publication.
Please note that the Authors checklist will be published at the end of the RPF.

I look forward to receiving your revised manuscript at your earliest convenience.

With kind regards,

Lise Roth

Lise Roth, PhD
Editor
EMBO Molecular Medicine

To submit your manuscript, please follow this link:

Link Not Available

***
Rev_Com_number: RC-2021-00947
New_manu_number: EMM-2022-15816-V2
Corr_author: Kuczynski

The authors performed the requested editorial changes.

7th Apr 2022

Dear Dr. Kuczynski,

Thank you for submitting your revised files. I am pleased to inform you that your manuscript is accepted for publication and is now being sent to our publisher to be included in the next available issue of EMBO Molecular Medicine!

Please read below for additional IMPORTANT information regarding your article, its publication and the production process.

Congratulations on your interesting work!

With kind regards,

Lise Roth

Lise Roth, Ph.D
Scientific Editor
EMBO Molecular Medicine

Follow us on Twitter @EmboMolMed

EMBO Press Author Checklist

Corresponding Authors: Elizabeth Kuczynski, Larissa Carnevalli, Dinis Calado, Charles Sinclair
Journal Submitted to: EMBO Molecular Medicine
Manuscript Number: EMM-2022-15816

USEFUL LINKS FOR COMPLETING THIS FORM
The EMBO Journal - Author Guidelines
EMBO Reports - Author Guidelines
Molecular Systems Biology - Author Guidelines
EMBO Molecular Medicine - Author Guidelines

Reporting Checklist for Life Science Articles (updated January 2022)
This checklist is adapted from Materials Design Analysis Reporting (MDAR) Checklist for Authors. MDAR establishes a minimum set of requirements in transparent reporting in the life sciences (see Statement of Task: 10.31222/osf.io/9sm4x). Please follow the journal's guidelines in preparing your manuscript.
Please note that a copy of this checklist will be published alongside your article.

Abridged guidelines for figures
1. Data
The data shown in figures should satisfy the following conditions:
- the data were obtained and processed according to the field's best practice and are presented to reflect the results of the experiments in an accurate and unbiased manner.
- ideally, figure panels should include only measurements that are directly comparable to each other and obtained with the same assay.
- plots include clearly labeled error bars for independent experiments and sample sizes. Unless justified, error bars should not be shown for technical replicates.
- if n<5, the individual data points from each experiment should be plotted. Any statistical test employed should be justified.
- Source Data should be included to report the data underlying figures according to the guidelines set out in the authorship guidelines on Data Presentation.

2. Captions
Each figure caption should contain the following information, for each panel where they are relevant:
- a specification of the experimental system investigated (eg cell line, species name).
- the assay(s) and method(s) used to carry out the reported observations and measurements.
- an explicit mention of the biological and chemical entity(ies) that are being measured.
- an explicit mention of the biological and chemical entity(ies) that are altered/varied/perturbed in a controlled manner.
- the exact sample size (n) for each experimental group/condition, given as a number, not a range;
- a description of the sample collection allowing the reader to understand whether the samples represent technical or biological replicates (including how many animals, litters, cultures, etc.).
- a statement of how many times the experiment shown was independently replicated in the laboratory.
- definitions of statistical methods and measures:
  - common tests, such as t-test (please specify whether paired vs. unpaired), simple χ2 tests, Wilcoxon and Mann-Whitney tests, can be unambiguously identified by name only, but more complex techniques should be described in the methods section;
  - are tests one-sided or two-sided?
  - are there adjustments for multiple comparisons?
  - exact statistical test results, e.g., P values = x but not P values < x;
  - definition of 'center values' as median or average;
  - definition of error bars as s.d. or s.e.m.

Please complete ALL of the questions below.
Select "Not Applicable" only when the requested information is not relevant for your study.

Materials

Newly Created Materials	Information included in the manuscript?	In which section is the information available? (Reagents and Tools Table, Materials and Methods, Figures, Data Availability Section)
New materials and reagents need to be available; do any restrictions apply?	Yes	Materials and Methods

Antibodies	Information included in the manuscript?	In which section is the information available? (Reagents and Tools Table, Materials and Methods, Figures, Data Availability Section)
For **antibodies** provide the following information:<br>- Commercial antibodies: RRID (if possible) or supplier name, catalogue number and or/clone number<br>- Non-commercial: RRID or citation	Yes	Materials and Methods

DNA and RNA sequences	Information included in the manuscript?	In which section is the information available? (Reagents and Tools Table, Materials and Methods, Figures, Data Availability Section)
**Short novel DNA or RNA including primers, probes**: provide the sequences.	Yes	Materials and Methods

Cell materials	Information included in the manuscript?	In which section is the information available? (Reagents and Tools Table, Materials and Methods, Figures, Data Availability Section)
**Cell lines:** Provide species information, strain. Provide accession number in repository **OR** supplier name, catalog number, clone number, and/**OR** RRID.	Yes	Materials and Methods
**Primary cultures:** Provide species, strain, sex of origin, genetic modification status.	Not Applicable	
Report if the cell lines were recently **authenticated** (e.g., by STR profiling) and tested for mycoplasma contamination.	Yes	Materials and Methods

Experimental animals	Information included in the manuscript?	In which section is the information available? (Reagents and Tools Table, Materials and Methods, Figures, Data Availability Section)
**Laboratory animals or Model organisms:** Provide species, strain, sex, age, genetic modification status. Provide accession number in repository **OR** supplier name, catalog number, clone number, **OR** RRID.	Yes	Materials and Methods
**Animal observed in or captured from the field:** Provide species, sex, and age where possible.	Not Applicable	
Please detail **housing and husbandry conditions**.	Yes	Materials and Methods

Plants and microbes	Information included in the manuscript?	In which section is the information available? (Reagents and Tools Table, Materials and Methods, Figures, Data Availability Section)
**Plants:** provide species and strain, ecotype and cultivar where relevant, unique accession number if available, and source (including location for collected wild specimens).	Not Applicable	
**Microbes:** provide species and strain, unique accession number if available, and source.	Not Applicable	

Human research participants	Information included in the manuscript?	In which section is the information available? (Reagents and Tools Table, Materials and Methods, Figures, Data Availability Section)
If collected and within the bounds of privacy constraints report on age, sex and gender or ethnicity for all study participants.	Not Applicable	

Core facilities	Information included in the manuscript?	In which section is the information available? (Reagents and Tools Table, Materials and Methods, Figures, Data Availability Section)
If your work benefited from core facilities, was their service mentioned in the acknowledgments section?	Yes	Acknowledgements

Design

Study protocol	Information included in the manuscript?	In which section is the information available? (Reagents and Tools Table, Materials and Methods, Figures, Data Availability Section)
If study protocol has been **pre-registered, provide DOI in the manuscript**. For clinical trials, provide the trial registration number **OR** cite DOI.	Not Applicable	
Report the **clinical trial registration number** (at ClinicalTrials.gov or equivalent), where applicable.	Not Applicable	

Laboratory protocol	Information included in the manuscript?	In which section is the information available? (Reagents and Tools Table, Materials and Methods, Figures, Data Availability Section)
Provide DOI OR other citation details if **external detailed step-by-step protocols** are available.	Not Applicable	

Experimental study design and statistics	Information included in the manuscript?	In which section is the information available? (Reagents and Tools Table, Materials and Methods, Figures, Data Availability Section)
Include a statement about **sample size** estimate even if no statistical methods were used.	Yes	Materials and Methods
Were any steps taken to minimize the effects of subjective bias when allocating animals/samples to treatment (e.g. **randomization procedure**)? If yes, have they been described?	Yes	Materials and Methods
Include a statement about **blinding** even if no blinding was done.	Yes	Materials and Methods
Describe **inclusion/exclusion criteria** if samples or animals were excluded from the analysis. Were the criteria pre-established?<br><br>If sample or data points were omitted from analysis, report if this was due to attrition or intentional exclusion and provide justification.	Yes	Materials and Methods
For every figure, are **statistical tests** justified as appropriate? Do the data meet the assumptions of the tests (e.g., normal distribution)? Describe any methods used to assess it. Is there an estimate of variation within each group of data? Is the variance similar between the groups that are being statistically compared?	Yes	Materials and Methods

Sample definition and in-laboratory replication	Information included in the manuscript?	In which section is the information available? (Reagents and Tools Table, Materials and Methods, Figures, Data Availability Section)
In the figure legends: state number of times the experiment was **replicated** in laboratory.	Yes	Figure legends
In the figure legends: define whether data describe **technical or biological replicates**.	Yes	Figure legends

Ethics

Ethics	Information included in the manuscript?	In which section is the information available? (Reagents and Tools Table, Materials and Methods, Figures, Data Availability Section)
Studies involving **human participants**: State details of **authority granting ethics approval** (IRB or equivalent committee(s), provide reference number for approval.	Not Applicable	
Studies involving **human participants**: Include a statement confirming that **informed consent** was obtained from all subjects and that the experiments conformed to the principles set out in the WMA Declaration of Helsinki and the Department of Health and Human Services Belmont Report.	Not Applicable	
Studies involving **human participants:** For publication of **patient photos**, include a statement confirming that consent to publish was obtained.	Not Applicable	
Studies involving experimental **animals**: State details of **authority granting ethics approval** (IRB or equivalent committee(s), provide reference number for approval. Include a statement of compliance with ethical regulations.	Yes	Materials and Methods
Studies involving **specimen and field samples:** State if relevant **permits** obtained, provide details of authority approving study; if none were required, explain why.	Not Applicable	

Dual Use Research of Concern (DURC)	Information included in the manuscript?	In which section is the information available? (Reagents and Tools Table, Materials and Methods, Figures, Data Availability Section)
Could your study fall under dual use research restrictions? Please check biosecurity documents and list of **select agents and toxins** (CDC): https://www.selectagents.gov/sat/list.htm	Not Applicable	
If you used a select agent, is the security level of the lab appropriate and reported in the manuscript?	Not Applicable	
If a study is subject to dual use research of concern regulations, is the name of the **authority granting approval and reference number** for the regulatory approval provided in the manuscript?	Not Applicable	

Reporting

The MDAR framework recommends adoption of discipline-specific guidelines, established and endorsed through community initiatives. Journals have their own policy about requiring specific guidelines and recommendations to complement MDAR.

Adherence to community standards	Information included in the manuscript?	In which section is the information available? (Reagents and Tools Table, Materials and Methods, Figures, Data Availability Section)
State if relevant guidelines or checklists (e.g., **ICMJE, MIBBI, ARRIVE, PRISMA**) have been followed or provided.	Yes	Materials and Methods
For **tumor marker prognostic studies,** we recommend that you follow the **REMARK** reporting guidelines (see link list at top right). See author guidelines, under 'Reporting Guidelines'. Please confirm you have followed these guidelines.	Not Applicable	
For **phase II and III randomized controlled trials**, please refer to the **CONSORT** flow diagram (see link list at top right) and submit the CONSORT checklist (see link list at top right) with your submission. See author guidelines, under 'Reporting Guidelines'. Please confirm you have submitted this list.	Not Applicable	

Data Availability

Data availability	Information included in the manuscript?	In which section is the information available? (Reagents and Tools Table, Materials and Methods, Figures, Data Availability Section)
Have **primary datasets** been deposited according to the journal's guidelines (see 'Data Deposition' section) and the respective accession numbers provided in the Data Availability Section?	Yes	Materials and Methods
Were **human clinical and genomic datasets** deposited in a public access-controlled repository in accordance to ethical obligations to the patients and to the applicable consent agreement?	Not Applicable	
Are **computational models** that are central and integral to a study available without restrictions in a machine-readable form? Were the relevant accession numbers or links provided?	Not Applicable	
If publicly available data were reused, provide the respective **data citations in the reference list**.	Yes	Materials and Methods
